# Combination of six enzymes of a marine *Novosphingobium* converts the stereoisomers of β-*O*-4 lignin model dimers into the respective monomers

**DOI:** 10.1038/srep15105

**Published:** 2015-10-19

**Authors:** Yukari Ohta, Shinro Nishi, Ryoichi Hasegawa, Yuji Hatada

**Affiliations:** 1 Japan Agency for Marine-Earth Science and Technology (JAMSTEC), 2-15 Natsushima, Yokosuka, Kanagawa, Japan

## Abstract

Lignin, an aromatic polymer of phenylpropane units joined predominantly by β-*O*-4 linkages, is the second most abundant biomass component on Earth. Despite the continuous discharge of terrestrially produced lignin into marine environments, few studies have examined lignin degradation by marine microorganisms. Here, we screened marine isolates for β-*O*-4 cleavage activity and determined the genes responsible for this enzymatic activity in one positive isolate. *Novosphingobium* sp. strain MBES04 converted all four stereoisomers of guaiacylglycerol-β-guaiacyl ether (GGGE), a structural mimic of lignin, to guaiacylhydroxypropanone as an end metabolite in three steps involving six enzymes, including a newly identified *Nu*-class glutathione-S-transferase (GST). *In silico* searches of the strain MBES04 genome revealed that four GGGE-metabolizing GST genes were arranged in a cluster. Transcriptome analysis demonstrated that the lignin model compounds GGGE and (2-methoxyphenoxy)hydroxypropiovanillone (MPHPV) enhanced the expression of genes in involved in energy metabolism, including aromatic-monomer assimilation, and evoked defense responses typically expressed upon exposure to toxic compounds. The findings from this study provide insight into previously unidentified bacterial enzymatic systems and the physiological acclimation of microbes associated with the biological transformation of lignin-containing materials in marine environments.

Lignin is the second most abundant organic carbon and is produced by terrestrial plants (15% to 40% dry weight) as an aromatic polymer of monomeric units joined predominantly by β-*O*-4 linkages (approximately 45%–60%)^1^,^2^. Lignin is also the major component of terrigenous organic carbon (TerrOC), which is discharged from rivers into marine environments at a rate of approximately 4.0 × 10^11^ kg (0.4 Gt)/year. Despite the enormous quantities of TerrOC that are annually deposited in the ocean, chemical biomarker and stable isotopic data from geochemical studies indicate that only low levels of TerrOC are present in the global ocean ecosystem^3^,^4^. Clarifying the conversion processes and flux of this “missing TerrOC” has been one of the major conundrums in oceanography in the past few decades^3^,^4^,^5^,^6^,^7^.

TerrOC is comprised of a large proportion of lignin-containing plant material, which is highly recalcitrant to chemical degradation^5^. In natural environments, lignin is predominantly degraded by members of the fungal class Agaricomycetes^8^, which are strictly aerobic and inactive in marine environments. Therefore, TerrOC is generally considered to be poorly degraded in marine environments. However, Loh *et al.*^9^ detected evidence of lignin metabolism in a Scottish sea sediment. In addition, a marked decrease of total lignin phenol content was observed in sediment samples collected from depths between 74 and 2250 m in the north-western Gulf of Mexico^10^. The efficient removal of low- and high-molecular weight lignin fractions in Arctic Ocean seawater with increasing depth has also been reported^6^,^11^. These recent findings provide evidence that a certain proportion of discharged terrigenous lignin is biologically degraded in sea water and marine sediments prior to diagenesis and storage of the highly condensed and recalcitrant products as unknown “humic” substances.

The findings from recent phylogenetic analysis^12^ of microbial flora associated with sunken-wood indicate that α-Proteobacteria often constitutes a major and ubiquitous group within sunken-wood microbial communities, particularly in the early degradation stages of wood components. In addition, these analyses have revealed that specialized metabolizing pathways exist for TerrOC in marine environments. Therefore, identifying the genes encoding the enzymes responsible for lignin decay in aquatic environments, including the ocean, is key to improving our understanding of global carbon cycling^3^.

Microbial extracellular metalloenzymes, such as peroxidases and laccases, are typically involved in the primary stages of bacterial lignin modification^13^,^14^,^15^. Functional screening of metagenomic scaffolds sourced from coal bed microbial communities suggests that lignin is transformed by the concerted activities of various environmental bacterial enzymes^16^. Sequence analyses of fosmids carrying lignin-transforming enzymes, including copper-containing oxidases, suggest that multidrug resistance pumps and regulatory genes are involved in the physiological acclimation of microbes upon exposure to lignin transformation products in the environment^16^.

Among previously conducted cultivation-dependent studies, *Sphingobium* sp. strain SYK-6 isolated from waterlogged waste sludge generated during paper production was shown to be capable of assimilating a wide range of lignin-related aromatic monomers and dimers^17^,^18^,^19^,^20^,^21^,^22^. Strain SYK-6 cleaves the β*-O-*4 ether linkage in lignin model dimers via multiple enzyme reactions. In the first cleavage step, as demonstrated using a lignin model dimer, short-chain dehydrogenases (SDRs) catalyze Cα dehydrogenation. The β*-O-*4 linkages between monoaromatic units are then cleaved by β-etherases belonging to the glutathione-S-transferase (GST) superfamily. In the last step, glutathione is removed from the intermediate compounds by GSTs with β-glutathione thioetherase (glutathione lyase) activity. To date, the β-glutathione thioetherase from strain SYK-6 is the only enzyme in this pathway to be biochemically characterized. However, a homology-based amino-acid database search for β-etherases coupled with biochemical characterization of the candidate enzymes identified several other β-etherases from the genomes of two *Novospingobium* species^23^,^24^ that were originally isolated from sea water^25^ and deep-subsurface sediment near the coast^26^.

To gain deeper insight into the involvment of microbes to lignin degradation in marine environments, we previously conducted functional screening of microbes isolated from deep-sea sediments and highly decomposed submerged wood, and identified a number of bacterial strains capable of degrading lignin-derived aromatic monomeric compounds^27^. Here, we screened this collection of marine isolates for microbes with β*-O-*4 ether linkage cleavage activity and mined the genes required for the reaction from the genomic sequence^28^ of one identified strain. Furthermore, gene expression profiles during exposure to two lignin model compounds were examined to elucidate the physiological changes that occur when the strain encounters lignin-related compounds.

## Results and Discussion

### Identification of a marine microorganism capable of cleaving the β-*O*-4 linkage of a dimeric lignin model compound

**Figure 1:**
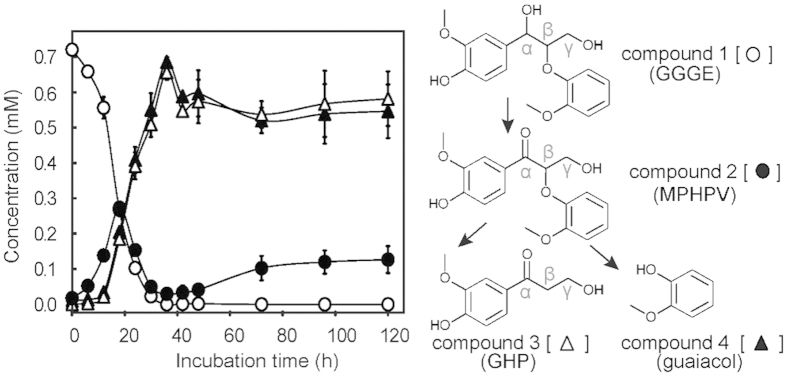
GGGE metabolism of strain MBES04. The decrease of GGGE and production of downstream metabolites were monitored by HPLC quantification performed in duplicate. After inoculation of strain MBES04 into basal medium supplemented with 0.9 mM GGGE (time 0 h), periodical sampling was conducted for 5 days from two independent cultures. The results of parallel experiments conducted with uninoculated medium confirmed that no significant abiotic change of GGGE or production of MPHPV, GHP, and guaiacol occurred. Compounds 1 (open circles), 2 (closed circles), 3 (open triangles), and 4 (closed triangles) denote GGGE, MPHPV, GHP, and guaiacol, respectively. Error bars indicate standard deviation.

We previously isolated several deep-sea bacteria capable of metabolizing lignin-derived aromatic monomers^27^. Here, we screened the isolates for strains capable of cleaving the β*-O-*4 ether linkage of the dimeric lignin model compound GGGE (Fig. 1, compound 1) and identified an isolate from sunken wood, *Novosphingobium* sp. strain MBES04, which metabolized GGGE into two end-products, guaiacylhydroxylpropanone (GHP; Fig. 1, compound 3) and guaiacol (Fig. 1, compound 4). GGGE metabolism by strain MBES04 was quantitatively examined during a 5-day culture in basal medium. The detection of a transient intermediate metabolite (Fig. 1, compound 2) indicated that GGGE was oxidized to MPHPV prior to cleavage of the β*-O-*4 ether linkage. GHP and guaiacol were produced as end products from MPHPV and accumulated in the culture medium at more than 60% of the estimated maximum yield.

### Metabolism of lignin-related aromatic monomers, dimers, and a crude extract from milled wood by strain MBES04

Strain MBES04 grew on a wide range of aromatic monomers and esters, including synaptic acid, ferulic acid, caffeic acid, 4-hydroxybenzoic acid, syringic acid, vanillic acid, vanillin, benzoate, protocatechuic acid, and chlorogenic acid, as well as hexoses and pentoses that are commonly distributed in terrestrial plants. Notably, however, strain MBES04 was not capable of growth using either GGGE or MPHPV as the sole carbon source in a minimal salt medium. To examine the metabolism of natural wood components by strain MBES04, a water-soluble fraction of dioxan extract (designated herein as WDM) of milled wood, *Quercus myrsinifolia*, was added to cultures of strain MBES04. After a 48-h incubation, metabolites in the culture supernatant were identified by reversed-phase LC/MS (Figure S1), which revealed that GHP and 3-hydroxy-1-(4-hydroxy-3,5-dimethoxyphenyl)-1-propanone (syringyl hydroxyl propanone; SHP), a methoxylated derivative of GHP, were the two predominant metabolites among a number of unidentified compounds. This finding is consistent with a study by Lancefield *et al.*^29^, who reported that GHP and SHP were specifically produced by the chemoselective breakdown of lignin model dimers, lignin-like synthetic polymers and milled wood lignin by the oxidation of Cα hydroxyl moieties followed by the reductive cleavage of β-*O*-4 ether linkages.

To assess the capability of strain MBES04 to depolymerize and/or modify natural lignin outside of cells, the extracellular activities of oxidative enzymes, including oxidases and peroxidases, which are known to function as bacterial lignin-modifying enzymes^30^, were measured in WDM-supplemented cultures once daily for 3 days using 2,2'-azino-bis(3-ethylbenzothiazoline-6-sulphonic acid) (ABTS) and 2,6-dimethoxyphenol as substrates with/without a divalent metal salt (FeSO_4_, CuSO_4_, and MnSO_4_) and with/without H_2_O_2_. However, the oxidative enzymatic activities in WDM-supplemented cultures were below the detection limit of the assay. This result suggests that oxidative enzymes have limited involvement in the depolymerization and modification of polymeric lignin outside of cells, even though the strain MBES04 genome contains putative genes encoding four catalase-peroxidases (GAM03180, GAM05894, GAM05893, and GAM04190) and two multicopper oxidases (laccases) (GAM04037 and GAM03576) (Table S1). Based on these findings, strain MBES04 appears to cleave the β-*O*-4 ether linkages present in partially depolymerized, low-molecular-weight lignin using intracellular enzymes.

### Identification of the genes involved in GGGE metabolism

**Figure 2:**
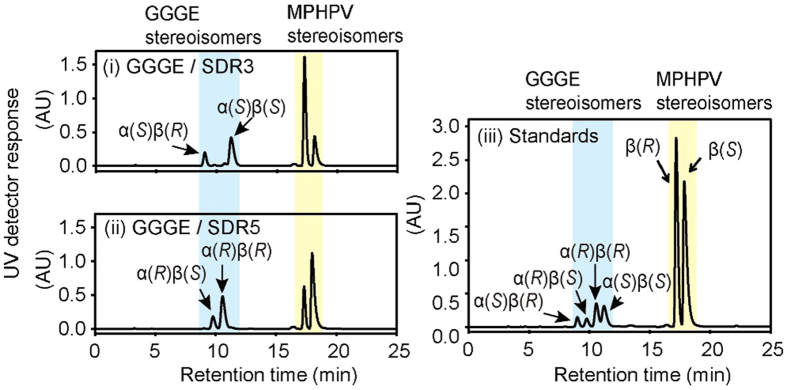
Chiral HPLC chromatograms of the enzymatic Cα dehydrogenation of GGGE to produce MPHPV. Four stereoisomers of GGGE were mixed with nicotinamide adenine dinucleotide sodium salt and either SDR3 or SDR5. After 16-h incubation, the reactions by SDR3 (**i**) and SDR5 (**ii**) were monitored by chiral HPLC analysis. The chromatogram (**iii**) shows synthetic GGGE and MPHPV as standards.

According to an earlier study of *Sphingobium* sp. strain SYK-6^20^, GGGE metabolism mediated by SDRs and GSTs produces the intermediates MPHPV, GHP, and guaiacol, which were also produced from GGGE by strain MBES04. Among the 58 genes in the strain MBES04 genome^28^ that showed similarity to reported SDRs of Sphingomonadaceae family members and encoded short-chain alcohol dehydrogenases, 6 candidate genes were selected based on similarities to the 4 SDR genes reported to function as Cα-dehydrogenases (accession numbers: NC_015976/Gene ID; BAK65539, BAK68041, BAK68265, and BAK68263) in strain SYK-6^20^,^21^ and expressed as His-tagged proteins in *E. coli* (Table S2). The recombinant SDRs were purified and assessed for their ability to dehydrogenate the Cα position of GGGE. Only two recombinant SDRs (SDR3 and SDR5; Figure S2a), encoded by the genes GAM05523 and GAM05547, respectively, exhibited dehydrogenase activity in the presence of nicotinamide adenine dinucleotide (NAD). SDR3 selectively acted on α(*R*)-substrate, whereas SDR5 was selective for α(*S*)-substrate (Fig. 2).

**Figure 3:**
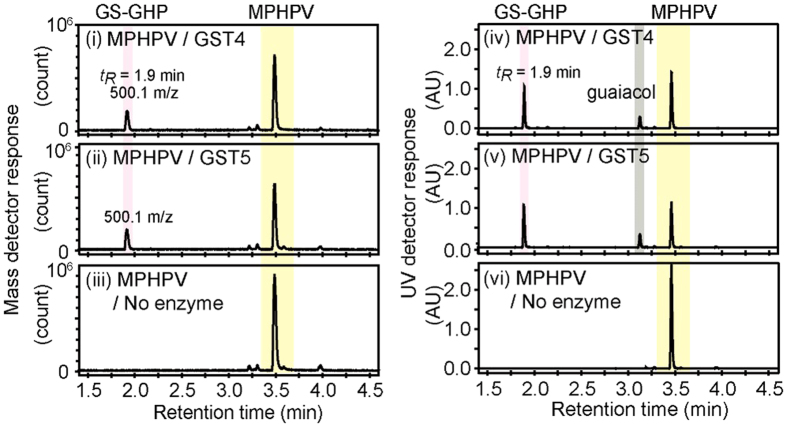
LCMS analysis of the β-*O*-4 cleavage reactions of MPHPV by GST4 and GST5. Two stereoisomers of MPHPV were incubated with glutathione and either GST4 (**i**,**iv**) or GST5 (**ii**,**v**) for 16 h. Reaction mixtures without added enzymes were used as controls (**iii**,**vi**). After incubation, the reaction products were analyzed by LCMS. The glutathione adduct of GHP (GS-GHP, *t*_*R*_ = 1.9 min) has a molecular mass of 500.1 m/z. Guaiacol was identified on UV detection (right), but was not detected on the mass chromatogram (left) due to evaporation caused by the carrier gas at 500 °C in the mass detector.

**Figure 4:**
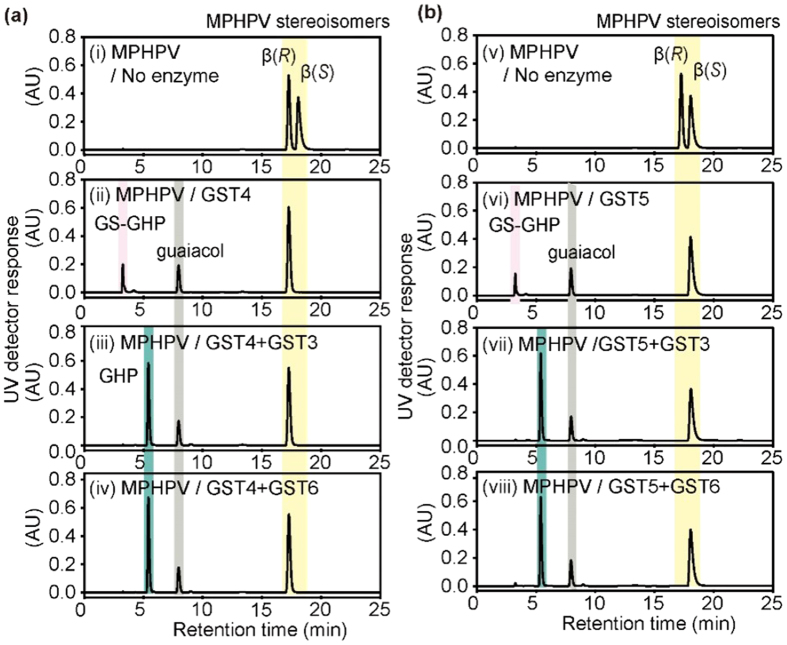
Stereo-specificity of the enzymatic cleavage of the β-*O*-4 linkage of MPHPV and removal of glutathione from the glutathione adduct of GHP (GS-GHP). **(a)** Two stereoisomers of MPHPV were mixed with glutathione and the resulting mixture (i) was incubated with GST4 (ii). Either GST3 (iii) or GST6 (iv) was then added to the reaction mixture to remove the glutathione from GS-GHP produced by the catalytic activity of GST4. **(b)** Two stereoisomers of MPHPV were mixed with glutathione (v) and incubated with GST5 (vi). Either GST3 (vii) or GST6 (viii) was then added to the reaction mixture to remove the glutathione from GS-GHP produced by the catalytic activity of GST5.

A total of 18 genes in the strain MBES04 genome were predicted to encode GST family proteins. BLASTP analyses with sequences of reported β-etherases (BAK65541, BAK65540, and BAK67935) and a glutathione lyase (BAK65542) detected three putative GST genes that may be involved in GGGE metabolism. The three identified GST genes (GST4–6; GAM05530, GAM05531, and GAM05532) were grouped in a cluster in the same orientation. In addition, a fourth putative GST gene (GST3; GAM05529) was found upstream of the three GST genes, but in the opposite orientation. These four GST genes were expressed in *E. coli* as His-tagged proteins (Table S2), which were then purified (Figure S2b) and assessed for enzymatic activity. Two recombinant GSTs (GST4 and GST5) catalyzed the cleavage of β*-O-*4 ether linkages in MPHPV using glutathione as a cofactor to produce glutathione conjugates of GHP (Fig. 3). GST4 selectively eliminated the ether-linked moiety of the β(*S*)-enantiomer (Fig. 4a), whereas GST5 reacted exclusively with the β (*R*)-enantiomer (Fig. 4b). Neither GST4 nor GST5 cleaved the β*-O-*4 ether linkages in GGGE. GST3 and GST6 did not catalyze cleavage of the β*-O-*4 ether linkages both in GGGE and MPHPV but the removal of glutathione from both glutathione conjugates of GHP produced by GST4 and GST5 under these reaction conditions (Fig. 4a,b). Based on these results, it was determined that GST4/GST5 and GST3/GST6 function as β-etherases and β-thioetherases, respectively. Notably, GST6 showed markedly lower activity toward the glutathione conjugate produced by GST5 when present at low enzyme concentrations, indicating that the two glutathione conjugates produced by GST4/GST5 have different configurations.

Recently, Gall *et al.*^31^ determined the configuration of glutathione-conjugated intermediates and demonstrated that all of the enzymatic reactions were strictly stereospecific. To date, no enzyme capable of reacting efficiently with the β(*S*)-glutathione conjugate produced by the enzymes LigE and LigP, which are encoded by BAK65541 and BAK67935, respectively, has been identified. Therefore, it has been suggested that a racemase-like or other enzyme with different stereospecificity from LigG (a β(*S*)-stereospecific β-thioetherase, BAK65542) for glutathione removal, or other metabolic pathway for the cellular utilization of glutathione conjugates, are functional in strain SYK-6 cells^31^. In the present study, GST3 displayed no apparent preference for either epimer of the glutathione conjugate substrate. Thus, this is the first report of an enzyme capable of directly removing glutathione from a glutathione conjugate in both the β(*R*) and β(*S*) configurations.

Based on structural modeling deduced from amino acid sequences of GST3/GST6 using the Swiss model workspace^32^, GST6 is predicted to be a member of the GST omega class, as expected, whereas GST3 is proposed to be a member of the *Nu*-class. YghU and YfcG from *E. coli*^33^ and Ure2p from the wood-degrading fungus *Phanerochaete chrysosporium*^34^ are partially characterized members of the *Nu*-class within the GST family. YghU and YfcG exhibit distinct disulfide bond oxide-reductase activities (scheme 1) and little or no GSH transferase activity towards typical electrophilic substrates^33^,^35^,^36^. *Nu*-class GSTs are atypical in that they bind two molecules of GSH in each active site. Ure2p from *P. chrysosporium* is able to efficiently deglutathionylate GS-phenacylacetophenone and interacts *in vitro* with an omega class GST. The present finding that a novel GST belonging to the *Nu*-class catalyzes the reductive removal of glutathione from glutathione conjugates (scheme 2) and has no preference for the glutathione adducts produced by β(*S*) and β(*R*) specific-etherases provides new insight into microbial lignin metabolism. The protein structure of the GST enzyme will be solved in future work.

scheme 1: E*2GSH + R-S-S-R ↔ E*GSSG + 2RSH

scheme 2: E*GSH + R-SG ↔ E*GSSG + RH

**Figure 5:**
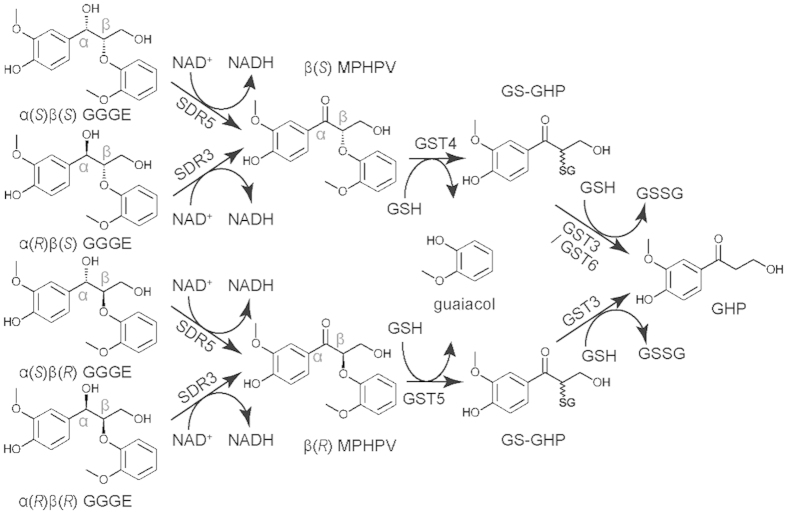
Possible pathway of GGGE metabolism in strain MBES04. The responsible enzymes and their required cofactors are shown. Abbrevations: GS-GHP, glutathione adduct of GHP; NAD^ + ^, oxidized form of nicotinamide adenine dinucleotide (NAD); NADH, reduced form of NAD; GSH, reduced form of glutathione; and GSSG, oxidized form of glutathione.

A possible pathway of GGGE metabolism in strain MBES04, including the responsible enzymes and required cofactors, is presented in Fig. 5. However, this proposed pathway remains speculative and requires corroboration by gene disruption experiments.

### Biochemical characterization of SDRs and GSTs

To compare the catalytic properties of the SDRs and GSTs identified in this study to closely related enzymes showing the same catalytic activity, the purified SDR and GST enzymes from strain MBES04 were biochemically characterized. The pH and temperature optima for the SDR (SDR3 and SDR5) and GST (GST4 and GST5) activities were determined using GGGE and MPHPV, respectively, as substrates (Table 1). The optimal temperatures for GST4 and GST5 activities were higher than the reported β-etheraes (Figure S3b). The specific activities and kinetic parameters of SDR3 and SDR5 were also measured under the optimal reaction conditions using GGGE and veratryllglycerol-β-guaiacyl ether (VGGE), which is a non-phenolic derivative of GGGE, as substrates (Table 1). The catalytic efficiency (specificity constant) (*k*_cat_/*K*_m_) of SDR3 and SDR5 for GGGE were 1.6 × 10^1^ and 8.6 × 10^3^ min^−1^mM^−1^, respectively, values that are one order of magnitude lower and higher, respectively, than that of LigD of strain SYK-6^37^. The specific activities and kinetic parameters of GST4 and GST5 were determined using MPHPV and β-guaiacyl-α-veratrylglycerone (GVG) as substrates (Table 1, Table S3). GVG is a non-phenolic derivative of MPHPV and has been used for determination of the specific activities of β-etherases^24^. The specific activities of GST4 and GST5 for GVG were 3.7 × 10^2^ and 1.7 × 10^2 ^mU/mg, respectively. These specific activities were within the range of those reported in a previous study, in which the specific activities ranged from 1.0 × 10^2^ to 6.8 × 10^3^ mU/mg for the reported enzymes encoded by the members of sphingomonads^24^ (Table S3).

The GST activities of GST3, GST4, GST5, and GST6 were also screened using commercially available nucleophilic substrates that are widely used in conventional GST assays^35^,^36^. However, no GST activity towards any of the tested compounds was detected, with the exception of the activity of GST3 towards 1-chloro-2,4-dinitrobenzene (CDNB) (675.0 ± 16.3 mU/mg). These results indicate that the substrate specificity of GST3 is broader than that of GST4–6.

### Distribution of SDR and GST homologs involved in GGGE metabolism

BLASTP searches using the deduced amino acid sequences of the two SDR and four GST genes identified in strain MBES04 as queries against the NCBI nr protein database, which covers non-redundant GenBank CDS translations, RefSeq, PDB, SwissProt, PIR, PRF, excluding those in PAT, TSA, and env_nr (http://www.ncbi.nlm.nih.gov/), was performed to find homologs reported to have β-etherase or β-thioetherase activity^20^,^23^,^24^. A phylogenetic tree was constructed from alignments of the 15 most similar amino acid sequences to SDR3, SDR5, GST3, GST4, GST5, and GST6 (Figure S4). GST4 and GST5 clustered together with LigEs and LigFs, respectively, which are known to function as β(*R*)- and β(*S*)-stereospecific etherases, respectively. GST6 was classified together with LigG, a β(*S*)-stereospecific β-thioetherase belonging to the omega-class of the GST family. The enzymes characterized in the present and previous studies (highlighted by boxes in Figure S4) that belonged to the same clade, also shared the same substrate specificities. In contrast, GST3 was assigned to an uncharacterized branch of GST family proteins, indicating that this enzyme belongs to a new class within the GST family that targets lignin model dimers.

BLASTP searches using the deduced amino acid sequences of the four GST genes identified in strain MBES04 as queries showed that the putative GST proteins with homology to GST3 or GST6 were widely distributed among members of the α-, β-, and γ-proteobacteria classes. However, only a small number of homologous proteins to GST4 and GST5 were found. Proteins with similarity to GST4 (E value < e-50) were found exclusively in the Sphingomonadaceae family and were annotated as LigF/GST proteins, whereas proteins with homology to GST5 were found among members of α- and δ-proteobacteria and were predominantly annotated as β-1,3-glucanases.

Among members of the Sphingomonadaceae family, 15 complete and 69 draft genome sequences are currently available. Here, the distribution of GGGE metabolic genes in this family was investigated using BLASTP and BLASTX searches against predicted coding sequences and nucleotide sequences, respectively (Table S4). At least one complete set of GGGE-converting enzymes, consisting of Cα-dehydrogenases, β-etherases, and β-thioetherases, was found to be essential for GGGE metabolism in this family. A total of 9 strains were identified as candidates with GGGE metabolic activity and included 7 strains that were isolated from water-logged environments, such as rivers, lakes, sludge, subsurface sediment/water, and seawater, whereas the other 2 strains were obtained from a decomposing plant and the rhizosphere.

**Figure 6:**
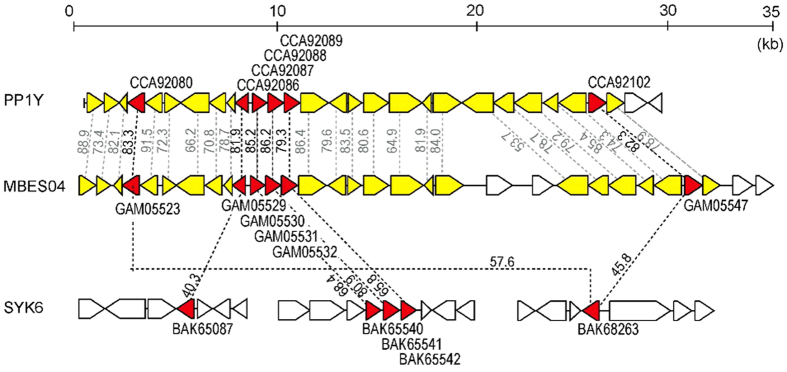
Arrangement of the GGGE-metabolizing genes and neighboring loci in strain MBES04 in comparison with selected species possessing homologs of these genes. The red arrows show commonly conserved genes among strain MBES04, *Novosphingobium* sp. PP1Y and *Sphingobium* sp. SYK-6. The white arrows indicate genes that have no mutual relationship among the three strains. The yellow arrows show conserved genes among strains MBES04 and PP1Y. Each number indicates the similarity value (%) at the amino acid sequence level.

The organization of the GGGE metabolizing-gene homologs was investigated in the 9 identified Sphingomonadaceae strains (Table S5). The tandem arrangement of SDR3 and SDR5 homologs was detected in 6 strains, and multiple GSTs were found in neighboring loci in 4 strains. Two strains, SYK-6 and a marine isolate, *Novosphingobium* sp. PP1Y, possessed a complete set of GGGE metabolizing-gene homologs encoding enzymes capable of cleaving the β*-O-*4 linkage of lignin model dimers, which were comprised of four stereoisomers with two chiral carbon centers. As was observed in strain MBES04, the four GGGE-metabolizing GST genes were clustered together in strain PP1Y, which was also found to share gene synteny with strain MBES04 (Fig. 6).

The tight clustering of all four GGGE-metabolizing GST genes in the two marine isolates, strain MBES04 and PP1Y, suggests that these genes may confer an evolutionary advantage in the marine environment and/or be coordinately regulated and expressed. To elucidate the origin, evolution and diversity of the genes involved in β*-O-*4 reductive cleavage and gain a better understanding of the evolutionary processes controlling the assembly of the corresponding enzymes involved in lignin metabolism, pan-genomic studies and experimental evidence at the protein level are needed.

### Effect of lignin model dimers on global gene expression

qPCR quantification of GGGE-metabolizing gene expression, which ranged from 10^−5^ to 10^−6^ fold of that of the 16 S rRNA gene, confirmed the low level expression of these genes, and no apparent differences between the control and GGGE-added conditions were detected (Figure S5). Whole-genome transcriptional profiling in the early exponential phase was conducted to detect the response of strain MBES04 to the lignin-related compounds GGGE and MPHPV (Table 2, Table S6, Figure S8). A total of 28 and 51 genes were upregulated in response to medium supplemented with GGGE and MPHPV, respectively, and included 12 common genes between these two groups. In particular, the expression of genes involved in glycerol metabolism for biosynthesis of the cellular membrane was clearly increased. The gene expression analysis also revealed that 5 and 15 genes were down-regulated in strain MBES04 in response to GGGE and MPHPV, respectively. In response to GGGE alone, expression of genes involved in energy metabolism, including aromatic monomers such as toluene and benzoate, and fatty acid degradation were enhanced. The upregulation of the energy metabolism gene expression may promote the growth of strain MBES04 on lignin-derived aromatic compounds immediately upon exposure to plant materials, including lignin-derived aromatic compounds. In addition, the elevated expression of several stress-response genes, such as the transcriptional regulators PadR and CopG, and multidrug transporters was observed. PadR and CopG are involved in stress responses to various aromatic compounds^38^,^39^ and plasmid replication^40^. Elevated expression of multiple drug transporters is indicative of enhanced stress responses against diverse antimicrobial agents^41^. These responses of strain MBES04 are consistent with the coordinated regulation of the microbial gene expression associated with lignin transformation, as predicted from the analysis of lignin-transforming bacterial scaffolds^16^. In cells exposed to MPHPV, increased expression of a greater number of stress-response genes and a few genes involved in energy metabolism were detected compared to GGGE-supplemented conditions. Specifically, in response to MPHPV, the gene encoding the flagellar basal body-associated protein involved in chemotaxis^42^ was enhanced, and several cytochrome C proteins involved in the respiratory chain for energy production were repressed. Energy production may be reduced by repression of cytochrome proteins involved in the respiratory chain, a response that has been shown to lead to cellular dormancy^43^. Such dormancy is referred to as a “bed-hedging” strategy, and is employed by many microorganisms to sustain viability in unfavorable environmental conditions^44^. Thus, the response of strain MBES04 to the lignin model dimers MPHPV and GGGE may be a survival strategy for utilizing the abundant TerrOC discharged into the ocean. The findings from this study provide insight into previously unidentified bacterial enzymatic systems and the physiological acclimation of microbes associated with the biological transformation of TerrOC containing lignin in marine environments.

## Methods

For more detailed descriptions of the materials and methods used in this study, please refer to the Supplementary text.

### Synthesis of β-ether-linked model lignin dimers and associated metabolites

1-(4-Hydroxy-3-methoxyphenyl)-2-(2-methoxyphenoxy)-1,3-propanediol (guaiacylglycerol-β-guaiacyl ether; GGGE) and 3-hydroxy-1-(4-hydroxy-3-methoxyphenyl)-2-(2-methoxyphenoxy)-1-propanone ((2-methoxyphenoxy)hydroxy-propiovanillone; MPHPV) were synthesized according to the method of Hosoya *et al.*^45^. 1-(3,4-Dimethoxyphenyl)-2-(2-methoxyphenoxy)-1,3-propanediol (veratrylglycerol-β-guaiacyl ether; VGGE) and 3-hydroxy-1-(3,4-dimethoxyphenyl)-2-(2-methoxyphenoxy)-1-propanone (β-guaiacyl-α-veratrylglycerone; GVG) were synthesized by a similar scheme as used for GGGE with minor modifications according to the description of Picart *et al.*^24^. The synthesized GGGE and VGGE were characterized and assigned using liquid chromatography/mass spectroscopy (LC/MS) and ^13^C-NMR. LC/MS data were generated using a Waters Xevo G2 quadrupole time-of-flight mass spectrometer operated in negative ion ESI mode. The inlet system was a Waters Acquity H-class UPLC system and was operated at a flow rate of 0.4 mL/min using a BEH C18 reverse phase column (1.8-μm particle size, 100 × 2.1 mm; Waters) using the mobile phase gradients A (2 mM sodium acetate and 0.05% formic acid) and B (95% acetonitrile/H_2_O) and the following conditions: from 0– 6 min, 95%-5% A with B as the remainder; and from 6–7 min, 100% B. The eluate was monitored at 270 nm using a Waters photodiode array (PDA) eλ detector. The measured mono-isotopic masses (M-H^ + ^GGGE/C_17_H_19_O_6_; 319.1, VGGE/C_18_H_21_O_6_; 333.1) agreed with those calculated from the respective molecular formulas. The ^13^C-NMR spectra of synthetic GGGE (Figure S7) and VGGE (Figure S8) matched those deposited in the NMR Database of Lignin and Cell Wall Model Compounds (http://ars.usda.gov/SP2UserFiles/Place/36553000/software/NMR/NMR_DB_11-2004.pdf).^46^ and the spectra reported by Picart *et al.*^24^. The ratios of stereoisomers in synthetic GGGE and MPHPV were determined by chiral chromatography based on the peak areas of the isomers. GGGE contained α(*S*)β(*R*) GGGE, α(*R*)β(*S*) GGGE, α(*S*)β(*S*) GGGE, and α(*R*)β(*R*) GGGE at a ratio of 1:1:3:3. MPHPV contained β(*R*) MPHPV and β(*S*) MPHPV at a ratio of 1:1 (Figure S9).

For the structural analysis of the unidentified metabolite from GGGE, 3-hydroxy-1-(4-hydroxy-3-methoxyphenyl)-1-propanone (guaiacylhydroxylpropanone; GHP) was chemically synthesized via an aldol reaction of acetovanillone and formalin. GHP was characterized using 2D COSY, HSQC, and HMBC (Figure S10a,b).

### Strains and media

*Novosphingobium* sp. strain MBES04 (NITE AP-01797) was grown aerobically with shaking at 30 °C in basal medium consisting of Luria-Bertani (LB) medium supplemented with 5 mM MgSO_4_. For testing carbon utilization, a defined mineral medium containing 1 mM of the test substrate as the sole carbon source was used. The mineral medium (100 mL) consisted of 20 mL basal salt solution (33.9 g Na_2_HPO_4_, 15.0 g KH_2_PO_4_, 10.0 g NaCl, and 5.0 g NH_4_Cl per liter of deionized H_2_O), 0.5 mL of 1 M MgSO_4_, 1 mL of 0.25% (w/v) Daigo’s IMK medium (Wako), 1 mL trace vitamins solution, 1 mL of 100 mM substrate stock solutions, and 86.5 mL deionized H_2_O. The trace vitamin solution was prepared according to Balch *et al.*^47^. Prior to use, the medium was sterilized using a 0.22-μm membrane filter. Substrate stock solutions of 100 mM GGGE, MPHPV, synaptic acid, ferulic acid, caffeic acid, 4-hydroxybenzoic acid, syringic acid, vanillic acid, vanillin, protocatechuic acid, and chlorogenic acid were prepared using N,N-dimethylformamide (DMF) as a solvent. Stock solutions of 100 mM sodium benzoate, arabinose, and xylose were prepared in deionized H_2_O. Mineral medium containing 1 mM glucose with/without 1% (v/v) DMF was used as a positive control for growth. The growth of strain MBES04 was not affected by supplementation of the medium with 1% DMF.

### Metabolism of a crude extract from milled wood

*Quercus myrsinifolia* sawdust was milled at 25,000 rpm for 2 min using a Wander blender (D3V-10, Osaka Chemical, Osaka, Japan). The coarse grain was removed by passing the material through a 0.1-mm mesh sieve. A total of 10 g milled wood was immersed in 1 L dioxan-water (96:4) for 2 days at room temperature. The extract was recovered by filtration and dried under vacuum to obtain a crude lignin-rich material, which was suspended in water at 0.4% (w/v) and then autoclaved at 120 °C for 15 min. The suspension was filtered through a 0.22-μm membrane to obtain the water-soluble fraction, which was designated as WDM (water-soluble fraction of dioxan extract from milled wood). A quarter volume of WDM was added to basal medium as a low-molecular-weight lignin containing crude natural materials. Strain MBES04 was cultured using 10 mL WDM-supplemented medium in triplicate. After 48-h cultivation, the culture broth was centrifuged at 10,500 × *g* for 10 min to remove all cells and debris, and the obtained supernatant was analyzed by LC/MS. Control experiments were performed in triplicate using basal medium containing WDM without inoculation of strain MBES04 (control 1) and using basal medium without WDM, but with inoculation of strain MBES04 (control 1). All LC/MS loading data were analyzed with multivariate statistics using MarkerLynks XS software (Waters). An OPLS-discriminant model was constructed and visualized in an S-plot to detect differences between the data obtained from the WDM-supplemented culture medium and those from the control experiments. Ten MS ions with high loadings (>0.05) and correlations (>0.9) were selected as potential metabolites from WDM and were used for quantification based on the peak area in the MS chromatograms. Metabolites were identified by comparing the retention times (*t*_*R*_) and MS spectral patterns with those of GHP and SHP standards. Authentic SHP was purchased from Tokyo Fine Chemicals (Tokyo, Japan).

### Assessment of oxidase and peroxidase activities of strain MBES04

The supernatant of 48-h cultures of MBES04 grown in WDM-supplemented medium was used for the assessment of oxidase and peroxidase activities of the strain. Oxidase activity was assayed every 24 h for 3 days according to a method described in the literature^48^ with minor modifications. Briefly, 0.5 mM 2,2'-azino-bis(3-ethylbenzothiazoline-6-sulphonic acid) (ABTS) and 1 mM 2,6-dimethoxyphenol (DMP) were used as substrates in reaction mixtures with and without 0.5 mM each of the divalent metal salts of FeSO_4_, CuSO_4_, and MnSO_4_. After adding 40 μL of culture supernatants to the assay mixtures to make a total volume of 200 μL, increases in absorbance at 420 and 480 nm for the ABTS and DMP assays, respectively, were monitored every hour for 4 h with a Powerscan HT microplate reader (Dainippon Pharmaceutical) at 25 °C. Peroxidase activity was assayed in the presence of 0.1 mM H_2_O_2_ using the same substrates and metal ions used for the oxidase assays. Uninoculated medium incubated under the same condition as the test cultures was used as a control for abiotic-induced changes in the absorbance.

### Preparation of expression plasmids and enzyme purification

The whole-genome shotgun sequence of strain MBES04 was previously determined by our group^28^. A total of 124 contigs were deposited at DDBJ/EMBL/GenBank under the accession numbers BBNP01000001 to BBNP01000124. Candidate GGGE-metabolizing genes of strain MBES04 were identified by querying all detected ORFs in the MBES04 draft genome with known GGGE-metabolizing genes of *Sphingobium* sp. SYK-6 (accession numbers NC_015976/ Gene ID; BAK65539, BAK65541, BAK65540, BAK65542, BAK68041, BAK68265, BAK68263, and BAK67935) using BLASTP. DNA fragments containing possible genes encoding GGGE-metabolizing enzymes were amplified by PCR using the primer sets listed in Table S2 and were then cloned into the pRSET A expression vector (Life Technologies, Carlsbad, CA, USA), which was used to add a His × 6 tag at the N-terminus of the target protein. Enzyme purification from transformant *E. coli* strain BL21(DE3)pLysE cells was conducted using cOmplete His-tag purification resin (Roche Diagnostics, Basel, Switzerland). The purity of protein preparations was confirmed by SDS-15% PAGE (Figure S2a,b).

### Analysis of GGGE metabolism

Strain MBES04 was cultured in basal medium containing 0.9 mM GGGE at 30 °C with shaking for 5 days. The culture supernatants were sampled every 6 h for 2 days, followed by every 24 h until the 5th day, and were analyzed by reversed-phase HPLC using a Waters Alliance 2796 Liquid Chromatography (LC) system (Waters) equipped with an Xbridge C18 reversed-phase column (3.5-μm particle size, 100 × 4.6 mm; Waters) operated at a flow rate of 1.2 mL/min using the mobile phase gradients A (2 mM sodium acetate and 0.05% formic acid) and C (95% methanol/H_2_O) under the following two conditions: 0–1 min, 90% A and 10% C, 1–8 min, a decreasing gradient of 90%–10% A with C as the remainder; and 8–10 min, 100% C. The eluate was monitored at 270 nm using a Waters 2998 PDA detector. The amount of substrate and metabolites in the culture supernatant was calculated based on the area of the corresponding chromatographic peaks. Uninoculated medium incubated under the same conditions as the test cultures was used as a blank sample to assess the effect of the abiotic degradation of GGGE. The procedure used for the structural determination of the metabolites is described in the Supplementary text (Figures S10 and S11).

### Preparation and structural characterization of metabolites produced by strain MBES04

Medium composed of 6 g Daigo artificial seawater (Wako), 0.9 g Difco tryptone peptone, 0.9 g Bacto yeast extract, 288 mg GGGE, and 300 mL tap water was inoculated with strain MBES04 and was then incubated at 30 °C for 150 h with shaking at 120 rpm. Metabolites in the medium were then extracted with ethyl acetate and purified by silica gel (Wakogel C-200) column chromatography, yielding a total of 140 mg crystals. The purified metabolites recovered from the culture supernatant and chemically synthesized GHP were analyzed by LC/MS as described above, ^1^H-NMR at 500 MHz in CDCl_3_ and ^13^C-NMR at 126 MHz in CDCl_3_ (Figure S11a,b).

### SDRs and GSTs reactions of lignin model dimers

The enzymatic conversions of four mixed stereoisomers of GGGE (1.0 mM) were performed with the recombinant enzymes SDR3 (encoded by GAM05523, 10.0 μg/mL) or SDR5 (encoded by GAM05547, 5.0 μg/mL) with NAD sodium salt (2.0 mM) as a cofactor for 16 h at 15 or 25 °C, respectively. The enzymatic conversion of two mixed stereoisomers of MPHPV (1.0 mM) was conducted with one or two enzymes selected from GST3 (encoded by GAM05529, 5.0 μg/mL), GST4 (encoded by GAM05529, 5.0 μg/mL GST5 (encoded by GAM05531, 50.0 μg/mL), and GST6 (encoded by GAM05532, 5.0 μg/mL) using glutathione (2.0 mM) as a cosubstrate for 16 h at 25 °C.

### Biochemical and kinetic characterization of SDRs and SGRs

SDR3 and SDR5 were characterized using 10 mM GGGE as a substrate and 20 mM NAD sodium salt as a cofactor. The formation of the reaction product, MPHPV, after 30-min incubation was determined by HPLC as described above. GST4 and GST5 were characterized using 5 mM MPHPV as a substrate and 10 mM GSH as a cofactor. The formation of the reaction product, guaiacol, was measured by HPLC. The determination of the pH optimum for enzymatic activity was performed using the following buffers (100 mM): 2-(N-morpholino) ethanesulfonic acid (pH 5.5 to 7.0), 3-morpholinopropanesulfonic acid (pH 7.0 to 8.0), N-Tris(hydroxymethyl)methyl–3-aminopropanesulfonic acid (pH 8.0 to 9.0), N-cyclohexyl-2-aminoethanesulfonic acid (pH 9.0 to 10.0), and N-cyclohexyl-3-aminopropanesulfonic acid (pH 10.0 to 11.0). The optimal temperature was determined by measuring the formation of each reaction product after 30-min incubation at the optimal pH for each enzyme at temperature ranges of 5–45 °C for SDR3 and SDR5, and 15–45 °C for GST4 and GST5. The experiments were performed in triplicate.

Kinetic measurements were conducted for 30 min with the substrates (final concentrations) GGGE and VGGE (0.06 to 5.0 mM), MPHPV (0.06 to 2.5 mM), and GVG (0.06 to 1.5 mM). The highest concentration of each substrate was determined according to the maximum solubility of each compound in the tested reaction mixture. The formation of MPHPV from GGGE by SDR3/SDR5, GVG from VGGE by SDR3/SDR5, and guaiacol from MPHPV and GVG by GST3/GST5 was measured by HPLC. The kinetic experiments were performed in triplicate. The *K*_m_ and *v*_max_ values were calculated from a hyperbolic regression analysis using Hyper32 software (version 1.0.0.; http://homepage.ntlworld.com/john.easterby).

GST activities (10 μg GST3, GST4, GST5, and GST6) toward the commercially available nucleophilic substrates phenethyl isothiocyanate, CDNB, and 4-nitrophenyl butyrate were assessed according the method described by Mathieu *et al.*^36^. 1,2-Dichloro-4-dinitrobenzene, ethacrynic acid, and 4-nitrobenzyl chloride were also used as substrates^37^.

### Chiral chromatography

The enzymatic reaction mixtures prepared above were pretreated by solid-phase extraction and were then injected into a CHIRALPAK IE-3 column (Daicel Chemical Industries) for separation of the stereoisomers α(*S*)β(*R*) GGGE, α(*R*)β(*S*) GGGE, α(*S*)β(*S*) GGGE, α(*R*)β(*R*) GGGE, β(*R*)MPHPV, and β(*S*) MPHPV. A mixture of acetonitrile and H_2_O was used as the mobile phase at a flow rate of 1.0 mL/min. The acetonitrile concentration of the mobile phase was adjusted as follows (the remainder was H_2_O): 0–10 min, 20% acetonitrile; 10–15 min, gradient from 20% to 30% acetonitrile; and 15–30 min, 30% acetonitrile. The absorbance of the eluate was monitored at 270 nm using a Waters 2998 PDA detector. The *t*_*R*_ values of the GGGE and MPHPV isomers are shown in Figure S9. Peak identification was based on optical rotation, as described by Hishiyama *et al.*^49^.

### RNA isolation and purification

Strain MBES04 was grown aerobically overnight with shaking at 30 °C in basal medium and was then subcultured (1:100) in 0.1 L of basal medium supplemented with 1 mM GGGE or MPHPV at 30 °C for 6 h. Cells cultured in basal medium without GGGE and MPHPV were used as controls. Cells were collected by centrifugation at 10,500 × *g* for 5 min at 4 °C. RNA was isolated and purified from the pelleted cells using an RNeasy kit (Qiagen, Valencia, CA, USA) following the manufacturer’s manual. Total RNA was eluted in 100 μL RNase-free H_2_O, and DNase I digestion of genomic DNA was then performed on a column using RNase-free DNase I (Qiagen) according to the manufacturer’s protocol. The obtained sample was then subjected to a second RNeasy purification step. RNA quality in the purified solutions was verified by quantification of the A260/A280 and A260/A230 ratios using an e-Spect spectrophotometer (Malcom, Tokyo, Japan) and by electrophoresis on an Agilent Bioanalyzer to detect intact 16 S and 23 S rRNAs.

### Quantitative PCR (qPCR)

Total RNAs were reverse transcribed using the Transcriptor First Strand cDNA Synthesis Kit (Roche Diagnostics) and used in the subsequent qPCR reaction, which was performed with Light Cycler 480 SYBR Green Master Mix (Roche Diagnostics) in a Roche Light Cycler 480. The 16 S rRNA gene was used as a reference. The primers used for qPCR are listed in Table S7. All qPCR experiments were performed independently in duplicate.

### RNA sequencing and data analysis

RNA sequencing libraries were constructed using DNA– and rRNA–free RNA samples, and were then sequenced using an Illumina Hiseq 2000 platform at the Beijing Genome Institute (BGI, Shenzhen, China), as previously described^50^. The obtained reads were mapped to the strain MBES04 draft genome using the short-read aligner Bowtie (http://bowtie-bio.sourceforge.net)^51^. Differentially expressed genes (DEG) were identified by the methods described in the Bioconductor project^52^ and included iDEGES^53^ and edgeR analyses^54^. Statistical significance was defined as a P-value of <0.05 in a negative binomial test following correction for false discovery rate^55^. The pathways involved in the physiological response to lignin model dimers were inferred using the KEGG Automatic Annotation Server with manual curation^56^.

## Additional Information

**How to cite this article**: Ohta, Y. *et al.* Combination of six enzymes of a marine *Novosphingobium* converts the stereoisomers of β-*O*-4 lignin model dimers into the respective monomers. *Sci. Rep.* **5**, 15105; doi: 10.1038/srep15105 (2015).

## Supplementary Material

Supplementary Information

## Figures and Tables

**Table 1:** Biochemical and kinetic characterization of SDRs (a) and GSTs (b) from strain MBES04

a	Parameter	Substrate	Enzyme	
			SDR3	SDR5
	optimal pH	GGGE	9	9–10
	optimal Temperature (°C)		15	30
	specific activity (mU/mg)		3.4E+02	4.3E+04
	*v*_max_ (mU)		4.2E+02	4.6E+04
	*K*_M_ (mM)		9.7E−01	2.0E−01
	*k*_cat_ (min^−1^)		1.6E+01	1.7E+03
	*k*_cat_/*K*_M_ (min^−1^ mM^−1^)		1.6E+01	8.6E+03
	specific activity (mU/mg)	VGGE	7.4E+01	2.9E+04
	*v*_max_ (mU)		1.0E+02	3.8E+04
	*K*_M_ (mM)		1.5E+00	1.1E+00
	*k*_cat_ (min^−1^)		3.9E+00	1.4E+03
	*k*_cat_/*K*_M_ (min^−1^ mM^−1^)		2.5E+00	1.3E+03

b	Parameter	Substrate	Enzyme	
			GST4	GST5
	optimal pH	MPHPV	9	7−8
	optimal Temperature (°C)		35	40
	specific activity (mU/mg)		1.0E+03	8.2E+01
	*v*_max_ (mU)		1.3E+03	9.8E+01
	*K*_M_ (mM)		4.7E−01	2.9E−01
	*k*_cat_ (min^−1^)		4.5E+01	3.5E+00
	*k*_cat_/*K*_M_ (min^−1^ mM^−1^)		9.5E+01	1.2E+01
	specific activity (mU/mg)	GVG	3.7E+02	1.7E+02
	*v*_max_ (mU)		5.3E+02	2.7E+02
	*K*_M_ (mM)		5.5E-01	7.8E-01
	*k*_cat_ (min^−1^)		1.8E+01	9.5E+00
	*k*_cat_/*K*_M_ (min^−1^ mM^−1^)		3.2E+01	1.2E+01

**Table 2:** Upregulated genes with putative functions in strain MBES04 in response to GGGE.

Gene ID	Putative function	Foldchange	KOentry*	KEGG pathway orDefinition**
GAM03580	cation efflux protein	3.6		−***
GAM03456	*p*-cresol methylhydroxylase subunit	2.9	K05797	toluene degradation
GAM03457	*p*-cresol methylhydroxylase subunit	2.6		toluene degradation
GAM03896	calcium-binding protein	2.3		−
GAM04237	Malate:quinone oxidoreductase	2.0	K00116	citrate cycle
GAM06750	REDY-like protein HapK	1.9		−
GAM03632	3-hydroxyacyl-CoA dehydrogenase	1.9	K07516	fatty acid degradation
GAM03631	acyl-CoA dehydrogenase	1.8		−
GAM03633	acetyl-CoA acyltransferase	1.7	K00632	benzoate degradation
GAM04562	PadR family transcriptional regulator	1.7	K10947	PadR family transcriptional regulator
GAM05137	CopG family transcriptional regulator	1.7	K07722	CopG family transcriptional regulator
GAM04124	2-keto-4-pentenoate hydratase	1.7	K02554	degradation of aromatic compounds

^*^KO entries are the defined ortholog groups categorized under the hierarchy of KEGG pathways and BRITE ontologies in Kyoto Encyclopedia of Genes and Genomes Databases (KEGG) (http://www.genome.jp/kegg/)

^**^Manually curated descriptions. See supplementary Table S6 for the full descriptions.

^***^Not defined due to the low nucleotide sequence similarity to KO entries.
