# Supplementary material for: Combination of six enzymes of a marine Novosphingobium converts the stereoisomers of β-O-4 lignin model dimers into the respective monomers
Source: Sci Rep. 2015 Oct 19;5:15105. doi: 10.1038/srep15105 (PMC4609964; doi:10.1038/srep15105)
Supplement: Supplementary Information [file srep15105-s1.pdf]

Supplementary information

Combination of six enzymes of a marine *Novosphingobium* converts the stereoisomers of β-*O*-4 lignin model dimers into the respective monomers

Yukari Ohta*, Shinro Nishi, Ryoichi Hasegawa, and Yuji Hatada

Correspondence: Y. Ohta, Japan Agency for Marine-Earth Science and Technology (JAMSTEC), 2-15 Natsushima, Yokosuka, Kanagawa 237-0061, Japan.

E-mail: ohtay@jamstec.go.jp

Contents

I. Supplementary methods (page 2-14)

II. Supplementary figures page (page 15-28)

III. Supplementary tables (page 29-40)

IV. Supplementary references (page 41-48)

I. Supplementary methods

Synthesis of β-ether-linked model lignin dimers and associated metabolites

1-(4-Hydroxy-3-methoxyphenyl)-2-(2-methoxyphenoxy)-1,3-propanediol (guaiacylglycerol-β-guaiacyl ether; GGGE) and 3-hydroxy-1-(4-hydroxy-3-methoxyphenyl)-2-(2-methoxyphenoxy)-1-propanone ((2-methoxyphenoxy)hydroxypropiovanillone; MPHPV) were synthesized according to the method of Hosoya *et al.* (45). Briefly, the synthesis was initiated by the bromination of commercially available acetovanillone (1-(4-hydroxy-3-methoxyphenyl) -1-ethanone) to produce 2-bromo-1-(4-hydroxy-3-methoxyphenyl) -1-ethanone. Keto aryl ether was formed via the displacement reaction of the bromine with the phenolate ion of guaiacol, affording 1-(4-hydroxy-3-methoxyphenyl)-2-(2-methoxyphenoxy) -1- ethanone. An aldol reaction with formaldehyde was used to produce MPHPV, which was then reduced with $NaBH_4$ to obtain GGGE. 1-(3,4-Dimethoxyphenyl)-2-(2-methoxyphenoxy)-1,3-propanediol (veratrylglycerol-β-guaiacyl ether; VGGE) and 3-hydroxy-1-(3,4-dimethoxyphenyl)-2-(2-methoxyphenoxy)-1-propanone (β-guaiacyl-α-veratrylglycerone; GVG) were synthesized by a similar scheme as used for GGGE with minor modifications according to the description of Picart *et al.* (24). Briefly, acetoveratrone (1-(3,4-dimethoxyphenyl)ethan-1-one) was first brominated to produce bromoacetoveratrone (2-bromo-1- (3,4-dimethoxyphenyl)ethan-1-one). Keto aryl ether was formed by the displacement of bromide with the phenolate ion of guaiacol, affording 1-(3,4-dimethoxyphenyl)-2- (2-methoxyphenoxy)ethan-1-one. An aldol reaction with formaldehyde was used to produce GVG, which was then reduced with $NaBH_4$ to obtain VGGE. The synthesized compounds were characterized and assigned using liquid chromatography/mass spectroscopy (LC/MS) and ^{13}C-NMR. LC/MS data were generated

using a Waters Xevo G2 quadrupole time-of-flight mass spectrometer operated in negative ion ESI mode. The inlet system was a Waters Acquity H-class UPLC system and was operated at a flow rate of 0.4 mL/min using a BEH C18 reverse phase column (1.8-μm particle size, 100 × 2.1 mm; Waters) using the mobile phase gradients A (2 mM sodium acetate and 0.05% formic acid) and B (95% acetonitrile/H_2O) under the following conditions: from 0–6 min, 95%-5% A with B as the remainder; and from 6–7 min, 100% B. The eluate was monitored at 270 nm using a Waters photo diode array (PDA) eλ detector. Data were acquired over the mass range of 100 to 1000 Da with a 0.45-s scan time using a desolvation temperature of 500 °C, source temperature of 150 °C and cone voltage of 30 V. Measured mono-isotopic mass (319.1 m/z) was consistent with the calculated masses (M-H^+ GGGE/ $C_{17}H_{19}O_6$; 319.1, VGGE/ $C_{18}H_{21}O_6$; 333.1) from the molecular formulas of each compound (GGGE/ $C_{17}H_{20}O_6$; 320.1, VGGE/ $C_{18}H_{22}O_6$; 334.1). ^{13}C NMR spectra of GGGE (Figure S7) and VGGE (Figure S8) were recorded on a Varian Inova 400- and 500-MHz spectrometer (Agilent Technologies, Santa Clara, CA, USA). Synthesized GGGE and VGGE had the following characteristic peaks:

[GGGE] ^{13}C-NMR (101 MHz, $CDCl_3$) δ [ppm] 151.4 (C3´); 147.7 (C4´); 146.7 (C3); 145.6, 145.1 (C4); 131.8, 131.5 (C1); 124.3,124.2 (C1´); 121.7, 121.7 (C6´); 121.1, 121.0 (C6); 120.3, 119.1 (C5´); 114.3, 114.3 (C5); 112.2 (C2´); 109.5, 108.7, (C2); 89.6 (Cβ-threo), 87.4 (Cβ-erithro); 74.0(Cα-threo), 72.8 (Cα-erithro); 61.1(Cγ-threo), 60.8 (Cγ-erithro); and 56.0 / 55.9 (3´-OMe / 3-OMe).

[VGGE] ^{13}C-NMR (101 MHz, CDCl3) δ [ppm] 151.5(C3'-erithro); 151.2(C3'-threo); 149.0(C3); 148.9(C4-threo); 148.4 (C4-erithro); 147.6 (C4'-threo); 146.8 (C4'-erithro);132.5 (C1-erithro); 132.1 (C1-threo); 124.2, 124.1 (C1'); 121.7, 121.6 (C6'); 121.0 (C5'); 119.6 (C6-threo); 118.4 (C6-erithro); 112.1 (C2'); 111.0 (C5); 109.8 (C2-threo); 109.2 (C2-erithro); 89.4 (Cβ-threo); 87.3 (Cβ-erithro); 73.9 (Cα-threo); 72.6 (Cα-erithro); 61.0 (Cγ-threo); 60.7 (Cγ-erithro); and 55.9 (C-OMe).

Numbering of the atoms followed the scheme used in an earlier study (31). The ^{13}C-NMR spectrum of synthetic GGGE (Figure S7) and VGGE (Figure S8) matched those deposited in the NMR Database of Lignin and Cell Wall Model Compounds (http://ars.usda.gov/SP2UserFiles/Place/36553000/software/NMR/NMR_DB_11-2004.pdf) (46).

The ratios of stereoisomers in synthetic GGGE and MPHPV were determined by chiral chromatography based on the peak areas of the isomers. GGGE contained α(*S*)β(*R*) GGGE, α(*R*)β(*S*) GGGE, α(*S*)β(*S*) GGGE, and α(*R*)β(*R*) GGGE at a ratio of 1:1:3:3. MPHPV contained β(*R*) MPHPV and β(*S*) MPHPV at a ratio of 1:1 (Figure S9). The analytical conditions are described below in the Chiral chromatography section.

For the structural analysis of the unidentified metabolite from GGGE, 3-hydroxy-1-(4-hydroxy-3-methoxyphenyl)-1-propanone (guaiacyl hydroxyl propanone; GHP) was chemically synthesized via an aldol reaction. For the synthesis, NaOH 9.6 g (0.24 mol) was dissolved in 850 mL water in a 1-l Erlenmeyer flask, to which 33.2 g (0.20 mol) acetovanillone followed by 19.5 g (0.24 mol) 37% formalin were added. The resulting reaction mixture was incubated at 40 °C for 3.5 h. Aqueous HCl was used to adjust the pH of the reaction mixture to approximately 3, and the unreacted acetovanillone crystal deposit was then removed by filtration. The filtrate was extracted twice with 300 mL ethyl acetate, which was then removed, yielding 9.3 g of crude product consisting of approximately 12% GHP. The crude product was purified with silica gel (Wakogel C-200, Wako, Osaka, Japan) using ethyl acetate:toluene (1:4), followed by ethyl acetate:toluene (1:1). After removal of the solvent from the GHP-containing fractions, the obtained residue (1.6 g) was recrystallized from the ethyl acetate:toluene (1:1) to yield 0.8 g GHP with an HPLC purity of 99%. GHP was characterized by 2D COSY, HSQC, and HMBC. NMR spectra were recorded on a Varian Inova 500-MHz spectrometer (Agilent Technologies). The NMR spectra had the following signals: ^{1}H-NMR

(500 MHz, CDCl3) δ [ppm]: 7.56-7.54 (m, 2H, H2/H6), 6.96 (d, 1H, J = 8.5 Hz, H5), 6.15 (s, 1H, phenol-OH), 4.04-4.01 (m, 2H, Hγ), 3.96 (s, 3H, 3-OMe), 3.19 (t, 2H, J = 5.3 Hz, Hβ), and 2.74 (t, 1H, γ-OH) (Figure S10a); ^{13}C-NMR (126 MHz, $CDCl_3$) δ [ppm]: 199.1 (Cα); 150.8 (C4); 146.7 (C3), 129.7 (C1); 123.7 (C6); 113.9 (C5); 109.6 (C2); 58.3 (Cγ); 56.1 (3-OMe), and 39.8 (Cβ) (Figure S10b).

Strains and media. *Novosphingobium* sp. strain MBES04 (NITE AP-01797) was grown aerobically with shaking at 30 °C in a basal medium consisting of Luria-Bertani (LB) medium supplemented with 5 mM $MgSO_4$. For testing carbon utilization, a defined mineral medium containing 1 mM of the test substrate as the sole carbon source was used. The mineral medium (100 mL) consisted of basal salt solution (33.9 g Na_2HPO_4, 15.0 g KH_2PO_4, 10.0 g NaCl, and 5.0 g NH_4Cl per liter of deionized H_2O), 0.5 mL 1 M $MgSO_4$, 1 mL of 0.25% (w/v) of Daigo's IMK medium (Wako), 1 mL trace vitamins solution, 1 mL of 100 mM substrate stock solution, and 86.5 mL deionized H_2O. The trace vitamin solution was prepared according to Balch *et al.* (51). Prior to use, the medium was sterilized using a 0.22-μm membrane filter. Substrate stock solutions of 100 mM GGGE, MPHPV, synaptic acid, ferulic acid, caffeic acid, 4-hydroxybenzoic acid, syringic acid, vanillic acid, vanillin, protocatechuic acid, and chlorogenic acid were prepared using N,N-dimethylformamide (DMF) as a solvent. Stock solutions of 100 mM sodium benzate arabinose and xylose were prepared in deionized H_2O. Mineral medium containing 1 mM glucose with/without 1% (v/v) DMF was used as a positive control for growth. The growth of strain MBES04 was not affected by supplementation of the medium with 1% (v/v) DMF.

Metabolism of a crude extract from milled wood. *Quercus myrsinifolia* sawdust was milled at 25,000 rpm for 2 min using a Wander blender (D3V-10, Osaka Chemical, Osaka,

Japan). The coarse grain was removed by passing the material through a 0.1-mm mesh sieve. A total of 10 g milled wood grain was immersed in 1 L dioxian-water (96:4) for 2 days at room temperature. The extract was recovered by filtration and dried under vacuum to obtain a crude lignin-rich material, which was then suspended in water at 0.4% (w/v) and autoclaved at 120 °C for 15 min. The suspension was filtered through a 0.22-μm membrane to obtain the water-soluble fraction, which was designated as WDM (water-soluble fraction of dioxan extract from milled wood). A quarter volume of WDM was added to basal medium as a low-molecular-weight lignin containing crude natural materials. Strain MBES04 was cultured using 10 mL WDM-supplied medium in triplicate. After 48-h cultivation, the culture broth was centrifuged at 10,500 × *g* for 10 min to remove all cells and debris, and the obtained supernatant was analyzed by LC/MS. Control experiments were performed in triplicate using basal medium containing WDM without inoculation of strain MBES04 (control 1), and using basal medium without WDM, but with inoculation of the strain (control 2). All LC/MS loading data were analyzed with multivariate statistics using MarkerLynks XS software (Waters). An OPLS-discriminant model was constructed and visualized in an S-plot to detect differences between the data obtained from the WDM-supplemented culture medium and those from the control experiments. Ten MS ions with high loadings (>0.05) and correlations (>0.9) were selected as possible metabolites from WDM and were used for quantification based on the peak area in the MS chromatograms. Metabolites were identified by comparing the retention times and MS spectral patterns with those of GHP and SHP standards. Authentic SHP was purchased from Tokyo Fine Chemicals (Tokyo, Japan).

Assessment of oxidase and peroxidase activities of strain MBES04. The supernatant of 48-h cultures of strain MBES04 grown in WDM-supplemented basal medium was used for the assessment of oxidase and peroxidase activities of the strain. Oxidase activity

was assayed according to the method described in the literature for laccase (48) with minor modifications. Briefly, 0.5 mM 2,2'-azino-bis(3-ethylbenzothiazoline-6-sulphonic acid) (ABTS) and 1 mM 2,6-dimethoxyphenol (DMP) were used as substrates in reaction mixtures with and without 0.5 mM each of the divalent metal salts of $FeSO_4$, $CuSO_4$, and $MnSO_4$. After adding 40 μL of the culture supernatants to the assay mixtures to make a total volume of 200 μL, increases in absorbance at 420 and 480 nm for the ABTS and DMP assays, respectively, were monitored every hour for 4 h with a Powerscan HT microplate reader (Dainippon Pharmaceutical) at 25 °C. Peroxidase activity was assayed in the presence of 0.1 mM H_2O_2 using the same substrates and metal ions for the oxidase assays. Uninoculated medium incubated under the same conditions as the test cultures was used as a control for abiotic-induced changes in the absorbance.

Preparation of expression plasmids and enzyme purification. The whole-genome shotgun sequence of strain MBES04 was previously determined by our group (28). A total of 124 contigs were deposited at DDBJ/EMBL/GenBank under the accession numbers BBNP01000001 to BBNP01000124. Candidate GGGE-metabolizing genes of strain MBES04 were identified by querying all detected ORFs in the MBES04 draft genome with known GGGE-metabolizing genes of *Sphingobium* sp. SYK-6 (accession numbers NC_015976/ Gene ID; BAK65539, BAK65541, BAK65540, BAK65542, BAK68041, BAK68265, BAK68263, and BAK67935) using BLASTP with the following thresholds: coverage >60%, identity >25%, and similarity >50% (56). DNA fragments containing possible genes encoding GGGE-metabolizing enzymes and the expression vector pRSET A (Life Technologies, Carlsbad, CA, USA), which was used to add a His × 6 tag at the N-terminus of the target protein, were amplified by polymerase chain reaction (PCR) using PrimeSTAR GXL DNA polymerase (Takara Bio, Ohtsu, Japan) and the primer sets listed in Table S1. The amplicons

of each ORF and the vector were ligated and cloned into competent *E. coli* strain BL21(DE3)pLysE cells using an In-Fusion HD Cloning Kit (Takara Bio) according to the supplier’s instructions. The constructed plasmids were extracted and purified from cells using a High Pure Plasmid Isolation Kit (Roche Diagnostics, Basel, Switzerland). The nucleotide sequences of inserted genes in the plasmid constructs were confirmed using an ABI 3730 XL DNA sequencer (Applied Biosystems, Foster City, CA, USA).

Transformant cultures of *E. coli* strain BL21(DE3)pLysE were grown aerobically overnight with shaking at 37 °C in LB medium and were then subcultured (1:100) into 400 mL LB medium supplemented with 100 μg/mL ampicillin. After 3-h incubation at 16 °C with shaking, 0.5 mM isopropyl β-D-1-thiogalactopyranoside was added to induce protein expression, and the cultures were further incubated overnight at 16 °C and then harvested by centrifugation at 10,500 × *g*. Pelleted cells were resuspended in ∼20 mL TN buffer (50 mM Tris-HCl and 500 mM NaCl, pH 7.5) and were then disrupted by sonication. After the removal of cell debris by centrifugation at 10,500 × *g*, cell lysates were loaded onto a laboratory-packed column containing 10 mL of cOmplete His-tag Purification Resin (Roche Diagnostics). The packed column was washed with 100 mL of 40 mM imidazole in TN buffer, and His-tagged proteins were then eluted with 20 mL of 500 mM imidazole in TN buffer. Collected fractions were desalted by repeated concentration and dilution at 4 °C using a 10,000 molecular weight cut-off centrifugal concentrator (Amicon Ultra-15 Centrifugal Filter Unit; Merck Millipore AG, Zug, Switzerland). The purity of protein preparations was confirmed by SDS-15% PAGE (Figure S2). Protein concentrations were determined using a Protein Assay Kit (Bio-Rad, Hercules, CA, USA).

Analysis of GGGE metabolism. Strain MBES04 was grown aerobically overnight with shaking at 30 °C in basal medium and was then subcultured (1:100) into 150 mL of basal

medium supplemented with 0.9 mM GGGE. The cultures were further incubated for 5 days at 30 °C with shaking, and culture supernatants were periodically collected by centrifugation at 10,500 × *g* for 5 min. A 0.1-mL aliquot of each supernatant sample was mixed with 0.9 mL methanol and then centrifuged at 10,500 × *g* for 5 min. The resulting supernatant was collected and analyzed using an Aliance 2796 Liquid Chromatography (LC) system (Waters) equipped with an Xbridge C18 reversed-phase column (3.5-μm particle size, 100 × 4.6 mm; Waters) operated at a flow rate of 1.2 mL/min using the mobile phase gradients A (2 mM sodium acetate and 0.05% formic acid) and C (95% methanol/H_2O) under the following conditions: 0–1 min, 90% A and 10% C, 1–8 min, a decreasing gradient of 90%–10% A with C as the reminder, followed by 8–10 min 100% C. The eluate was monitored at 270 nm using a Waters 2998 PDA detector. The amounts of substrate and metabolites in the culture supernatant samples were calculated based on the area of the corresponding chromatographic peaks. Uninoculated medium incubated under the same conditions as the test cultures was used as a blank sample to assess the effect of the abiotic degradation of GGGE.

Preparation and structural characterization of metabolites produced by strain MBES04. The intermediate compound transiently produced by strain MBES04 was determined to be MPHPV based on molecular mass and retention time (t_R) on LC/MS analysis using synthetic MPHPV as a reference. One of the two major end metabolites was identified to be guaiacol by comparison of the t_R value in reversed-phase column chromatography to that of the authentic compound.

To confirm the structure of the other major metabolite, which had a mass of 195.1 m/z ($M\text{-}H^+$), the metabolite was purified by the following procedure. GGGE (288 mg, 3 mM final concentration) was added to a medium composed of 6 g Daigo artificial seawater (Wako), 0.9 g Difco tryptone peptone, 0.9 g Bacto yeast extract, and 300 mL tap water. The prepared medium

was inoculated with strain MBES04 and was then incubated at 30 °C for 150 h with shaking at 120 rpm. The culture supernatant was collected by centrifugation, adjusted to approximately pH 3 with 10% aqueous HCl, and GHP was then extracted three times with 100 mL ethyl acetate. The ethyl acetate extract was concentrated under reduced pressure to obtain 0.5 g of crude metabolite, which was then purified by silica gel (Wakogel C-200) column chromatography (110 × 21 mm) using ethyl acetate:toluene (1:1) as the eluent. The solvent was removed to yield a total of 140 mg crystals.

The purified metabolites recovered from the culture supernatant and chemically synthesized GHP were analyzed by LC/MS as described above, ^{1}H-NMR at 500 MHz in $CDCl_3$ and ^{13}C-NMR at 126 MHz in $CDCl_3$. Both compounds had identical t_R (2.3 min) values and had masses of 195.1 m/z. The GHP recovered and purified from the culture supernatant had the following characteristics: ^{1}H-NMR (500 MHz, CDCl3) δ [ppm]: 7.56-7.54 (m, 2H, H2/H6), 6.96 (d, 1H, *J* = 8.5 Hz, H5), 6.12 (s, 1H, phenol-OH), 4.02 (t, 2H, *J* = 5.5 Hz, Hγ), 3.96 (s, 3H, 3-OMe), 3.19 (t, 2H, *J* = 5.3 Hz, Hβ), and 2.6-2.9 (s, 1H, γ-OH) (Figure S11a). ^{13}C-NMR (126 MHz, CDCl3) δ [ppm]: 199.1 (Cα); 150.8 (C4); 146.7 (C3); 129.7 (C1); 123.7 (C6); 113.9 (C5); 109.5 (C2); 58.3 (Cγ); 56.1 (3-OMe); and 39.8 (Cβ) (Figure S11b).

SDRs and GSTs reactions of lignin model dimers. The enzymatic conversions of four mixed stereoisomers of GGGE (1.0 mM) were performed with the recombinant enzymes SDR3 (encoded by GAM05523, 10.0 μg/mL) or SDR5 (encoded by GAM05547, 5.0 μg/mL) with NAD sodium salt (2.0 mM) as a cofactor for 16 h at 15 or 25 °C, respectively. The enzymatic conversion of two mixed stereoisomers of MPHPV (1.0 mM) was conducted with one or two enzymes selected from GST3 (encoded by GAM05529, 5.0 μg/mL), GST4 (encoded by GAM05530, 5.0 μg/mL), GST5 (encoded by GAM05531, 50.0 μg/mL) and GST6

(encoded by GAM05532, 5.0 μg/mL) with glutathione (2.0 mM) as a cosubstrate for 16 h at 25 °C.

Biochemical characterization and kinetics of SDRs and GSTs. SDR3 and SDR5 were characterized using 10 mM GGGE as a substrate and 20 mM NAD sodium salt as a cofactor. The formation of the reaction product, MPHPV, after 30-min incubation was determined by HPLC as described above. GST4 and GST5 were characterized using 5 mM MPHPV as a substrate and 10 mM GSH as a cofactor. The formation of the reaction product, guaiacol, was measured by HPLC. The determination of the pH optimum for enzymatic activity was performed using the following buffers (100 mM): 2-(N-morpholino) ethanesulfonic acid (pH 5.5 to 7.0), 3-morpholinopropanesulfonic acid (pH 7.0 to 8.0), N-Tris(hydroxymethyl)methyl-3-aminopropanesulfonic acid (pH 8.0 to 9.0), N-cyclohexyl-2-aminoethanesulfonic acid (pH 9.0 to 10.0), and N-cyclohexyl-3-aminopropanesulfonic acid (pH 10.0 to 11.0). The optimal temperature was determined by measuring the formation of each reaction product after a 30-min incubation at the optimal pH for each enzyme at temperature ranges of 5-45 °C for SDR3 and SDR5, and 15-45 °C for GST4 and GST5. All experiments were performed in triplicate.

Kinetic measurements were conducted for 30 min with the substrates (final concentrations) GGGE and VGGE (0.06 to 5.0 mM), MPHPV (0.06 to 2.5 mM), and GVG (0.06 to 1.5 mM). The highest concentration of each substrate was determined according to the maximum solubility of each compound in the tested reaction mixture. The formation of MPHPV from GGGE by SDR3/SDR5, GVG from VGGE by SDR3/SDR5, and guaiacol from MPHPV and GVG by GST3/GST5 were measured by HPLC. The kinetic experiments were performed in triplicate. The apparent K_m and V_{max} values were calculated from a hyperbolic regression analysis using Hyper32 software (version 1.0.0.; http://homepage.ntlworld.com/john.easterby).

GST activities (10 μg GST3, GST4, GST5, and GST6) toward the commercially available substrates phenethyl isothiocyanate, 1-chloro-2,4-dinitrobenzene and 4-nitrophenyl butyrate were assessed according the method described by Mathieu *et al.* (36). 1,2-Dichloro-4-dinitrobenzene, ethacrynic acid and 4-nitrobenzyl chloride were also used as substrates (37).

Chiral chromatography. The enzymatic reaction mixtures prepared above were loaded onto a Waters Oasis WAX Solid Extraction Cartridge Column and eluted with 60% acetonitrile. The recovered fractions were diluted 3 fold with H_2O to prepare 20% acetonitrile solutions, which were then injected into a CHIRALPAK IE-3 column (4.6 × 250 mm; Daicel Chemical Industries) for separation of the stereoisomers α(*S*)β(*R*) GGGE, α(*R*)β(*S*) GGGE, α(*S*)β(*S*) GGGE, α(*R*)β(*R*) GGGE, β(*R*)MPHPV, and β(*S*) MPHPV. A mixture of acetonitrile and H_2O was used as the mobile phase at a flow rate of 1.0 mL/min. The acetonitrile concentration of the mobile phase was adjusted as follows (the remainder was H_2O): 0–10 min, 20% acetonitrile; 10–15 min, gradient from 20% to 30% acetonitrile; and 15–30 min, 30% acetonitrile. The absorbance of the eluate was monitored at 270 nm using a Waters 2998 PDA detector. The t_R of α(*S*)β(*R*) GGGE, α(*R*)β(*S*) GGGE, α(*S*)β(*S*) GGGE, α(*R*)β(*R*) GGGE, β(*R*)MPHPV, and β(*S*) MPHPV are shown in Figure S9. Peak identification was based on optical rotation, as described by Hishiyama *et al.* (49).

RNA isolation and purification. Strain MBES04 was grown aerobically overnight with shaking at 30 °C in basal medium and was then subcultured (1:100) into 100 mL basal medium supplemented with 1 mM GGGE or MPHPV and further incubated at 30 °C for 6 h. Cells cultured in basal medium without GGGE and MPHPV were used as controls. Cells were collected by centrifugation at 10,500 × *g* for 5 min at 4 °C. RNA was isolated and purified

from the pelleted cells using an RNeasy kit (Qiagen, Valencia, CA, USA) following the manufacturer's manual. Total RNA was eluted in 100 µL RNase-free H_2O, and DNase I digestion of genomic DNA was then performed on a column using RNase-free DNase I (Qiagen) according to the manufacturer's protocol. The sample was then subjected to a second RNeasy purification step. RNA quality in the purified solutions was verified by quantification of the A260/A280 and A260/A230 ratios using an e-Spect spectrophotometer (Malcom, Tokyo, Japan) and by electrophoresis on an Agilent Bioanalyzer to detect intact 16S and 23S rRNAs.

Quantitative PCR (qPCR). Four µg of total RNA was reverse transcribed using the Transcriptor First Strand cDNA Synthesis Kit (Roche Diagnostics) in a total volume of 40 µL. The reaction was diluted 1:20 using water and 5 µL were used in the subsequent qPCR reaction performed with Light Cycler 480 SYBR Green Master Mix (Roche Diagnostics) in a Roche Light Cycler 480. The 16S rRNA gene was used as a reference. The primers used for qPCR are listed in Table S5. All qPCR experiments were performed independently in duplicate.

RNA sequencing and data analysis. RNA sequencing was conducted using a previously described method (50). Briefly, RNA samples were treated with DNase I (Promega, Madison, WI, USA) at a concentration of 1 U/µg of total RNA. rRNA was removed using a Ribo-Zero rRNA Removal Kit (Gram-Negative Bacteria) (Epicentre Biotechnologies, Madison, WI, USA). Following purification, the mRNA was fragmented into small pieces (200-700 nt) using fragmentation buffer. The cleaved RNA fragments were used for first strand cDNA synthesis using reverse transcriptase and random primers. This synthesis reaction was followed by second strand cDNA synthesis using DNA polymerase I and RNase H. The generated cDNA fragments were purified using a QiaQuick PCR Extraction Kit (Qiagen), treated using an end

repair process, and then ligated to adapters. The obtained products were purified, and fragments with an approximate size of 200 bp were selected by agarose gel-electrophoresis. Sequencing libraries were constructed by amplifying the selected fragments by PCR. The quality of the sequencing libraries was assessed using an Agilent Bioanalyzer and ABI Step One Plus Real-Time PCR (Applied Biosystems). The constructed sequencing libraries were sequenced using an Illumina Hiseq 2000 platform at the Beijing Genome Institute (BGI, Shenzhen, China).

RNAseq data analysis was performed by mapping the obtained reads to the strain MBES04 draft genome using the short-read aligner Bowtie (http://bowtie-bio.sourceforge.net) (51), with two mismatches being allowed per read alignment.

Differentially expressed genes from mapped RNA-Seq reads of strain MBES04 cultured in medium supplemented with and without lignin model dimer (1 mM GGGE or 1 mM MPHPV) were statistically identified using the method of the Bioconductor project (52), which included iDEGES for accurate normalization of tag count data (53) and edgeR for examining differential expression of replicated count data (54). Significance was calculated using dispersion values estimated from the two samples, as no replicate was available, and was defined as a P-value of < 0.05 in a negative binomial test following correction for false discovery rate (55). The pathways involved in the physiological response to lignin model dimers were inferred using the KEGG Automatic Annotation Server with manual curation (56).

II. Supplementary figures

Figure S1. Detection of metabolites in the crude extract of milled wood

a

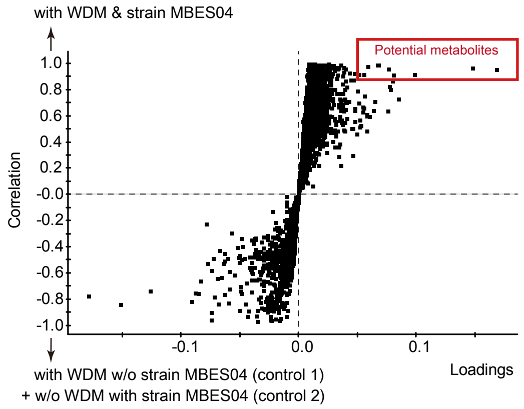

b

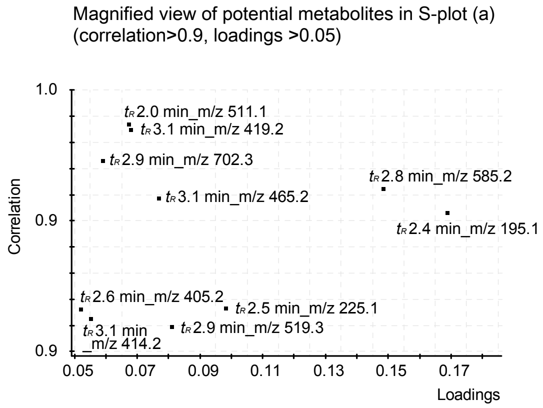

c

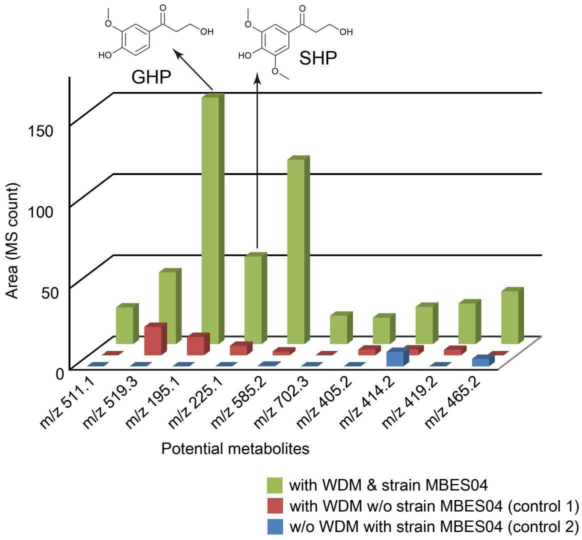

The water-soluble fraction of a dioxan extract from milled wood, Quercus myrsinifolia (WDM), was used for cultivation of strain MBES04. After 48-h cultivation, the supernatant of the culture broth was analyzed by LC/MS. Control experiments conducted using WDM-supplemented medium without inoculation of strain MBES04 (control 1), and uninoculated medium without WDM (control 2) were performed in triplicate. All LC/MS loading data were analyzed using multivariate statistics. An OPLS-discriminant model was constructed and visualized in an S-plot (a) to detect differences between the data obtained from the WDM-supplemented culture media and those from the control experiments described above. Ten MS ions (b) with high loadings (>0.05) and correlations (>0.9) were selected as possible metabolites from WDM and used for quantification based on the peak area in the MS chromatograms (c). Metabolites were identified by comparing the retention times and MS spectra with those of authentic GHP and SHP.

Figure S2, SDS-PAGE of purified SDR (a) and GST (b) recombinant enzymes.

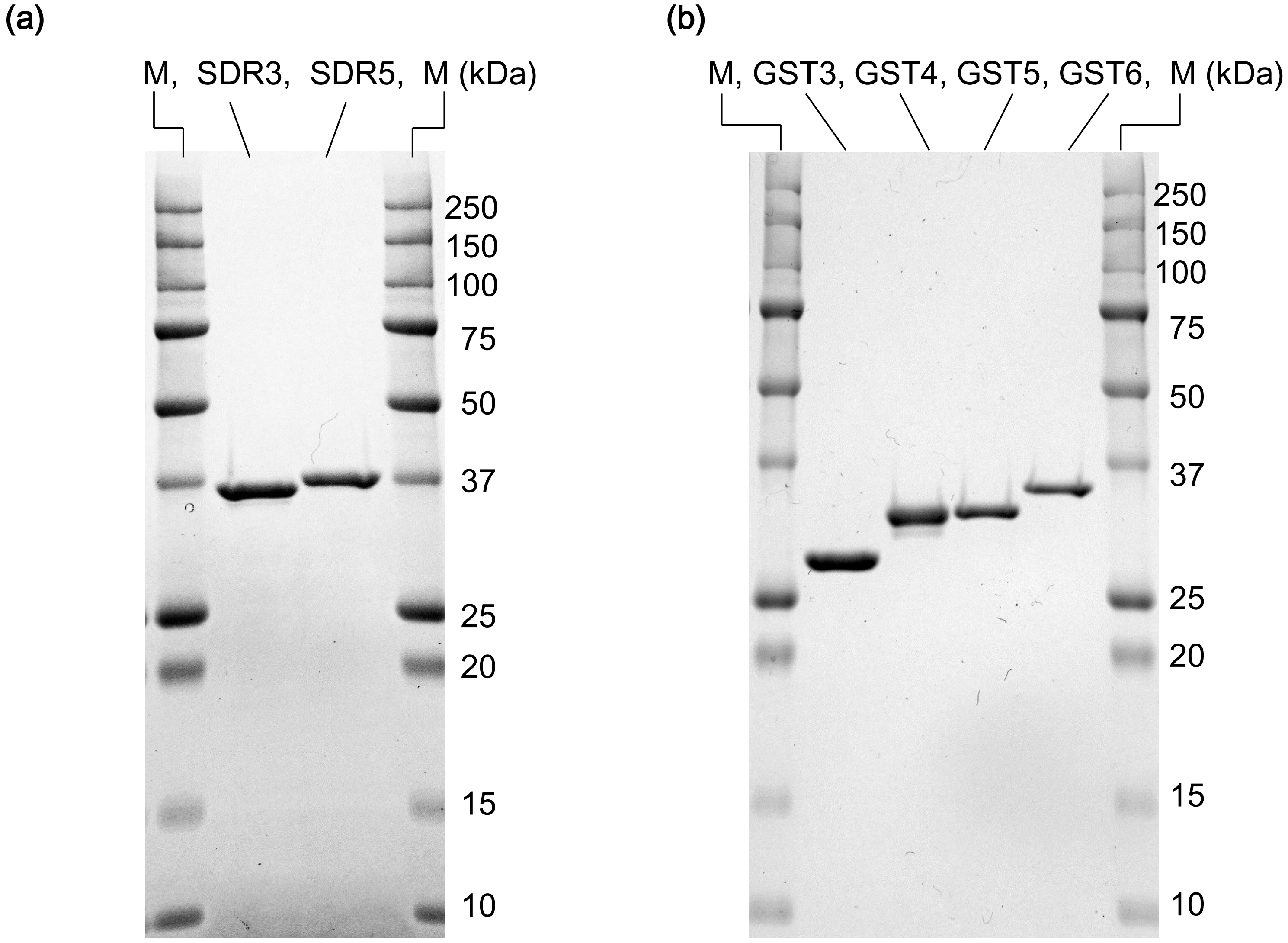

M denotes the size marker.

Figure S3. Determination of the pH and temperature dependences of SDR and GST activities and kinetics with varying substrate concentrations.

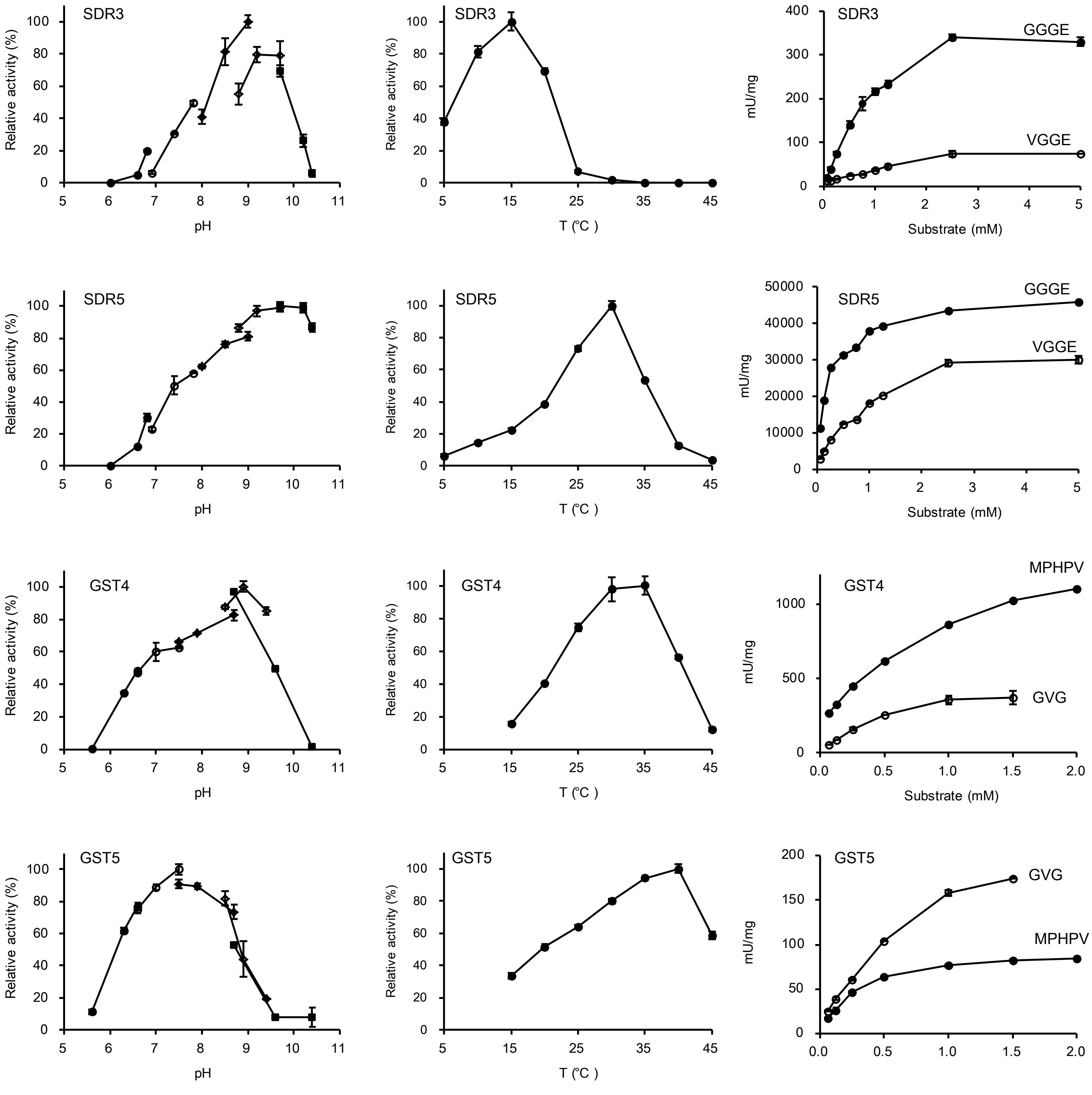

pH and temperature dependences of SDR (SDR3 and SDR5) and GST (GST4 and GST5) activities were evaluated based on the conversion efficiency of GGGE and MPHPV, respectively. The determination of the pH optimum was performed using the following buffers (100 mM): 2-(N-morpholino) ethanesulfonic acid (pH 5.5 to 7.0), 3-morpholinopropanesulfonic acid (pH 7.0 to 8.0), N-Tris(hydroxymethyl)methyl-3-aminopropanesulfonic acid (pH 8.0 to 9.0), N-cyclohexyl-2-aminoethanesulfonic acid (pH 9.0 to 10.0), and N-cyclohexyl-3-aminopropanesulfonic acid (pH 10.0 to 11.0). Values are presented as relative activity with the highest measured activity set to 100%. SDR kinetic experiments were conducted with varying concentrations of GGGE (0.06 to 5.0 mM) and VGGE (0.06 to 5.0 mM). GST kinetic experiments were conducted with varying concentrations of MPHPV (0.06 to 2.0 mM) and GVG (0.06 to 1.5 mM). All reactions were performed in triplicate.

Figure S4. Phylogenetic trees of aligned SDR and GST amino acid sequences.

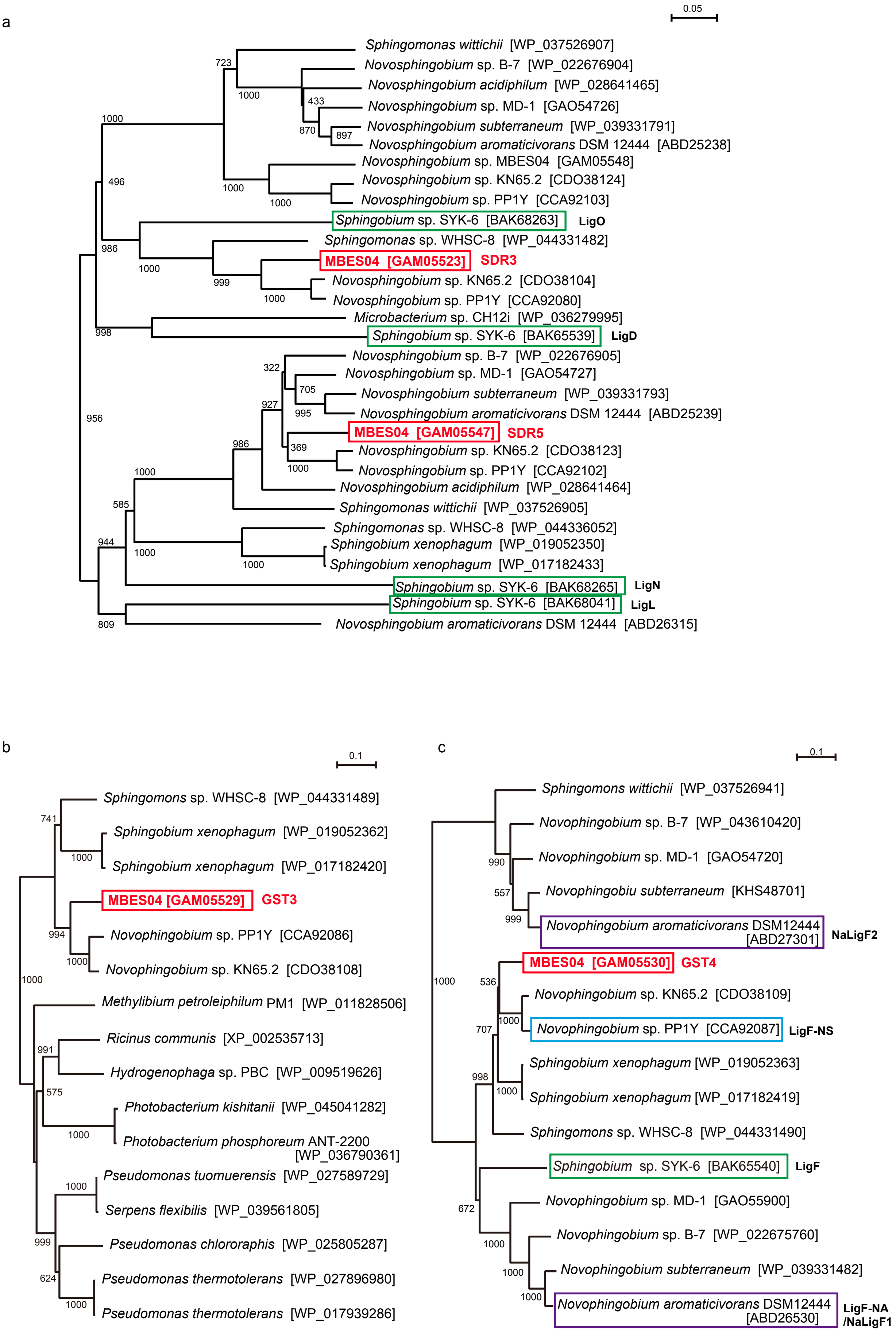

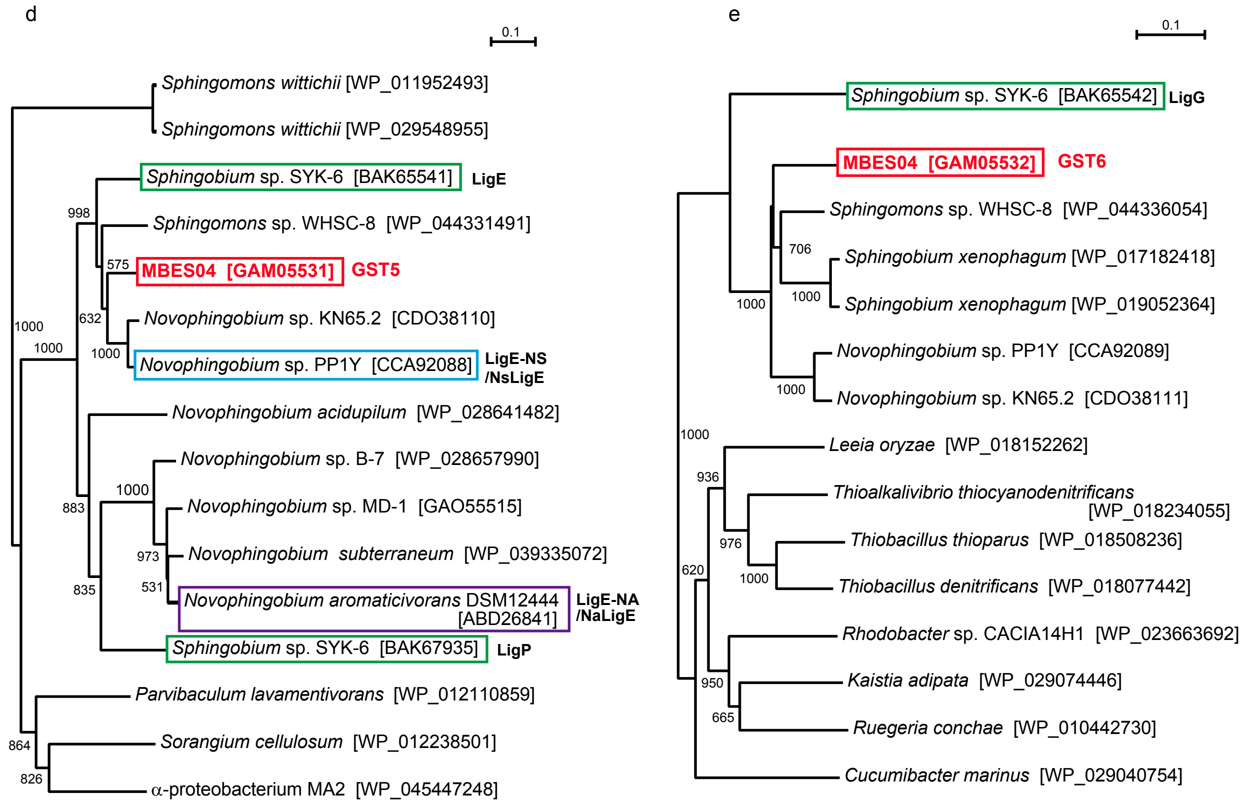

The aligned sequences depicted in (a) included the 15 most similar sequences to each SDR3 and SDR5 amino acid sequence found in the BLASTP database, in addition to the sequences of LigL and LigN. The GST sequences are the 15 most similar sequences to the GST3 (b), GST4 (c), GST5 (d), and GST6 (e) amino acid sequences in the BLASTP database. Enzymes with reported Cα-dehydrogenase, β-etherase, and β-thioetherase activities are indicated by the colored boxes.

Figure S5. Expression levels of GGGE-metabolizing genes in strain MBES04.

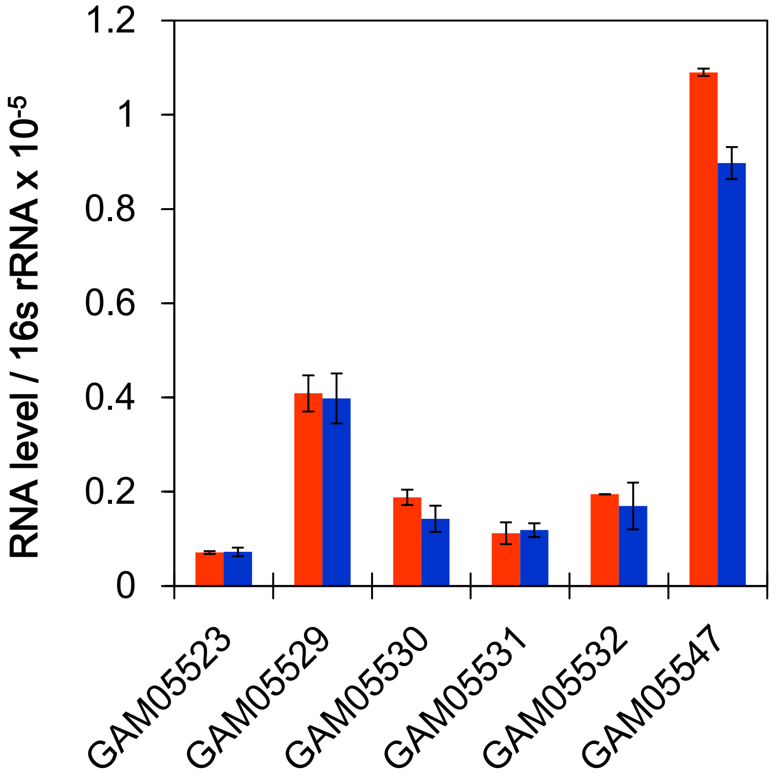

Gene expression levels were measured by qPCR in GGGE-supplemented (red bars) and control (blue bars) conditions. The levels are expressed as the ratio to the expression level of 16S RNA. RNA samples were extracted from two independent cultures for each defined medium condition and quantification of each sample qPCR was conducted in duplicate. Error bars indicate standard deviation.

Figure S6. MA plot of differentially expressed genes (DEG) in the transcriptome analysis of strain MBES04 in response to β-*O*-4 lignin model dimers.

(a) The GGGE-supplemented condition versus control condition

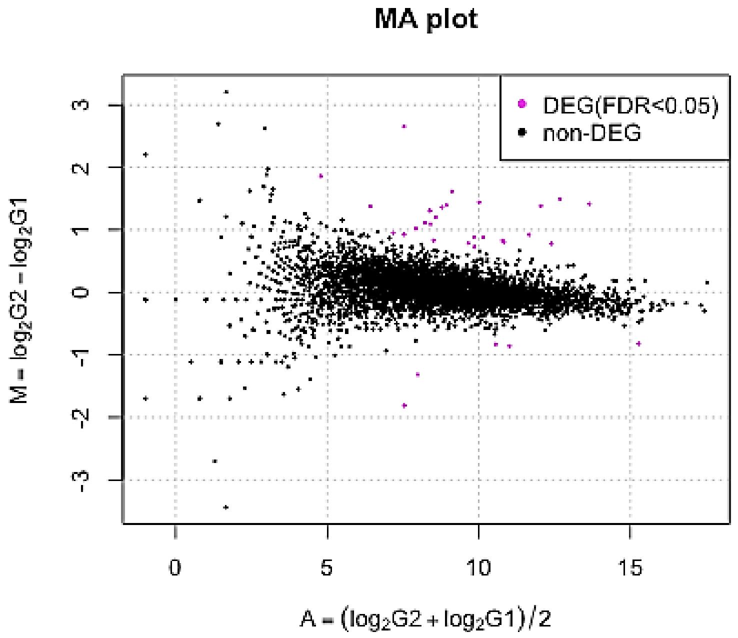

(b) The MPHPV-supplemented condition versus the control condition

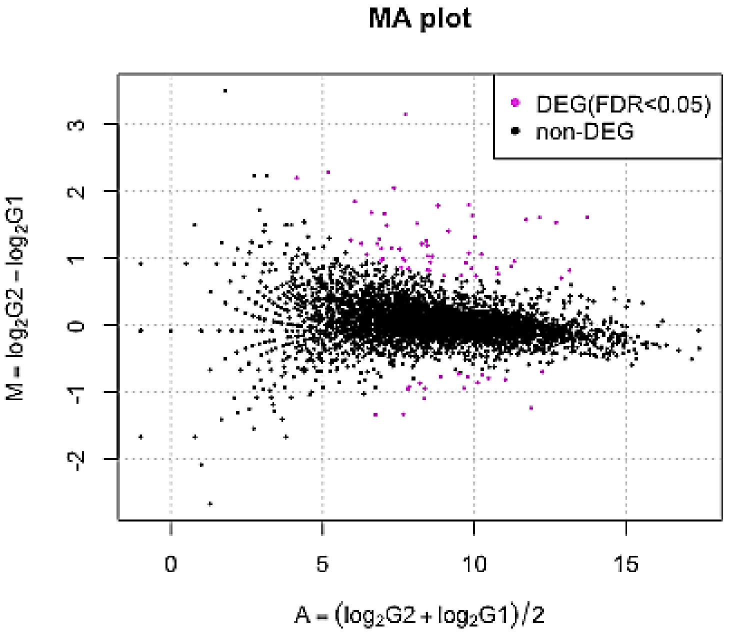

DEGs with significance ($p<0.05$; pink dots) and non-DEGs (black dots) in response to GGGE (a) and MPHPV (b) are shown in the MA plots. The a- and m-values were calculated as follows: a-value=$1/2\log_2(G1G2)=\log_2(G1)+\log_2(G2)$, m-value=$\log_2(G2/G1)=\log_2(G2)-\log_2(G1)$, G1: read counts in the control condition, G2: read counts in response to GGGE or MPHPV.

Figure S7. ^{13}C-NMR spectrum of synthetic GGGE in $CDCl_3$.

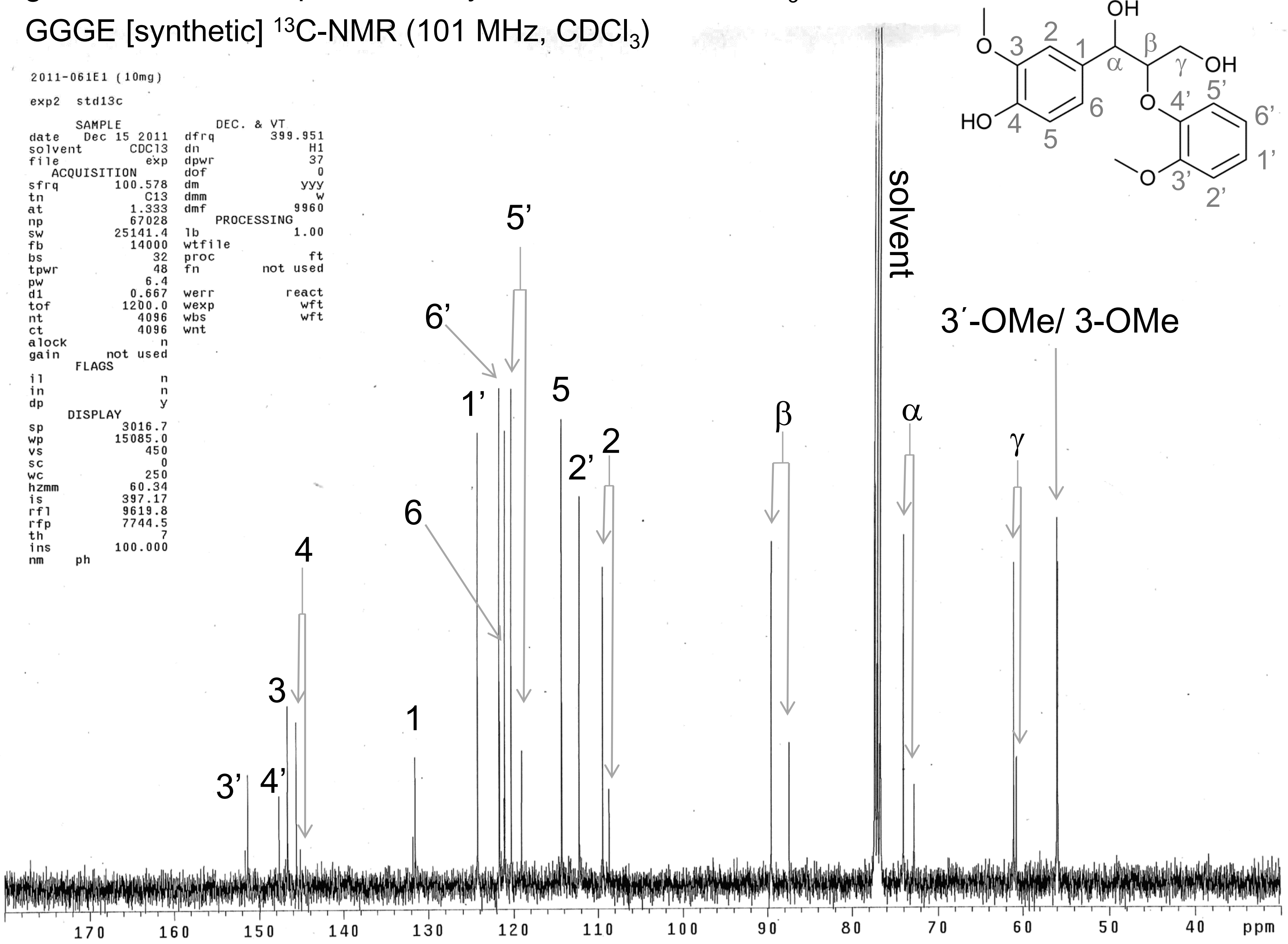

Figure S8. ^{13}C-NMR spectrum of synthetic VGGE in $CDCl_3$.

VGGE [synthetic] ^{13}C-NMR (126 MHz, $CDCl_3$)

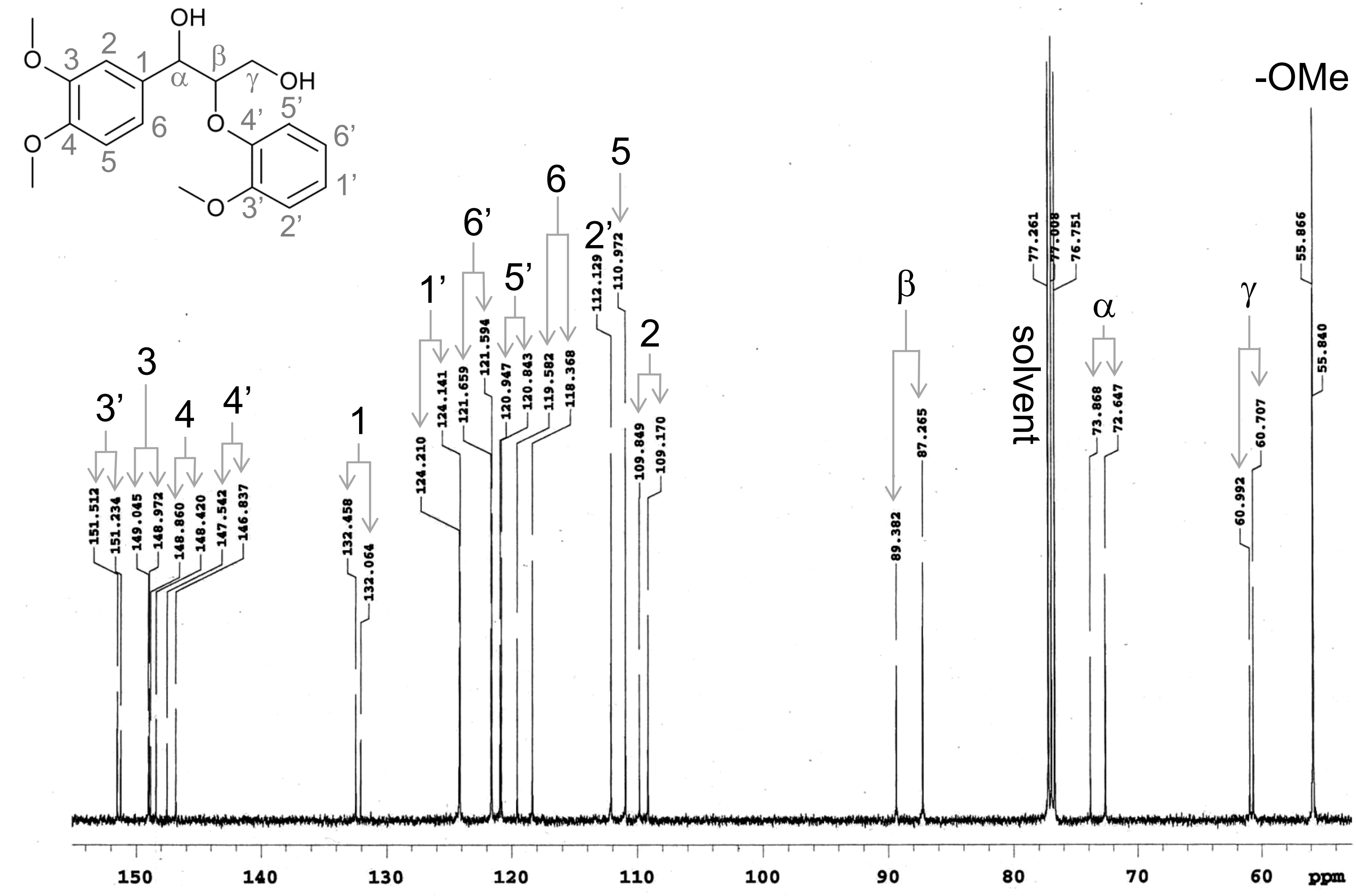

Figure S9. Chiral HPLC chromatograms of synthetic GGGE and MPHPV.

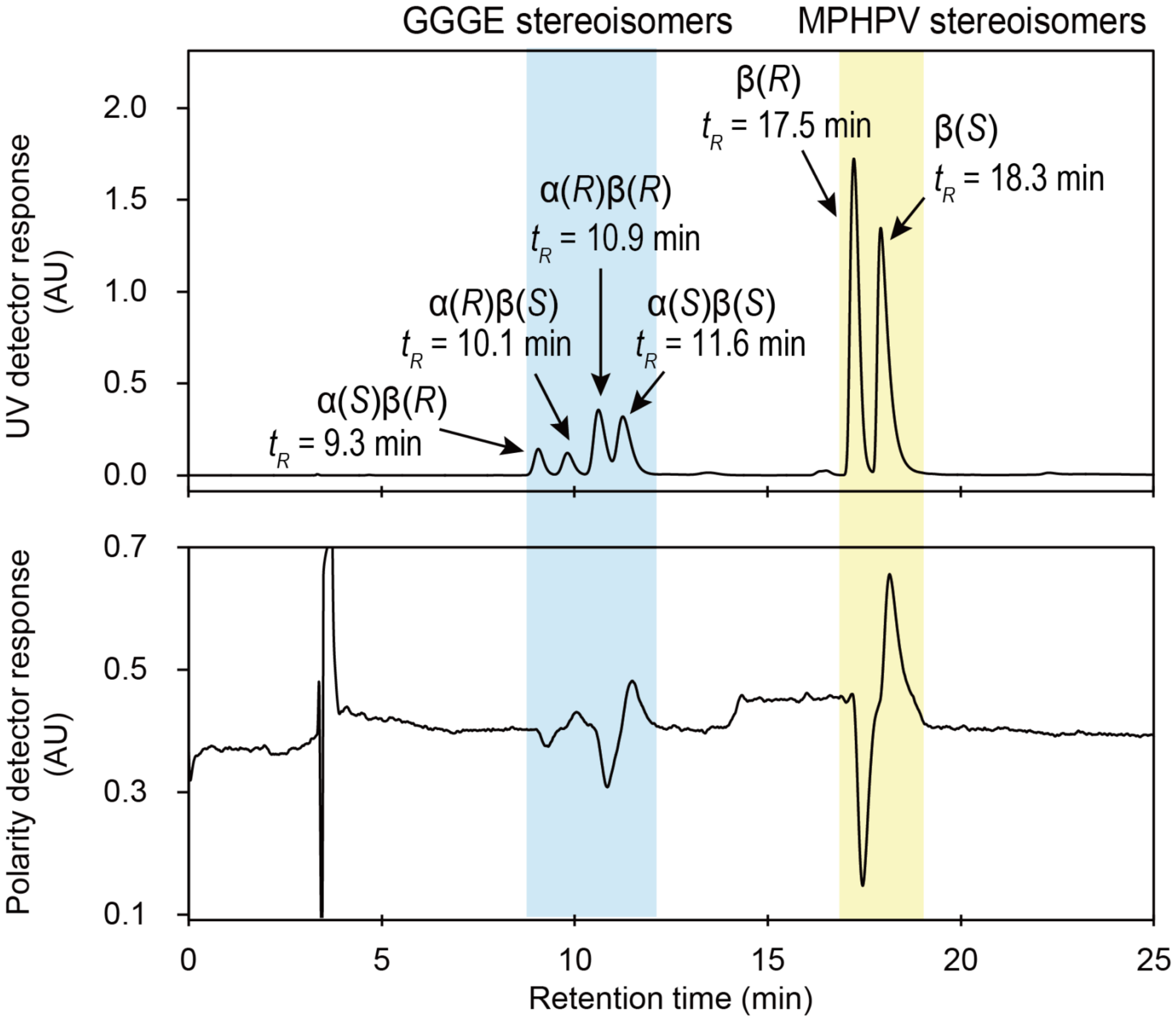

Four stereoisomers of GGGE and two stereoisomers of MPHPV were separated by UV absorbance at 270 nm (top) and optical rotation (bottom). The elution time is indicated below the peak identifications.

Figure S10. ^{1}H-NMR (a) and ^{13}C-NMR (b) spectra of synthetic GHP in $CDCl_3$.

(a) GHP [synthetic] ^{1}H-NMR (500 MHz, $CDCl_3$)

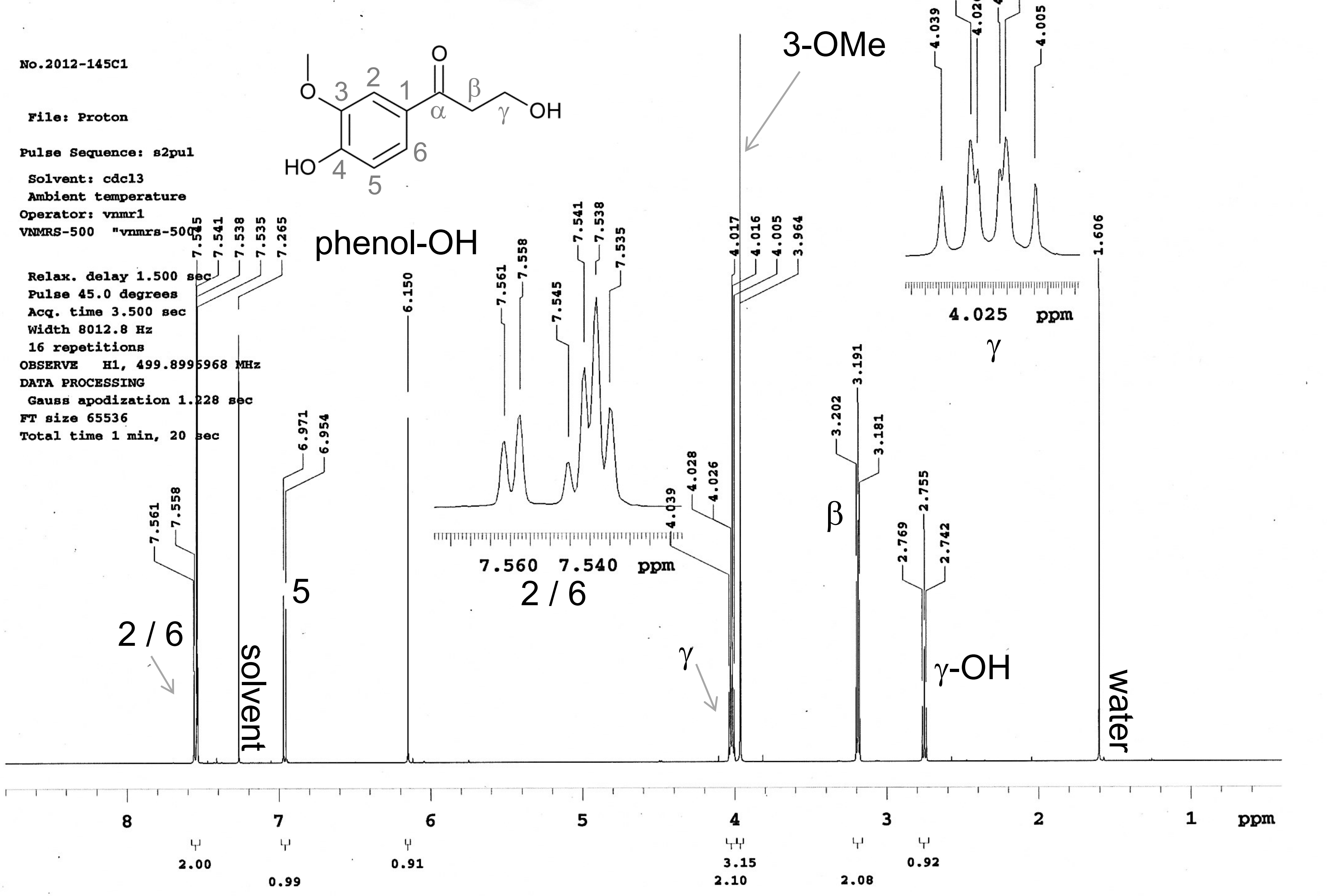

Figure S10, ^{1}H-NMR (a) and ^{13}C-NMR (b) spectra of synthetic GHP in $CDCl_3$.

(b) GHP [synthetic] ^{13}C-NMR (126 MHz, $CDCl_3$)

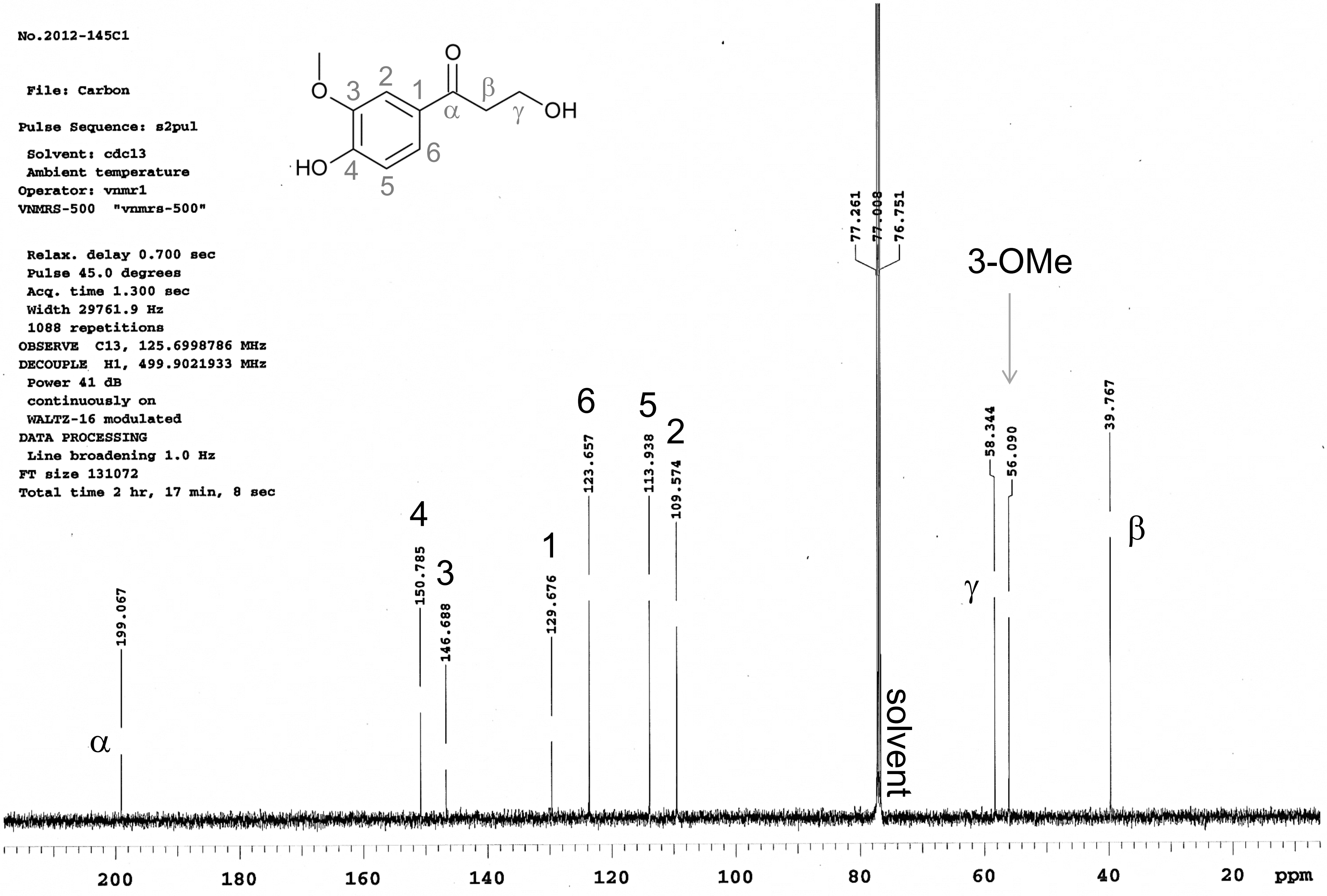

Figure S11. ^{1}H-NMR (a) and ^{13}C-NMR (b) spectra of biologically produced GHP in $CDCl_3$.

(a) GHP [biologically produced] ^{1}H-NMR (500 MHz, $CDCl_3$)

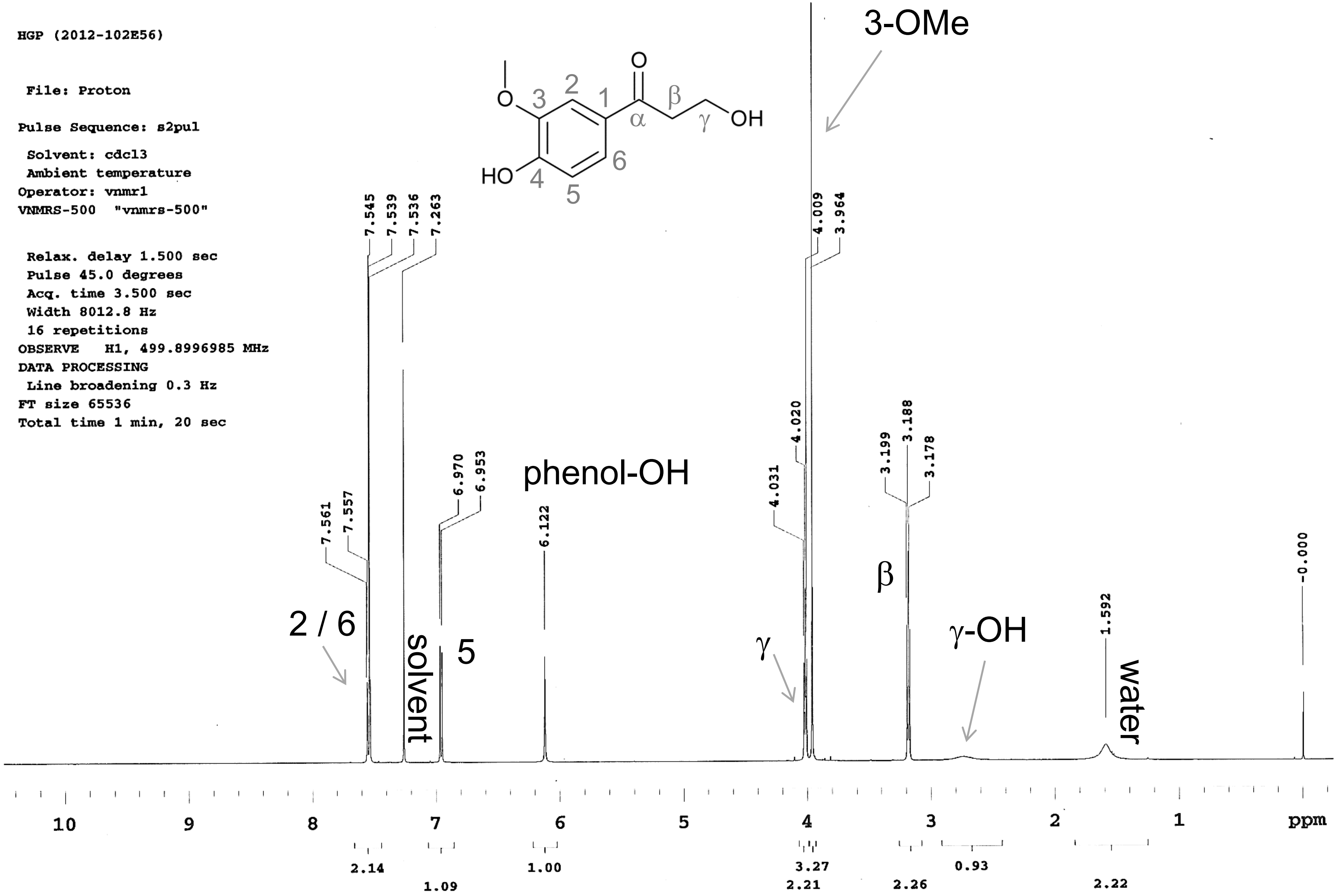

Figure S11. ^{1}H-NMR (a) and ^{13}C-NMR (b) spectra of biologically produced GHP in $CDCl_3$.

(b) GHP [biologically produced] ^{13}C-NMR (126 MHz, $CDCl_3$)

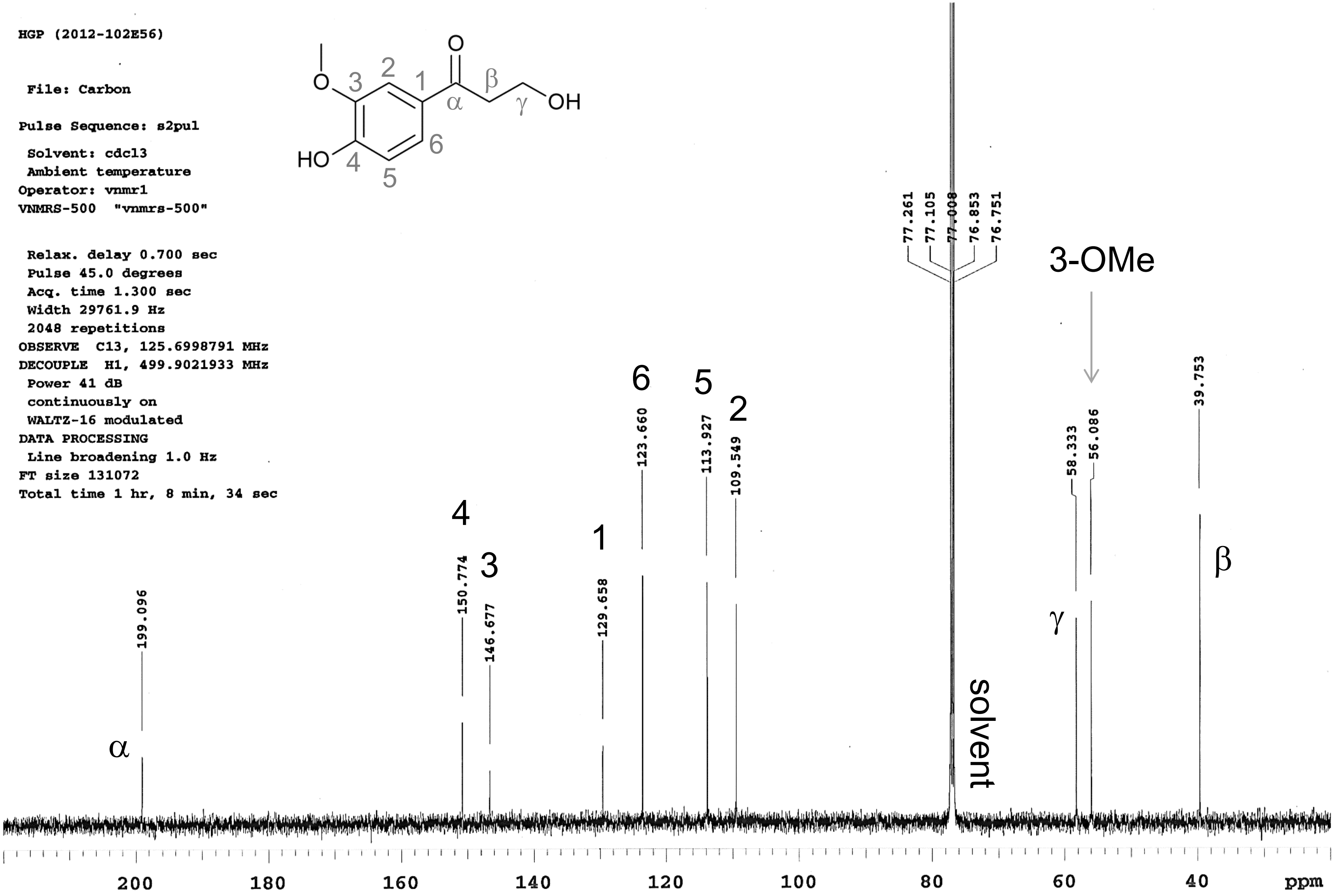

III. Supplementary tables

Table S1. The putative genes for catalase-peroxidase and multicopper oxidase of strain MBES04

Gene ID	BLASTP search nearest hit	Accession	E-value	Identity (%)	Conserved domain
GAM03180	catalase-peroxidase [*Sphingobium* sp. AP49]	WP_007712754	0.0E+00	80	catalase_peroxidase_2
GAM05894	peroxidase [Altererythrobacter atlanticus]	WP_046905285	0.0E+00	78	catalase_peroxidase_2
GAM05893	catalase-peroxidase [*Edwardsiella ictaluri*]	WP_015870368	5.0E-133	82	catalase_peroxidase_1
GAM04190	iron-dependent peroxidase [*Novosphingobium resinovorum*]	EZP74749	1.0E-162	58	predicted iron-dependent peroxidase, COG2837
GAM04037	polyphenol oxidase [*Sphingomonas sp.* WHSC-8]	WP_044335145	3.0E-118	72	multicopper polyphenol oxidase (laccase), yfiH
GAM03576	oxidase [*Citromicrobium bathyomarinum*]	WP_010240406	0.0E+00	98	multicopper oxidase, PRK10965

Table S2. PCR primers used for the gene cloning

Target	Primer name	Sequence
pRSETA	vec_fw	TCTCGAGCTCGGATCC
	vec_rv	CTGGTACCATGGAATTCG
GAM06260	sdr1_fw	GGATCCGAGCTCGAGATGATCAAGGGTATCGAAGG
	sdr1_rv	CGAATTCCATGGTACCAGTCAGAACTCCTGTGCGG
GAM06111	sdr2_fw	GGATCCGAGCTCGAGATGACGAACTGGCTTATCAC
	sdr2_rv	CGAATTCCATGGTACCAGTCAGACCTCGGCGAAG
GAM05523	sdr3_fw	GGATCCGAGCTCGAGATGACACAGGTAAAGGGACG
	sdr3_rv	CGAATTCCATGGTACCAGTCATGCCGTCTTTTCCTC
GAM05534	sdr4_fw	GGATCCGAGCTCGAGATGGGAGAGACGACAAAAC
	sdr4_rv	CGAATTCCATGGTACCAGTCAGGTGAGGTCGGC
GAM05547	sdr5_fw	GGATCCGAGCTCGAGATGCAGGATCTACCGGG
	sdr5_rv	CGAATTCCATGGTACCGCAAGCTGTGTCATGC
GAM05547	sdr6_fw	GGATCCGAGCTCGAGATGACGGGCGGGG
	sdr6_rv	CGAATTCCATGGTACCAGTCAGAGCGCGTTGGC
GAM05529	gst3_fw	GGATCCGAGCTCGAGATGCTGGAACTGTGGACTTC
	gst3_rv	CGAATTCCATGGTACCAGGTAGGTGTGCTCATCGTTCA
GAM05530	gst4_fw	GGATCCGAGCTCGAGATGTTGACGCTGTACAGCTTTG
	gst4_rv	CGAATTCCATGGTACCAGCTCCTCAGGCCTGTGC
GAM05531	gst5_fw	GGATCCGAGCTCGAGATGGCCAAGGACAACC
	gst5_rv	CGAATTCCATGGTACCAGTCAGCTCGCCGTAGC
GAM05532	gst6_fw	GGATCCGAGCTCGAGATGGCATGGGACGATG
	gst6_rv	CGAATTCCATGGTACCAGGATGACGGTGTGCTTCAC

Table S3. Biochemical characterization and kinetics of SDRs (a) and GSTs (b) from strain MBES04 and comparison of those of closely related enzymes.

a

Parameter	Substrate	Enzyme		
		SDR3	SDR5	LigD*
optimal pH	GGGE	9	9-10	9
optimal Temperature		15	30	60
specific activity (mU/mg)		3.4E+02	4.3E+04	1.2E+04
v_{max} (mU)		4.2E+02	4.6E+04	7.9E+03
K_M (mM)		9.7E-01	2.0E-01	1.1E+00
k_{cat} (min^{-1})		1.6E+01	1.7E+03	2.6E+02
k_{cat}/K_M (min^{-1} mM^{-1})		1.6E+01	8.6E+03	2.4E+02
specific activity (mU/mg)	VGGE	7.4E+01	2.9E+04	N.D.
Vmax (mU)		1.0E+02	3.8E+04	N.D.
K_M (mM)		1.5E+00	1.1E+00	N.D.
k_{cat} (min^{-1})		3.9E+00	1.4E+03	N.D.
k_{cat}/K_M (min^{-1} mM^{-1})		2.5E+00	1.3E+03	N.D.

b

Parameter	Substrate	Enzyme								
		GST4	LigF**	LigF-NS**	LigF-NA**	GST5	LigE**	LigE-NS**	LigE-NA**	LigP**
optimal pH	MPHPV	9	N.D.	N.D.	N.D.	7-8	N.D.	N.D.	N.D.	N.D.
optimal Temperature		35	N.D.	N.D.	N.D.	40	N.D.	N.D.	N.D.	N.D.
specific activity (mU/mg)		1.0E+03	N.D.	N.D.	N.D.	8.2E+01	N.D.	N.D.	N.D.	N.D.
v_{max} (mU)		1.3E+03	N.D.	N.D.	N.D.	9.8E+01	N.D.	N.D.	N.D.	N.D.
K_M (mM)		4.7E-01	N.D.	N.D.	N.D.	2.9E-01	N.D.	N.D.	N.D.	N.D.
k_{cat} (min^{-1})		4.5E+01	N.D.	N.D.	N.D.	3.5E+00	N.D.	N.D.	N.D.	N.D.
k_{cat}/K_M (min^{-1} mM^{-1})		9.5E+01	N.D.	N.D.	N.D.	1.2E+01	N.D.	N.D.	N.D.	N.D.
optimal pH	GVG	N.D.	9	9	9	N.D.	9	9	9	9
optimal Temperature		N.D.	25	< 20	25	N.D.	30	< 20	< 20	25
specific activity (mU/mg)		3.7E+02	5.3E+02	3.0E+02	2.9E+03	1.7E+02	2.2E+03	1.4E+02	1.5E+02	6.2E+00
v_{max} (mU)		5.3E+02	N.D.	N.D.	N.D.	2.7E+02	N.D.	N.D.	N.D.	N.D.
K_M (mM)		5.5E-01	N.D.	N.D.	N.D.	7.8E-01	N.D.	N.D.	N.D.	N.D.
k_{cat} (min^{-1})		1.8E+01	N.D.	N.D.	N.D.	9.5E+00	N.D.	N.D.	N.D.	N.D.
k_{cat}/K_M (min^{-1} mM^{-1})		3.2E+01	N.D.	N.D.	N.D.	1.2E+01	N.D.	N.D.	N.D.	N.D.
specific activity (mU/mg)	α-*O*-(β-methyl-umbeliferyl) acetovanillone	N.D.	3.7E+00	1.7E-01	3.1E+00	N.D.	7.0E-03	1.2E-02	2.7E+01	4.1E-02
v_{max} (mU)		N.D.	5.0E+00	2.6E-01	4.9E+00	N.D.	1.0E-02	2.1E-02	3.3E+01	6.7E-02
K_M (mM)		N.D.	3.1E-02	6.0E-02	4.8E-02	N.D.	5.4E-02	6.4E-02	2.4E-02	7.4E-02
k_{cat} (min^{-1})		N.D.	1.6E-01	6.0E-03	1.4E-01	N.D.	3.4E-04	6.7E-04	1.0E+00	2.1E-03
k_{cat}/K_M (min^{-1} mM^{-1})		N.D.	5.2E+00	1.0E-01	2.9E+00	N.D.	6.4E-03	1.0E-02	4.2E+01	2.8E-02

* Data retrieved from Reiter *et al*. (*Green Chem*, 2013, 15, 1373-1381). LigD; GenBank; BAA02030.1 from *Sphingobium* sp. SYK-6

** Data retrieved from Picart *et al*. (*ChemSusChem*, 2014, 7, 3164-3171).
LigF; GenBank;BAK6554 from *Sphingobium* sp. SYK-6, LigF-NS; GenBank; CCA92087 from *Novophingobium* sp. PP1Y, LigF-NS; GenBank; ABD26530, from *N. aromaticivorans* DSM 12444. LigE; GenBank; BAK65541 from *Sphingobium* sp. SYK-6, LigE-NS; GenBank; CCA92088 from *Novophingobium* sp. PP1Y, LigE-NS; GenBank; ABD26841 from *N. aromaticivorans* DSM 12444. LigP; GenBank; BAK67935 from *Sphingobium* sp. SYK-6
N.D. ; not determined.

Table S4. Distribution of possible GGGE-metabolizing genes in the Sphingomonadaceae family

No.	Organism	Origin of isolation	Number of potential homologous enzyms to:						Reference sequene data				Accession number				Literature
			Cα–dehydrogenases		β-etherases		β-thioetherases		Genome sequencing status	Formtat	Number of CDS	Number of nucleotide	Chromosomes		Plasmids		
			SDR3	SDR5	GST4	GST5	GST3	GST6					RefSeq	INSDC	RefSeq	INSDC	
1	*Novosphingobium aromaticivorans* DSM 12444	sediments collected 410 m below the land surface near Allendale, S.C.	**15**	**11**	**3**	**1**	**1**	**0**	complete	amino acid	3,937	4,233,314	NC_007794.1	CP000248.1	NC_009426.1<br>NC_009427.1	CP000676.1<br>CP000677.1	Fredrickson *et al.*, 1991
2	*Novosphingobium* sp. PP1Y	seawater from the harbor of Pozzuoli in Naples, Italy	**13**	**7**	**2**	**1**	**2**	**1**			4,683	5,313,905	NC_015580.1	FR856862.1	NC_015579.1<br>NC_015582.1<br>NC_015583.1	FR856860.1<br>FR856859.1<br>FR856861.1	D'Argenio *et al.*, 2011
3	*Sphingobium chlorophenolicum* L-1	soil	**5**	**7**	**0**	**0**	**3**	**0**			4,072	4,573,221	NC_015593.1<br>NC_015594.1	CP002798.1<br>CP002799.1	NC_015595.1	CP002800.1	Copley *et al.*, 2012
4	*Sphingobium japonicum* UT26S	HCH-contaminated soils	**8**	**2**	**0**	**0**	**4**	**0**			4,394	4,424,862	NC_014006.1<br>NC_014013.1	AP010803.1<br>AP010804.1	NC_014005.1<br>NC_014007.1<br>NC_014009.1	AP010806.1<br>AP010805.1<br>AP010807.1	Nagata *et al.*, 2010
5	*Sphingobium* sp. SYK-6	pond for the treatment of waste liquor from a kraft pulp mill.	**8**	**9**	**2**	**2**	**1**	**1**			4,063	4,348,133	NC_015976.1	AP012222.1	NC_015974.1	AP012223.1	Masai *et al.*, 2007
6	*Sphingomonas sanxanigenens* DSM 19645 = NX02	topsoil collected from a cornfield in Xinhe County, PR China	**6**	**8**	**0**	**0**	**2**	**0**			4,361	6,205,897		CP006644.1	-	-	Huang *et al.*, 2009
7	*Sphingomonas* sp. MM-1	contaminated soil with technical-HCH	**9**	**6**	**0**	**0**	**2**	**0**			5,855	4,633,613	NC_020561.1	CP004036.1	NC_020542.1<br>NC_020562.1<br>NC_020563.2<br>NC_020543.1<br>NC_020544.1	CP004037.1<br>CP004038.1<br>CP004041.2<br>CP004039.1<br>CP004040.1	Tabata *et al.*, 2013
8	*Sphingomonas wittichii* RW1	River Elbe	**9**	**5**	**0**	**1**	**4**	**0**			5,345	5,915,246	NC_009511.1	CP000699.1	NC_009507.1<br>NC_009508.1	CP000700.1<br>CP000701.1	Cua and Stein, 2014
9	*Sphingopyxis alaskensis* RB2256	oceanic surface water	**2**	**4**	**0**	**0**	**3**	**0**			3,195	3,373,713	NC_008048.1	CP000356.1	NC_008036.1	CP000357.1	Hoffmann *et al.*, 2012
10	*Zymomonas mobilis* subsp. *mobilis* ATCC 10988	Mexican pulque fermentations	**2**	**1**	**0**	**0**	**0**	**0**			1,803	2,143,461	NC_017262.1	CP002850.1	NC_017180.1<br>NC_017181.1<br>NC_017182.1<br>NC_017183.1<br>NC_017184.1<br>NC_017185.1	CP002851.1<br>CP002853.1<br>CP002855.1<br>CP002852.1<br>CP002854.1<br>CP002856.1	Pappas *et al.*, 2011
11	*Zymomonas mobilis* subsp. *mobilis* ATCC 29191	palm wine fermentations in Kinshasa, Congo	**1**	**0**	**0**	**0**	**0**	**0**			1,709	2,008,345	NC_018145.1	CP003704.1	NC_018148.1<br>NC_018147.1<br>NC_018146.1	CP003707.1<br>CP003706.1<br>CP003705.1	Desiniotis *et al.*, 2012
12	*Zymomonas mobilis* subsp. *mobilis* NCIMB 11163	British ale-infecting	**4**	**2**	**0**	**0**	**0**	**0**			1,840	2,223,520	NC_013355.1	CP001722.1	NC_013356.1<br>NC_013357.1<br>NC_013358.1	CP001723.1<br>CP001724.1<br>CP001725.1	Kouvelis *et al.*, 2009
13	*Zymomonas mobilis* subsp. *mobilis* str. CP4 = NRRL B-14023	sugarcane fermentations	**2**	**1**	**0**	**0**	**0**	**0**			1,884	2,163,236	NC_022900.1	CP006818.1	NC_022901.1<br>NC_022902.1<br>NC_022903.1<br>NC_022910.1<br>NC_022913.1	CP006891.1<br>CP006893.1<br>CP006894.1<br>CP006895.1<br>CP006892.1	Kouvelis *et al.*, 2014
14	*Zymomonas mobilis* subsp. *mobilis* ZM4 = ATCC 31821	fermenting cane juice	**3**	**1**	**0**	**0**	**0**	**0**			1,748	2,056,363	NC_006526.2	AE008692.2	-	-	Seo *et al*., 2005
15	*Zymomonas mobilis* subsp. *pomaceae* ATCC 29192	sick cider in Bristol, England	**0**	**0**	**0**	**0**	**0**	**0**			1,736	2,061,413	NC_015709.1	CP002865.1	NC_015716.1<br>NC_015715.1	CP002867.1<br>CP002866.1	Kouvelis *et al.*, 2011
16	*Blastomonas* sp. CACIA14H2	water sample collected from the Tucuruí hydroelectric dam in Pará, Brazil	**4**	**4**	**0**	**0**	**3**	**0**	draft		3,787	4,067,409	-	-	-	-	Lima *et al.*, 2014
17	*Novosphingobium lindaniclasticum* LE124	high-dosage-point HCH dumpsite (450 mg HCH/g soil) in Lucknow, India	**2**	**1**	**0**	**0**	**2**	**0**			5,214	4,857,928	-	-	-	-	Saxena *et al.*, 2013
18	*Novosphingobium nitrogenifigens* DSM 19370	a bioreactor treating nitrogen-deficient pulp and paper-mill effluent,New Zealand	**0**	**1**	**0**	**0**	**2**	**0**			4,566	4,182,647	-	-	-	-	Addison *et al.*, 2007
19	*Novosphingobium pentaromativorans* US6-1	muddy sediments of Ulsan Bay, Republic of Korea	**10**	**7**	**0**	**0**	**2**	**0**			3,801	5,344,974	-	-	NZ_AGFM010 00122.1<br>NZ_AGFM010 00123.1	AGFM010001 22.1<br>AGFM010001 23.1	Lyu *et al.*, 2014
20	*Novosphingobium resinovorum*	-	**8**	**7**	**0**	**0**	**1**	**0**			5,234	6,304,486	-	JFYZ0000000 0.1	-	-	Lim *et al.*, 2007
21	*Novosphingobium* sp. AP12	cottonwood rhisosphere	**12**	**7**	**0**	**0**	**1**	**0**			5,737	5,611,617	-	AKKE000000 00.1	-	-	Brown *et al.*, 2012.
22	*Novosphingobium* sp. Rr 2-17	grapevine crown gall tumor	**4**	**1**	**0**	**0**	**1**	**0**			4,302	4,539,029	-	AKFJ0000000 0.1	-	-	Gan *et al.*, 2012
23	*Sphingobium baderi* LL03	hexachlorocyclohexane (HCH)-contaminated soil from Spolana, Czech Republic	**2**	**3**	**0**	**0**	**2**	**0**			4,187	4,848,286	-	ATIB0000000 0.1	-	-	Kaur *et al.*, 2013
24	*Sphingobium chinhatense* IP26	HCH dumpsite located at Chinhat, Lucknow, India	**7**	**1**	**0**	**0**	**4**	**0**			4,212	5,847,843	-	AUDA000000 00.1	-	-	Niharika *et al.*, 2013
25	*Sphingobium herbicidovorans* NBRC 16415	soil in Vienna, Austria	**8**	**7**	**0**	**0**	**1**	**0**			4,807	6,304,486	-	JFZA0000000 0.2	-	-	Zipper *et al*., 1996
26	*Sphingobium indicum* B90A	sugarcane rhizosphere	**7**	**1**	**0**	**0**	**1**	**0**			4,513	4,082,196	-	AJXQ000000 00.1	-	-	Anand *et al.*, 2012
27	*Sphingobium japonicum* BiD32	-	**3**	**2**	**0**	**0**	**2**	**0**			5,703	4,788,005	-	-	-	-	-
28	*Sphingobium lactosutens* DS20	hexachlorocyclohexane (HCH)-contaminated dumpsite located at Lucknow, Uttar Pradesh, India	**6**	**2**	**0**	**0**	**2**	**0**			4,646	5,360,246	-	ATDP000000 00.1	-	-	Kumar R *et al.*, 2013
29	*Sphingobium quisquiliarum* P25	heavily contaminated (450 mg HCH/g of soil) HCH dumpsite located near Lucknow, India	**4**	**4**	**0**	**0**	**1**	**0**			5,737	4,170,546	-	ATHO000000 00.1	-	-	Kumar SA *et al.*, 2013
30	*Sphingobium* sp. Ant17	oil-contaminated soil collected near Scott Base on Ross Island, Antarctica	**2**	**2**	**0**	**0**	**1**	**0**			3,919	5,238,558	-	JEMV000000 00.1	-	-	Adriaenssens *et al.*, 2014
31	*Sphingobium* sp. AP49	root of Populus deltoides	**4**	**2**	**0**	**0**	**2**	**0**			4,671	4,479,274	-	AJVL0000000 0.1	-	-	Brown *et al.*, 2012
32	*Sphingobium* sp. C100	polycyclic aromatic hydrocarbon (PAH)-degrading consortium, which was enriched from the deep-sea sediment of the Makarov Basin in the Arctic Ocean	**1**	**3**	**0**	**0**	**1**	**0**			5,288	4,776,810	-	AYOY000000 00.1	-	-	Dong *et al.*, 2014
33	*Sphingobium* sp. HDIP04	hexachlorocyclohexane contaminated environment	**7**	**2**	**0**	**0**	**4**	**0**			4,033	4,741,576	-	ATDO000000 00.1	-	-	Mukherjee U *et al*., 2013
34	*Sphingobium ummariense* RL-3	hexachlorocyclohexane (HCH) dumpsite located at Ummari village in Lucknow, India	**6**	**2**	**0**	**0**	**3**	**0**			4,645	4,754,053	-	AUWY00000 000.1	-	-	Kohli *et al.*, 2013
35	*Sphingobium yanoikuyae* ATCC 51230	clinical specimen	**7**	**6**	**0**	**0**	**1**	**0**			5,067	5,532,579	-	AGZU000000 00.1	-	-	Yabuuchi *et al*. 1990
36	*Sphingomonas paucimobilis*	clinical specimen	**3**	**2**	**0**	**0**	**1**	**0**			6,283	4,874,185	-	JFYY0000000 0.1	-	-	Nandy *et al.*, 2013
37	*Sphingomonas* sp. LH128	soil	**10**	**8**	**0**	**0**	**1**	**0**			4,705	6,463,118	-	ALVC000000 00.1	-	-	Fida *et al.*, 2013
38	*Sphingomonas* sp. RIT328	cultivars and Salix viminalis × Salix miyabeana grown in bioenergy	**0**	**1**	**0**	**0**	**2**	**0**			4,002	4,343,511	-	JFYV0000000 0.1	-	-	Gan *et al.*, 2014
39	*Sphingomonas* sp. S17	modern stromatolite community in Socompa Lake (3,800-m altitude), placed near the active volcano Socompa in northwest Argentina	**3**	**2**	**0**	**0**	**1**	**0**			4,014	4,268,406	NZ_AFGG000 00000.1	AFGG000000 00.1	-	-	Eugenia *et al.*, 2011
40	*Sphingomonas* sp. SKA58	seawater	**4**	**3**	**0**	**0**	**1**	**0**			3,835	3,887,535	NZ_AAQG00 000000.1	AAQG000000 00.1	-	-	-
41	*Sphingopyxis* sp. MC1	activated sludge from a waste water treatment plants in Seattle, USA	**2**	**2**	**0**	**0**	**4**	**0**			3,467	3,653,464	NZ_AOUN00 000000.1	AOUN000000 00.1	-	-	-

Continue to the next page

Continued from the previous page

No.	Organism	Origin of isolation	Number of potential homologous enzyms to:						Reference sequene data				Accession number				Literature
			Cα–dehydrogenases		β-etherases		β-thioetherases		Genome sequencing status	Formtat	Number of CDS	Number of nucleotide	Chromosomes		Plasmids		
			SDR3	SDR5	GST4	GST5	GST3	GST6					RefSeq	INSDC	RefSeq	INSDC	
42	*Blastomonas* sp. AAP53	surface water of the Swan Lake, a freshwater desert lake in the Inner Mongolia Autonomous Region, China.	2	3	0	0	2	0				3,616,216	NZ_ANFZ00000000.1	ANFZ00000000.1	-	-	Zeng *et al.*, 2013
43	*Citromicrobium bathyomarinum* JL354	surface water of the South China Sea	4	3	0	0	1	0				3,273,334	NZ_ADAE00000000.1	ADAE00000000.1	-	-	Jiao *et al.*, 2010
44	*Citromicrobium* sp. JLT1363	surface water of the South China Sea	1	0	0	0	1	0				3,117,324	NZ_AEUE00000000.1	AEUE00000000.1	-	-	Zheng *et al.*, 2011
45	*Novosphingobium acidiphilum* DSM 19966	subsurface water of the acidic bog lake, Lake Grosse Fuchskuhle in Brandenburg, Germany	3	3	1	1	1	0				3,708,535	NZ_AUBA00000000.1	AUBA00000000.1	-	-	Glaeser *et al.*, 2009
46	*Novosphingobium nitrogenifigens* DSM 19370	pulp and paper-mill effluents, New Zealand	2	1	0	0	2	0				4,864,130	NZ_AEWJ00000000.1	AEWJ00000000.1	-	-	Addison *et al.*, 2007
47	*Novosphingobium* sp. B-7	steeping fluid of an eroded bamboo strips	3	3	2	1	1	0				4,148,973	NZ_APCQ00000000.1	APCQ00000000.1	-	-	Chen *et al.*, 2012
48	*Sandarakinorhabdus limnophila* DSM 17366	mesotrophic freshwater lake Starnberger See in Bavaria Germany,	1	1	0	0	2	0				3,131,544	NZ_ATVO00000000.1	ATVO00000000.1	-	-	Gich and Overmann., 2006
49	*Sandarakinorhabdus* sp. AAP62	surface water of the Shahu Lake in the Ningxia Hui Autonomous Region, China	1	1	0	0	4	0				2,613,739	NZ_ANFY00000000.1	ANFY00000000.1	-	-	Yonghui *et al.*, 2013
50	*Sphingobium lucknowense* F2	hexachlorocylcohexane (HCH) dumpsite located in Ummari village, Lucknow, India	8	1	0	0	4	0				4,916,599		JANF00000000.2	-	-	Negi *et al.*, 2014
51	*Sphingobium* sp. KK22	phenanthrene enrichment culture of a bacterial consortium that grew on diesel fuel	11	2	0	0	1	0				4,756,373	NZ_BATN00000000.1	BATN00000000.1	-	-	Kunihiro *et al.*, 2013
52	*Sphingobium* sp. YL23	sewage sludge of a domestic wastewater treatment plant	1	1	0	0	1	0				4,486,420	NZ_ASTG00000000.1	ASTG00000000.1	-	-	Hu *et al.*, 2013
53	*Sphingobium xenophagum* NBRC 107872 DNA,	river water, Germany	4	3	2	0	2	1				4,221,110	NZ_BARE00000000.1	BARE00000000.1	-	-	Pal R *et al.*, 2006 Mar
54	*Sphingobium xenophagum* QYY	sludge samples, China	4	3	2	0	2	1				5,357,836	NZ_AKIB00000000.1	AKIB00000000.1	-	-	Qu Y *et al.*, 2013
55	*Sphingobium yanoikuyae* XLDN2-5	petroleum-contaminated soils	6	7	0	0	2	0				3,800,099	NZ_AFXE00000000.1	AFXE00000000.1	-	-	Gai *et al.*, 2011
56	*Sphingomonas echinoides* ATCC 14820	plate contaminant, and defined as Pseudomonas echinoides	2	2	0	0	2	0				4,046,117	NZ_AHIR00000000.1	AHIR00000000.1	-	-	Shin *et al.*, 2012b
57	*Sphingomonas elodea* ATCC 31461	Stone Valley Lake, Pennsylvania	1	1	0	0	1	0				3,869,482	NZ_AGFU00000000.1	AGFU00000000.1	-	-	Videir *et al.*, 2000
58	*Sphingomonas jaspsi* DSM 18422	freshwater sample collected at Misasa in Tottori, Japan	1	2	0	0	1	0				4,180,007		JFBW00000000.1	-	-	Asker *et al.*, 2007
59	*Sphingomonas melonis* C3	Tobacco leaf	2	1	0	0	1	0				4,124,388	NZ_AQUJ00000000.1	AQUJ00000000.1	-	-	Innerebner *et al.*, 2011
60	*Sphingomonas melonis* DAPP-PG 224	melon fruit	1	1	0	0	2	0				2,547,846	NZ_AQZT00000000.1	AQZT00000000.1	-	-	Buonaurio *et al.*, 2002
61	*Sphingomonas melonis* FR1	*Arabidopsis thaliana* fruit	2	1	0	0	1	0				563,360	NZ_ATTG00000000.1	ATTG00000000.1	-	-	Innerebner *et al.* 2011
62	*Sphingomonas phyllosphaerae*	*Arabidopsis thaliana* seed	1	1	0	0	1	0				651,875	NZ_ATTI0000	ATTI0000000	-	-	Innerebner *et*
63	*Sphingomonas phyllosphaerae* FA2	phyllosphere of a leguminous tree, Acacia caven, in central Argentina	2	5	0	0	1	0	draft	nucleotide	not relevant	519,583	NZ_ATYK00000000.1	ATYK00000000.1	-	-	Rivas *et al.*, 2004
64	*Sphingomonas* sp. ATCC 31555	pond water	1	2	0	0	1	0				1,382,646	NZ_ALBQ00000000.1	ALBQ00000000.1	-	-	Wang *et al.*, 2012
65	*Sphingomonas* sp. BHC-A	soil that suffered long-term HCH contamination in an insecticide factory	5	1	0	0	1	0				155,074		JDRU00000000.2	-	-	Xue *et al.*, 2014
66	*Sphingomonas* sp. JGI 0001002-A17	-	1	0	0	0	0	0				969,519	NZ_AUOQ00000000.2	AUOQ00000000.2	-	-	-
67	*Sphingomonas* sp. JGI 0001002-C18	-	0	0	0	0	0	0				590,467	NZ_ATTZ00000000.2	ATTZ00000000.2	-	-	-
68	*Sphingomonas* sp. JGI 0001002-D21	-	0	1	0	0	0	0				4,074,265	NZ_AUNZ00000000.2	AUNZ00000000.2	-	-	-
69	*Sphingomonas* sp. JGI 0001002-I20	-	0	1	0	0	0	0				4,878,673	NZ_AUOO00000000.2	AUOO00000000.2	-	-	-
70	*Sphingomonas* sp. JGI 0001003-C6	-	0	0	0	0	0	0				3,929,644	NZ_AUOV00000000.1	AUOV00000000.1	-	-	-
71	*Sphingomonas* sp. JGI 0001003-D23	-	1	1	0	0	1	0				4,096,005	NZ_AUOL00000000.2	AUOL00000000.2	-	-	-
72	*Sphingomonas* sp. JGI 0001003-H15	-	0	0	0	0	2	0				4,047,966	NZ_AUOP00000000.2	AUOP00000000.2	-	-	-
73	*Sphingomonas* sp. KC8	activated sludge	7	9	0	0	3	0				3,185,459	NZ_AFMP00000000.1	AFMP00000000.1	-	-	Hu *et al.*, 2011
74	*Sphingomonas* sp. Mn802worker	termite	3	2	0	0	2	0				4,662,119	NZ_AORY00000000.1	AORY00000000.1	-	-	Aylward *et al.*, 2013
75	*Sphingomonas* sp. PAMC 26605	Arctic lichen (*Ochrolechia* sp.) that grow on rocks	4	5	0	0	5	0				4,663,101	NZ_AHIS00000000.1	AHIS00000000.1	-	-	Shin *et al.*, 2012a
76	*Sphingomonas* sp. PAMC 26617	Arctic lichen (*Umbilicaria* sp.) on the Svalbard Islands	4	4	0	0	5	0				4,769,930	NZ_AHHA00000000.1	AHHA00000000.1	-	-	Lee *et al.*, 2012b
77	*Sphingomonas* sp. PAMC 26621	Arctic lichen (*Cetraria* sp.) on Svalbard Islands	5	3	0	0	8	0				3,920,967	NZ_AIDW00000000.1	AIDW00000000.1	-	-	Lee *et al.*, 2012a
78	*Sphingomonas* sp. PR090111-T3T-6A	termite	4	2	0	0	5	0				3,920,099	NZ_AORL00000000.1	AORL00000000.1	-	-	Aylward *et al.*, 2013
79	*Sphingomonas* sp. URHD0057	-	0	0	0	0	2	0				3,910,956	NZ_JIAU00000000.1	JIAU00000000.1	-	-	-
80	*Sphingomonas* sp. YL-JM2C	activated sludge in Xiamen, China	11	4	0	0	4	0				2,784,495	NZ_ASTM00000000.1	ASTM00000000.1	-	-	-
81	*Sphingomonas* wittichii DP58	pimiento rhizosphere soils	11	6	0	1	6	0				5,628,887	-	JQMC00000000.1	-	-	Ma *et al.*, 2012
82	*Sphingomonas*-like bacterium B12	-	6	6	0	0	5	0				5,923,019	NZ_BACX00000000.1	BACX00000000.1	-	-	-
83	*Sphingopyxis baekryungensis* DSM 16222	sea water at Baekryung Island in the Yellow Sea, Korea	3	4	0	0	1	0				3,068,208	NZ_ATUR00000000.1	ATUR00000000.1	-	-	Yoon *et al.*, 2005
84	*Zymomonas mobilis* subsp. *mobilis* ATCC 31822	mutant derived from ATCC 31821 isolated from fermenting cane juice	3	1	0	0	0	0				2,036,394	NZ_AMSR00000000.1	AMSR00000000.1	-	-	Zhao N *et al.*, 2012
	Total number of the potential homologs		354	245	14	8	156	4									

The six enzymes identified in strain MBES04 and experimentally validated for their conversion activity of GGGE were used as queries to retrieve protein sequences with sequence similarity in 84 species within the Sphingomonadaceae family and whose genome sequences are
-; Not avairable
The full titles of the referenece literatures cited in this table are avirable in supplementary at the Scientific Reports's web site.

Table S5. List of possible GGGE-metabolizing genes and their distribution in the genomes of selected isolates

a

Organism	Reference enzyme	E-value	Identity %	Similarity %	Start nucleotide position	End nucleotide position	Description	Accession number	CDS name
Novosphingobium aromaticivorans DSM 12444	SDR3	2.00E-75	47	64	842319	843251	short-chain dehydrogenase/reductase SDR	ABD25238.1	Saro_0793
		5.00E-64	44	58	843248	844180	short-chain dehydrogenase/reductase SDR	ABD25239.1	Saro_0794
		3.00E-64	43	58	212005	212925	short-chain dehydrogenase/reductase SDR	ABD24653.1	Saro_0205
		7.00E-58	43	59	1976401	1977243	short-chain dehydrogenase/reductase SDR	ABD26315.1	Saro_1875
		1.00E-57	41	57	742296	743213	short-chain dehydrogenase/reductase SDR	ABD25150.1	Saro_0703
		2.00E-31	37	55	297019	297798	short-chain dehydrogenase/reductase SDR	ABP64526.1	Saro_3667
		4.00E-31	36	52	242308	243177	short-chain dehydrogenase/reductase SDR	ABP64479.1	Saro_3619
		1.00E-26	34	53	1163430	1164230	short-chain dehydrogenase/reductase SDR	ABD25562.1	Saro_1117
		7.00E-26	32	50	279606	280364	short-chain dehydrogenase/reductase SDR	ABP64509.1	Saro_3650
		5.00E-24	31	51	73027	73791	short-chain dehydrogenase/reductase SDR	ABP64332.1	Saro_3472
		1.00E-21	30	50	1617712	1618485	short-chain dehydrogenase/reductase SDR	ABD26000.1	Saro_1560
		3.00E-22	30	52	62816	63553	short-chain dehydrogenase/reductase SDR	ABP64320.1	Saro_3460
		5.00E-23	30	50	149323	150084	short-chain dehydrogenase/reductase SDR	ABP64392.1	Saro_3532
		3.00E-19	29	50	553249	553995	short-chain dehydrogenase/reductase SDR	ABD24959.1	Saro_0512
		7.00E-20	28	50	3428543	3429265	3-oxoacyl-[acyl-carrier-protein] reductase	ABD27647.1	Saro_3212
	SDR5	1.00E-142	80	87	843248	844180	short-chain dehydrogenase/reductase SDR	ABD25239.1	Saro_0794
		3.00E-65	49	64	1976401	1977243	short-chain dehydrogenase/reductase SDR	ABD26315.1	Saro_1875
		4.00E-60	44	61	742296	743213	short-chain dehydrogenase/reductase SDR	ABD25150.1	Saro_0703
		5.00E-52	41	58	842319	843251	short-chain dehydrogenase/reductase SDR	ABD25238.1	Saro_0793
		8.00E-49	40	57	212005	212925	short-chain dehydrogenase/reductase SDR	ABD24653.1	Saro_0205
		2.00E-21	36	51	1617712	1618485	short-chain dehydrogenase/reductase SDR	ABD26000.1	Saro_1560
		5.00E-28	35	53	242308	243177	short-chain dehydrogenase/reductase SDR	ABP64479.1	Saro_3619
		2.00E-23	34	50	297019	297798	short-chain dehydrogenase/reductase SDR	ABP64526.1	Saro_3667
		2.00E-19	32	51	1163430	1164230	short-chain dehydrogenase/reductase SDR	ABD25562.1	Saro_1117
		8.00E-15	28	50	3134479	3135297	short-chain dehydrogenase/reductase SDR	ABD27351.1	Saro_2916
	GST3	3.00E-31	37	52	2803495	2804361	glutathione S-transferase-like protein	ABD27031.1	Saro_2595
	GST4	2.00E-93	63	75	2227684	2228457	glutathione S-transferase-like protein	ABD26530.1	Saro_2091
		2.00E-60	46	62	3087750	3088526	glutathione S-transferase-like protein	ABD27301.1	Saro_2865
		2.00E-47	35	55	3096844	3097614	glutathione S-transferase-like protein	ABD27309.1	Saro_2873
	GST5	1.00E-94	62	72	2573603	2574442	glutathione S-transferase-like protein	ABD26841.1	Saro_2405
	GST6	-	-	-	-	-	-	-	-

b

Organism	Reference enzyme	E-value	Identity %	Similarity %	Start nucleotide position	End nucleotide position	Description	Accession number	CDS name
Novosphingobium sp. PP1Y	SDR3	1.00E-150	83	89	1241135	1242028	short-chain dehydrogenase/reductase SDR	CCA92080.1	PP1Y_AT11594
		4.00E-82	50	65	1266094	1266996	short-chain dehydrogenase/reductase SDR	CCA92103.1	PP1Y_AT11846
		1.00E-64	43	58	1265148	1266083	short-chain dehydrogenase/reductase SDR	CCA92102.1	PP1Y_AT11837
		4.00E-61	39	60	1251645	1252559	short-chain dehydrogenase/reductase SDR	CCA92091.1	PP1Y_AT11698
		2.00E-33	37	52	349486	350274	short-chain dehydrogenase/reductase SDR	CCA90296.1	PP1Y_Mpl3491
		4.00E-36	37	55	1367340	1368152	conserved hypothetical protein	CCA92190.1	PP1Y_AT12862
		2.00E-28	36	51	343595	344314	short-chain dehydrogenase/reductase SDR	CCA90289.1	PP1Y_Mpl3436
		8.00E-26	35	51	921727	922500	oxidoreductase	CCA90773.1	PP1Y_Mpl9257
		4.00E-38	35	51	337025	337873	conserved hypothetical protein	CCA91281.1	PP1Y_AT3242
		2.00E-25	34	51	109303	110085	short-chain dehydrogenase/reductase SDR	CCA90094.1	PP1Y_Mpl1126
		9.00E-24	30	52	552919	553671	short-chain dehydrogenase/reductase SDR	CCA90460.1	PP1Y_Mpl5490
		2.00E-22	30	51	2340725	2341462	short-chain dehydrogenase/reductase SDR	CCA93063.1	PP1Y_AT22066
		4.00E-15	26	50	1448106	1448924	short-chain dehydrogenase/reductase SDR	CCA92257.1	PP1Y_AT13638
	SDR5	1.00E-150	82	90	1265148	1266083	short-chain dehydrogenase/reductase SDR	CCA92102.1	PP1Y_AT11837
		2.00E-60	49	61	1241135	1242028	short-chain dehydrogenase/reductase SDR	CCA92080.1	PP1Y_AT11594
		5.00E-57	42	60	1251645	1252559	short-chain dehydrogenase/reductase SDR	CCA92091.1	PP1Y_AT11698
		6.00E-52	42	60	1266094	1266996	short-chain dehydrogenase/reductase SDR	CCA92103.1	PP1Y_AT11846
		2.00E-23	41	51	921727	922500	oxidoreductase	CCA90773.1	PP1Y_Mpl9257
		5.00E-25	37	54	349486	350274	short-chain dehydrogenase/reductase SDR	CCA90296.1	PP1Y_Mpl3491
		8.00E-23	33	52	552919	553671	short-chain dehydrogenase/reductase SDR	CCA90460.1	PP1Y_Mpl5490
	GST3	1.00E-102	80	86	1246742	1247413	glutathione S-transferase	CCA92086.1	PP1Y_AT11650
		1.00E-34	38	56	1235263	1236030	glutathione S-transferase-like	CCA92074.1	PP1Y_AT11532
	GST4	1.00E-129	85	92	1247655	1248407	glutathione S-transferase-like	CCA92087.1	PP1Y_AT11660
		1.00E-48	36	55	1235263	1236030	glutathione S-transferase-like	CCA92074.1	PP1Y_AT11532
	GST5	1.00E-140	86	90	1248451	1249272	glutathione S-transferase-like	CCA92088.1	PP1Y_AT11664
	GST6	1.00E-122	79	87	1249286	1250119	glutathione S-transferase family protein	CCA92089.1	PP1Y_AT11674

c

Organism	Reference enzyme	E-value	Identity %	Similarity %	Start nucleotide position	End nucleotide position	Description	Accession number	CDS name
Sphingobium sp. SYK-6	SDR3	3.00E-93	57	70	3867577	3868470	C alpha-dehydrogenase	BAK68263.1	SLG_35880
		6.00E-65	44	59	920073	920990	C alpha-dehydrogenase	BAK65539.1	SLG_08640
		2.00E-53	40	57	756721	757554	putative oxidoreductase	BAK65399.1	SLG_07240
		2.00E-54	39	57	3635618	3636487	C alpha-dehydrogenase	BAK68041.1	SLG_33660
		8.00E-58	38	57	1377047	1377961	putative oxidoreductase	BAK65939.1	SLG_12640
		3.00E-50	38	53	3066706	3067590	putative oxidoreductase	BAK67509.1	SLG_28340
		5.00E-54	38	54	3872346	3873281	C alpha-dehydrogenase	BAK68265.1	SLG_35900
	SDR5	3.00E-53	46	60	756721	757554	putative oxidoreductase	BAK65399.1	SLG_07240
		2.00E-58	45	59	3867577	3868470	C alpha-dehydrogenase	BAK68263.1	SLG_35880
		4.00E-58	45	57	3872346	3873281	C alpha-dehydrogenase	BAK68265.1	SLG_35900
		4.00E-52	42	56	1377047	1377961	putative oxidoreductase	BAK65939.1	SLG_12640
		1.00E-52	42	55	3066706	3067590	putative oxidoreductase	BAK67509.1	SLG_28340
		2.00E-50	40	57	3635618	3636487	C alpha-dehydrogenase	BAK68041.1	SLG_33660
		3.00E-46	38	57	920073	920990	C alpha-dehydrogenase	BAK65539.1	SLG_08640

Organism	Reference enzyme	E-value	Identity %	Similarity %	Start nucleotide position	End nucleotide position	Description	Accession number	CDS name
		1.00E-22	34	53	3014885	3015685	short-chain dehydrogenase/reductase SDR	BAK67470.1	SLG_27950
		8.00E-24	34	51	3597576	3598301	SDR-family protein	BAK68006.1	SLG_33310
	GST3	2.00E-33	39	55	421704	422603	glutathione S-transferase	BAK65087.1	SLG_04120
	GST4	6.00E-98	68	78	921070	921843	beta-etherase	BAK65540.1	SLG_08650
		1.00E-54	42	59	755869	756615	glutathione S-transferase	BAK65398.1	SLG_07230
	GST5	1.00E-122	80	87	921965	922810	beta-etherase	BAK65541.1	SLG_08660
		1.00E-104	67	80	3523525	3524370	beta-etherase	BAK67935.1	SLG_32600
	GST6	2.00E-96	65	78	922888	923685	glutathione S-transferase	BAK65542.1	SLG_08670

d

Organism	Reference enzyme	E-value	Identity %	Similarity %	Start nucleotide position	End nucleotide position	Description	Accession number	CDS name
Sphingomonas wittichii RW1	SDR3	1.00E-51	39	54	401363	402232	short-chain dehydrogenase/reductase SDR	ABQ66748.1	Swit_0378
		9.00E-26	36	53	3623724	3624473	short-chain dehydrogenase/reductase SDR	ABQ69640.1	Swit_3294
		1.00E-22	35	51	357327	358085	short-chain dehydrogenase/reductase SDR	ABQ66706.1	Swit_0335
		3.00E-24	35	50	2219321	2220061	short-chain dehydrogenase/reductase SDR	ABQ68341.1	Swit_1981
		3.00E-27	34	50	1107907	1108656	short-chain dehydrogenase/reductase SDR	ABQ67363.1	Swit_0996
		2.00E-26	34	50	3377667	3378434	short-chain dehydrogenase/reductase SDR	ABQ69428.1	Swit_3078
		2.00E-24	33	51	322843	323589	short-chain dehydrogenase/reductase SDR	ABQ66676.1	Swit_0305
		1.00E-24	33	52	1163837	1164631	short-chain dehydrogenase/reductase SDR	ABQ67411.1	Swit_1045
		3.00E-24	32	51	3618469	3619263	short-chain dehydrogenase/reductase SDR	ABQ69634.1	Swit_3288
		2.00E-22	31	50	5053768	5054490	3-oxoacyl-[acyl-carrier-protein] reductase	ABQ70937.1	Swit_4599
		7.00E-20	30	50	2513622	2514392	short-chain dehydrogenase/reductase SDR	ABQ68618.1	Swit_2259
		2.00E-19	28	50	1033586	1034305	short-chain dehydrogenase/reductase SDR	ABQ67296.1	Swit_0929
		5.00E-38	38	51	401363	402232	short-chain dehydrogenase/reductase SDR	ABQ66748.1	Swit_0378
		2.00E-24	36	53	1107907	1108656	short-chain dehydrogenase/reductase SDR	ABQ67363.1	Swit_0996
		2.00E-20	36	50	3623724	3624473	short-chain dehydrogenase/reductase SDR	ABQ69640.1	Swit_3294
		3.00E-21	35	51	1163837	1164631	short-chain dehydrogenase/reductase SDR	ABQ67411.1	Swit_1045
		3.00E-26	34	51	1122273	1123010	short-chain dehydrogenase/reductase SDR	ABQ67374.1	Swit_1007
		3.00E-21	33	50	2513622	2514392	short-chain dehydrogenase/reductase SDR	ABQ68618.1	Swit_2259
	SDR5	-	-	-	-	-	-	-	-
	GST3	9.00E-50	45	64	3794267	3794965	Glutathione S-transferase, N-terminal domain	ABQ69803.1	Swit_3457
		8.00E-43	45	60	4580162	4580845	Glutathione S-transferase, N-terminal domain	ABQ70503.1	Swit_4163
		3.00E-36	40	58	240932	241636	Glutathione S-transferase, N-terminal domain	ABQ66605.1	Swit_0234
		1.00E-37	39	55	1850550	1851272	Glutathione S-transferase, N-terminal domain	ABQ68027.1	Swit_1664
	GST4	-	-	-	-	-	-	-	-
	GST5	1.00E-49	41	59	1837745	1838458	hypothetical protein	ABQ68015.1	Swit_1652
	GST6	-	-	-	-	-	-	-	-

e

Organism	Reference enzyme	E-value	Identity %	Similarity %	Start nucleotide position	End nucleotide position	Description	Accession number
Novosphingobium acidiphilum DSM 19966	SDR3	3.00E-79	47	63	10570	11424	Novosphingobium acidiphilum DSM 19966 G404DRAFT_scaffold00015.15_C, whole genome shotgun sequence	gi|523617949|gb|AUBA01000015.1|
		2.00E-64	45	59	9641	10396	Novosphingobium acidiphilum DSM 19966 G404DRAFT_scaffold00015.15_C, whole genome shotgun sequence	gi|523617949|gb|AUBA01000015.1|
		2.00E-25	32	50	37864	38412	Novosphingobium acidiphilum DSM 19966 G404DRAFT_scaffold00001.1_C, whole genome shotgun sequence	gi|523617984|gb|AUBA01000001.1|
	SDR5	1.00E-156	78	86	9641	10552	Novosphingobium acidiphilum DSM 19966 G404DRAFT_scaffold00015.15_C, whole genome shotgun sequence	gi|523617949|gb|AUBA01000015.1|
		2.00E-58	40	60	10570	11340	Novosphingobium acidiphilum DSM 19966 G404DRAFT_scaffold00015.15_C, whole genome shotgun sequence	gi|523617949|gb|AUBA01000015.1|
		7.00E-22	31	51	12929	13510	Novosphingobium acidiphilum DSM 19966 G404DRAFT_scaffold00026.26_C, whole genome shotgun sequence	gi|523617931|gb|AUBA01000026.1|
	GST3	5.00E-34	38	55	94121	94711	Novosphingobium acidiphilum DSM 19966 G404DRAFT_scaffold00002.2_C, whole genome shotgun sequence	gi|523617981|gb|AUBA01000002.1|
	GST4	9.00E-62	46	62	15767	16471	Novosphingobium acidiphilum DSM 19966 G404DRAFT_scaffold00015.15_C, whole genome shotgun sequence	gi|523617949|gb|AUBA01000015.1|
	GST5	1.00E-114	66	77	35456	36196	Novosphingobium acidiphilum DSM 19966 G404DRAFT_scaffold00015.15_C, whole genome shotgun sequence	gi|523617949|gb|AUBA01000015.1|
	GST6	-	-	-	-	-	-	-

f

Organism	Reference enzyme	E-value	Identity %	Similarity %	Start nucleotide position	End nucleotide position	Description	Accession number
Novosphingobium sp. B-7	SDR3	2.00E-82	47	64	4707	5561	Novosphingobium sp. B-7 scaffold199_4, whole genome shotgun sequence	gi|510886133|gb|APCQ01000291.1|
		1.00E-67	42	58	5642	6502	Novosphingobium sp. B-7 scaffold199_4, whole genome shotgun sequence	gi|510886133|gb|APCQ01000291.1|
		4.00E-23	30	52	2835	3389	Novosphingobium sp. B-7 scaffold82_3, whole genome shotgun sequence	gi|510885593|gb|APCQ01000527.1|
	SDR5	1.00E-166	82	89	5573	6502	Novosphingobium sp. B-7 scaffold199_4, whole genome shotgun sequence	gi|510886133|gb|APCQ01000291.1|
		4.00E-58	40	59	4797	5561	Novosphingobium sp. B-7 scaffold199_4, whole genome shotgun sequence	gi|510886133|gb|APCQ01000291.1|

		3.00E-55	39	56	12913	13707	Novosphingobium sp. B-7 scaffold250_2, whole genome shotgun sequence	gi|510885447|gb|APCQ01000574.1|
	GST3	2.00E-37	39	54	29589	30185	Novosphingobium sp. B-7 scaffold246_2, whole genome shotgun sequence	gi|510886366|gb|APCQ01000145.1|
	GST4	1.00E-105	65	75	2367	3068	Novosphingobium sp. B-7 scaffold429_1, whole genome shotgun sequence	gi|510884874|gb|APCQ01000703.1|
		4.00E-70	50	65	429	1127	Novosphingobium sp. B-7 scaffold301_2, whole genome shotgun sequence	gi|510885331|gb|APCQ01000596.1|
	GST5	1.00E-111	64	72	1181	1933	Novosphingobium sp. B-7 scaffold190_1, whole genome shotgun sequence	gi|510886110|gb|APCQ01000314.1|
	GST6	-	-	-	-	-	-	-

g

Organism	Reference enzyme	E-value	Identity %	Similarity %	Start nucleotide position	End nucleotide position	Description	Accession number
Sphingobium xenophagum NBRC 107872	SDR3	4.00E-74	44	61	136070	136906	Sphingobium xenophagum NBRC 107872 DNA, contig: contig00601, whole genome shotgun sequence	gi|478730563|dbj|BARE01000011.1|
		8.00E-59	39	56	156034	156834	Sphingobium xenophagum NBRC 107872 DNA, contig: contig00601, whole genome shotgun sequence	gi|478730563|dbj|BARE01000011.1|
		3.00E-45	34	50	161554	162399	Sphingobium xenophagum NBRC 107872 DNA, contig: contig00301, whole genome shotgun sequence	gi|478730568|dbj|BARE01000006.1|
		2.00E-28	34	53	162438	163007	Sphingobium xenophagum NBRC 107872 DNA, contig: contig00301, whole genome shotgun sequence	gi|478730568|dbj|BARE01000006.1|
	SDR5	2.00E-85	52	66	155941	156834	Sphingobium xenophagum NBRC 107872 DNA, contig: contig00601, whole genome shotgun sequence	gi|478730563|dbj|BARE01000011.1|
		4.00E-54	42	57	136154	136906	Sphingobium xenophagum NBRC 107872 DNA, contig: contig00601, whole genome shotgun sequence	gi|478730563|dbj|BARE01000011.1|
		1.00E-46	36	51	161554	162357	Sphingobium xenophagum NBRC 107872 DNA, contig: contig00301, whole genome shotgun sequence	gi|478730568|dbj|BARE01000006.1|
	GST3	1.00E-100	70	82	168887	169501	Sphingobium xenophagum NBRC 107872 DNA, contig: contig00601, whole genome shotgun sequence	gi|478730563|dbj|BARE01000011.1|
		1.00E-38	41	55	174384	174977	Sphingobium xenophagum NBRC 107872 DNA, contig: contig00201, whole genome shotgun sequence	gi|478730572|dbj|BARE01000002.1|
	GST4	1.00E-142	84	91	169791	170522	Sphingobium xenophagum NBRC 107872 DNA, contig: contig00601, whole genome shotgun sequence	gi|478730563|dbj|BARE01000011.1|
		1.00E-55	39	56	148241	148990	Sphingobium xenophagum NBRC 107872 DNA, contig: contig00601, whole genome shotgun sequence	gi|478730563|dbj|BARE01000011.1|
	GST5	-	-	-	-	-	-	-
	GST6	1.00E-146	80	89	170588	171361	Sphingobium xenophagum NBRC 107872 DNA, contig: contig00601, whole genome shotgun sequence	gi|478730563|dbj|BARE01000011.1|

h

Organism	Reference enzyme	E-value	Identity %	Similarity %	Start nucleotide position	End nucleotide position	Description	Accession number
Sphingobium xenophagum QYY	SDR3	5.00E-75	44	61	19330	20166	Sphingobium xenophagum QYY contig058, whole genome shotgun sequence	gi|394703557|gb|AKIB01000058.1|
		1.00E-58	39	56	42865	43665	Sphingobium xenophagum QYY contig026, whole genome shotgun sequence	gi|394703625|gb|AKIB01000026.1|
		4.00E-28	34	52	45104	45673	Sphingobium xenophagum QYY contig014, whole genome shotgun sequence	gi|394703648|gb|AKIB01000014.1|
		2.00E-44	34	50	45712	46557	Sphingobium xenophagum QYY contig014, whole genome shotgun sequence	gi|394703648|gb|AKIB01000014.1|
	SDR5	8.00E-87	52	66	42865	43758	Sphingobium xenophagum QYY contig026, whole genome shotgun sequence	gi|394703625|gb|AKIB01000026.1|
		4.00E-55	42	57	19414	20166	Sphingobium xenophagum QYY contig058, whole genome shotgun sequence	gi|394703557|gb|AKIB01000058.1|
		6.00E-45	35	50	45754	46557	Sphingobium xenophagum QYY contig014, whole genome shotgun sequence	gi|394703648|gb|AKIB01000014.1|
	GST3	1.00E-102	71	82	30197	30811	Sphingobium xenophagum QYY contig026, whole genome shotgun sequence	gi|394703625|gb|AKIB01000026.1|
		3.00E-39	41	55	40752	41345	Sphingobium xenophagum QYY contig104, whole genome shotgun sequence	gi|394703460|gb|AKIB01000104.1|
	GST4	1.00E-142	84	91	29176	29907	Sphingobium xenophagum QYY contig026, whole genome shotgun sequence	gi|394703625|gb|AKIB01000026.1|
		2.00E-56	39	56	52018	52767	Sphingobium xenophagum QYY contig026, whole genome shotgun sequence	gi|394703625|gb|AKIB01000026.1|
	GST5	-	-	-	-	-	-	-
	GST6	1.00E-145	79	87	28337	29110	Sphingobium xenophagum QYY contig026, whole genome shotgun sequence	gi|394703625|gb|AKIB01000026.1|

I

Organism	Reference enzyme	E-value	Identity %	Similarity %	Start nucleotide position	End nucleotide position	Description	Accession number
Sphingomonas wittichii DP58	SDR3	2.00E-56	40	56	10050	10868	Sphingomonas wittichii DP58 contig000142, whole genome shotgun sequence	gi|375076127|gb|AHKO01000141.1|
		9.00E-29	36	53	13298	13864	Sphingomonas wittichii DP58 contig000592, whole genome shotgun sequence	gi|375075677|gb|AHKO01000591.1|
		6.00E-25	35	52	15110	15679	Sphingomonas wittichii DP58 contig000144, whole genome shotgun sequence	gi|375076125|gb|AHKO01000143.1|
		3.00E-28	34	51	16302	16889	Sphingomonas wittichii DP58 contig000153, whole genome shotgun sequence	gi|375076116|gb|AHKO01000152.1|
		3.00E-25	33	50	6855	7415	Sphingomonas wittichii DP58 contig000681, whole genome shotgun sequence	gi|375075588|gb|AHKO01000680.1|
		1.00E-25	33	51	6427	7005	Sphingomonas wittichii DP58 contig000454, whole genome shotgun sequence	gi|375075815|gb|AHKO01000453.1|
		3.00E-26	32	51	7855	8454	Sphingomonas wittichii DP58 contig000592, whole genome shotgun sequence	gi|375075677|gb|AHKO01000591.1|
		8.00E-25	31	50	24320	24886	Sphingomonas wittichii DP58 contig000497, whole genome shotgun sequence	gi|375075772|gb|AHKO01000496.1|
		6.00E-21	28	50	10335	10895	Sphingomonas wittichii DP58 contig000404, whole genome shotgun sequence	gi|375075865|gb|AHKO01000403.1|
		1.00E-30	28	50	18969	19556	Sphingomonas wittichii DP58 contig000235, whole genome shotgun sequence	gi|375076034|gb|AHKO01000234.1|
		2.00E-21	28	50	12802	13341	Sphingomonas wittichii DP58 contig000623, whole genome shotgun sequence	gi|375075646|gb|AHKO01000622.1|
	SDR5	1.00E-27	38	50	4043	4603	Sphingomonas wittichii DP58 contig000456, whole genome shotgun sequence	gi|375075813|gb|AHKO01000455.1|
		4.00E-45	38	51	10077	10862	Sphingomonas wittichii DP58 contig000142, whole genome shotgun sequence	gi|375076127|gb|AHKO01000141.1|
		9.00E-28	37	50	13268	13864	Sphingomonas wittichii DP58 contig000592, whole genome shotgun sequence	gi|375075677|gb|AHKO01000591.1|
		1.00E-28	34	51	27331	27897	Sphingomonas wittichii DP58 contig000456, whole genome shotgun sequence	gi|375075813|gb|AHKO01000455.1|
		3.00E-23	31	52	1603	2181	Sphingomonas wittichii DP58 contig000206, whole genome shotgun sequence	gi|375076063|gb|AHKO01000205.1|
		6.00E-19	27	50	2956	3543	Sphingomonas wittichii DP58 contig000581, whole genome shotgun sequence	gi|375075688|gb|AHKO01000580.1|
	GST3	6.00E-47	45	60	4974	5510	Sphingomonas wittichii DP58 contig000553, whole genome shotgun sequence	gi|375075716|gb|AHKO01000552.1|
		1.00E-55	45	64	265	831	Sphingomonas wittichii DP58 contig000196, whole genome shotgun sequence	gi|375076073|gb|AHKO01000195.1|
		2.00E-39	40	58	5426	6013	Sphingomonas wittichii DP58 contig000288, whole genome shotgun sequence	gi|375075981|gb|AHKO01000287.1|
		1.00E-37	40	54	4390	4947	Sphingomonas wittichii DP58 contig000027, whole genome shotgun sequence	gi|375076241|gb|AHKO01000027.1|
		5.00E-35	39	57	8	547	Sphingomonas wittichii DP58 contig000565, whole genome shotgun sequence	gi|375075704|gb|AHKO01000564.1|
		4.00E-25	29	50	4465	5001	Sphingomonas wittichii DP58 contig000031, whole genome shotgun sequence	gi|375076237|gb|AHKO01000031.1|
	GST4	-	-	-	-	-	-	-
	GST5	8.00E-54	38	55	134	892	Sphingomonas wittichii DP58 contig000567, whole genome shotgun sequence	gi|375075702|gb|AHKO01000566.1|
	GST6	-	-	-	-	-	-	-

Similarity between GGGE-converting enzymes from strain MBES04 and homologous proteins of selected species and their chromosomal locations or available contigs are shown.
-; Not detected

Table S6. Differentially expressed genes in response to GGGE and MPHPV in whole-genome transcriptional profiling

a Upregurated genes in response to GGGE

Gene ID	Putative function	Fold change	a.value[1]	m.value[2]	p.value	q.value	KO entry[3]	KEGG pathway or Definition
GAM03020	hypothetical protein	2.0	9.3E+00	1.0E+00	9.4E-07	3.0E-04		-[4]
GAM03021	outer membrane protein	2.8	1.3E+01	1.5E+00	5.5E-15	1.3E-11	K18139	β-lactam resistance
GAM03022	hydrophobe/amphiphile efflux-1 (HAE1) family transporter	2.7	1.4E+01	1.4E+00	9.9E-14	1.5E-10	K03296	hydrophobic/amphiphilic exporter-1 (mainly G-bacteria), HAE1 family
GAM03023	membrane fusion protein	2.6	1.2E+01	1.4E+00	3.3E-13	3.1E-10	K03585	β-lactam resistance
GAM03024	TetR family transcriptional regulator	1.8	1.0E+01	8.8E-01	9.0E-06	2.2E-03		-
GAM03455	hypothetical protein	6.0	7.4E+00	2.6E+00	7.6E-24	3.6E-20		-
GAM03456	*p*-cresol methylhydroxylase subunit	2.9	9.0E+00	1.5E+00	4.6E-13	3.6E-10	K05797	toluene degradation
GAM03457	*p*-cresol methylhydroxylase subunit	2.6	6.4E+00	1.4E+00	3.8E-06	1.1E-03		toluene degradation
GAM03580	cation efflux protein	3.6	4.8E+00	1.9E+00	4.3E-05	7.4E-03		-
GAM03631	acyl-CoA dehydrogenase	1.8	1.1E+01	8.3E-01	1.9E-05	4.0E-03		-
GAM03632	3-hydroxyacyl-CoA dehydrogenase	1.9	1.2E+01	9.3E-01	1.3E-06	3.8E-04	K07516	fatty acid degradation/carbon fixation pathways in prokaryotes/carbon metabolism/fatty acid metabolism
GAM03633	acetyl-CoA acyltransferase	1.7	1.1E+01	8.1E-01	3.1E-05	5.9E-03	K00632	fatty acid degradation/valine, leucine and isoleucine degradation/geraniol degradation (metabolism of terpenoids and polyketides)/benzoate degradation/a-linolenic acid metabolism/ethylbenzene degradation/fatty acid metabolism
GAM03896	calcium-binding protein	2.3	8.6E+00	1.2E+00	3.0E-08	1.2E-05		-
GAM04082	hypothetical conserved protein	2.5	8.4E+00	1.3E+00	3.4E-09	1.6E-06		-
GAM04083	hypothetical protein	2.6	8.9E+00	1.4E+00	4.4E-11	2.6E-08		-
GAM04124	2-keto-4-pentenoate hydratase	1.7	9.9E+00	7.3E-01	2.5E-04	3.7E-02	K02554	phenylalanine metabolism/benzoate degradation/dioxin degradation/xylene degradation/degradation of aromatic compounds
GAM04237	Malate:quinone oxidoreductase	2.0	8.0E+00	1.0E+00	1.3E-05	2.8E-03	K00116	citrate cycle/pyruvate metabolism/carbon metabolism
GAM04562	PadR family transcriptional regulator	1.7	1.2E+01	7.8E-01	3.6E-05	6.5E-03	K10947	PadR family transcriptional regulator, regulatory protein PadR
GAM04568	hypothetical conserved protein	1.8	9.0E+00	8.5E-01	5.1E-05	8.6E-03		-
GAM04702	hypothetical protein	1.6	9.6E+00	7.2E-01	3.5E-04	5.0E-02		-
GAM04949	two component LuxR family transcriptional regulator	1.9	7.2E+00	9.5E-01	2.1E-04	3.3E-02		-
GAM05027	FAD dependent oxidoreductase	2.1	8.4E+00	1.1E+00	7.7E-07	2.6E-04	K00111	glycerophospholipid metabolism
GAM05028	major facilitator superfamily glycerol uptake transporter	2.2	8.2E+00	1.1E+00	5.8E-07	2.1E-04	K02440	glycerol uptake facilitator protein
GAM05030	glycerol kinase	1.8	8.9E+00	8.8E-01	3.1E-05	5.9E-03	K00864	glycerolipid metabolism/PPAR signaling pathway/plant-pathogen interaction
GAM05137	CopG family transcriptional regulator	1.7	9.3E+00	7.4E-01	2.7E-04	4.0E-02	K07722	CopG family transcriptional regulator, nickel-responsive regulator
GAM06305	hypothetical conserved protein	2.6	8.8E+00	1.4E+00	1.9E-10	1.0E-07		-
GAM06306	neutral amino acid transporter B(0)-like	2.7	9.7E+00	1.4E+00	1.2E-12	8.4E-10	K11103	two-component system
GAM06750	REDY-like protein HapK	1.9	7.5E+00	9.3E-01	1.4E-04	2.3E-02		-

b Upregurated genes in response to MPHPV

Gene ID	Putative function	Fold change	a.value	m.value	p.value	q.value	KO entry	KEGG pathway or Definition
GAM03020	hypothetical protein	2.1	9.3E+00	1.1E+00	1.1E-06	2.1E-04		-
GAM03021	outer membrane protein	2.9	1.3E+01	1.5E+00	6.3E-14	3.3E-11	K18139	β-lactam resistance
GAM03022	hydrophobe/amphiphile efflux-1 (HAE1) family transporter	3.1	1.4E+01	1.6E+00	2.9E-15	4.4E-12	K03296	hydrophobic/amphiphilic exporter-1 (mainly G-bacteria), HAE1 family
GAM03023	membrane fusion protein	3.1	1.2E+01	1.6E+00	3.9E-15	4.4E-12	K03585	β-lactam resistance
GAM03024	TetR family transcriptional regulator	2.1	1.0E+01	1.1E+00	3.7E-07	7.5E-05		-
GAM03347	50S ribosomal protein L34	3.0	1.2E+01	1.6E+00	1.9E-14	1.3E-11	-	-
GAM03412	flagellar FliL protein	2.0	6.9E+00	9.7E-01	5.3E-04	4.1E-02	-	flagellar FliL protein
GAM03455	hypothetical protein	2.3	6.7E+00	1.2E+00	2.8E-05	3.8E-03	-	-
GAM03551	hypothetical protein	4.6	4.1E+00	2.2E+00	5.2E-05	6.1E-03	-	-
GAM03663	RND efflux system	3.2	6.9E+00	1.7E+00	3.6E-09	1.3E-06	K18139	β-lactam resistance
GAM03664	RND superfamily multidrug efflux pump acriflavin resistance protein	2.4	8.4E+00	1.3E+00	5.4E-08	1.3E-05	K18138	β-lactam resistance
GAM03665	multidrug resistance protein MexA	2.8	7.1E+00	1.5E+00	4.1E-08	1.1E-05	K03585	β-lactam resistance
GAM03825	hypothetical protein	2.7	9.1E+00	1.4E+00	2.0E-10	9.2E-08		-
GAM04021	hypothetical conserved protein	4.9	5.2E+00	2.3E+00	2.8E-08	7.7E-06	-	-
GAM04288	Rieske (2Fe-2S) domain-containing protein	1.8	7.6E+00	8.6E-01	5.9E-04	4.2E-02	-	-
GAM04293	acyl-CoA dehydrogenase 6-like	2.4	5.9E+00	1.3E+00	1.5E-04	1.5E-02	K00257	geraniol degradation
GAM04295	acyl-CoA dehydrogenase	2.3	5.8E+00	1.2E+00	5.6E-04	4.2E-02	K00257	geraniol degradation
GAM04307	hypothetical protein	8.9	7.7E+00	3.2E+00	7.4E-33	3.5E-29	-	-
GAM04315	Transcriptional regulator kdgR	2.0	8.5E+00	1.0E+00	6.7E-06	1.1E-03	-	-
GAM04323	acyl-CoA dehydrogenase	1.8	8.4E+00	8.2E-01	3.6E-04	2.9E-02	-	-
GAM04591	membrane protein	1.8	1.1E+01	8.8E-01	1.8E-05	2.6E-03	K08973	putative membrane protein
GAM04844	hypothetical conserved protein	2.0	7.0E+00	9.9E-01	3.4E-04	2.8E-02		-
GAM04943	aldehyde dehydrogenase	3.5	8.8E+00	1.8E+00	4.7E-15	4.4E-12	K00138	glycolysis-gluconeogenesis/pyruvate metabolism
GAM04949	two component LuxR family transcriptional regulator	2.2	7.3E+00	1.2E+00	1.1E-05	1.6E-03	-	-
GAM04950	pyrrolo-quinoline quinone	2.9	8.1E+00	1.5E+00	2.3E-10	9.7E-08	K00114	glycolysis-gluconeogenesis/chloroalkane and chloroalkene degradation/propanoate metabolism

GAM04951	extracellular solute-binding protein	2.4	6.7E+00	1.3E+00	7.4E-06	1.2E-03	-	-
GAM04952	cytochrome C550	3.2	6.6E+00	1.7E+00	1.5E-08	4.6E-06	-	-
GAM05027	FAD dependent oxidoreductase	2.3	8.4E+00	1.2E+00	3.2E-07	6.9E-05	K00111	glycerophospholipid metabolism
GAM05028	major facilitator superfamily glycerol uptake transporter	2.3	8.3E+00	1.2E+00	2.0E-07	4.5E-05	K02440	glycerol uptake facilitator protein
GAM05029	hypothetical protein	1.8	8.1E+00	8.8E-01	2.0E-04	1.9E-02	-	
GAM05030	glycerol kinase	1.9	8.9E+00	9.4E-01	2.5E-05	3.4E-03	K00864	glycerolipid metabolism/PPAR signaling pathway/plant-pathogen interaction
GAM05489	hypothetical conserved protein	2.0	7.6E+00	1.0E+00	6.3E-05	7.0E-03	-	-
GAM05496	hypothetical conserved protein	2.0	6.5E+00	1.0E+00	5.9E-04	4.2E-02	-	-
GAM05498	hypothetical conserved protein	2.4	6.1E+00	1.3E+00	6.5E-05	7.1E-03	-	-
GAM05503	carotenoid oxygenase	1.7	9.6E+00	7.5E-01	4.7E-04	3.7E-02	-	-
GAM05551	hypothetical protein	1.7	8.6E+00	7.8E-01	5.5E-04	4.2E-02	-	-
GAM05659	hypothetical protein	4.1	7.4E+00	2.1E+00	1.3E-14	1.0E-11	-	-
GAM05660	multidrug resistance efflux pump	2.5	1.0E+01	1.3E+00	4.7E-10	1.8E-07	K03543	multidrug resistance protein A
GAM05661	EmrB/QacA family drug resistance transporter	3.5	9.8E+00	1.8E+00	9.8E-17	2.3E-13	K03446	MFS transporter, DHA2 family, multidrug resistance protein B
GAM05662	outer membrane protein	3.1	9.9E+00	1.6E+00	2.2E-14	1.3E-11	-	-
GAM05838	TetR family transcriptional regulator	1.8	9.9E+00	8.5E-01	5.4E-05	6.2E-03	-	-
GAM05839	RND efflux system outer membrane lipoprotein	1.7	1.1E+01	7.7E-01	1.8E-04	1.7E-02	-	-
GAM05841	MFS transporter DHA2 family multidrug resistance protein B	1.8	1.1E+01	8.5E-01	3.7E-05	4.6E-03	K03446	MFS transporter, DHA2 family, multidrug resistance protein B
GAM05995	hypothetical conserved protein	2.2	6.9E+00	1.2E+00	3.7E-05	4.6E-03		-
GAM06021	50S ribosomal protein L36	1.9	1.1E+01	9.2E-01	7.5E-06	1.2E-03	K02919	ribosome
GAM06195	Flp/Fap pilin component family protein	1.6	1.3E+01	7.1E-01	4.4E-04	3.6E-02	-	-
GAM06305	hypothetical conserved protein	2.0	8.6E+00	1.0E+00	5.6E-06	9.7E-04	-	-
GAM06306	neutral amino acid transporter B(0)-like	2.1	9.5E+00	1.1E+00	5.1E-07	1.0E-04	K11103	two-component system
GAM07274	hypothetical conserved protein	1.7	1.3E+01	7.3E-01	2.7E-04	2.4E-02	-	-
GAM07297	hypothetical conserved protein	1.9	8.5E+00	9.5E-01	3.4E-05	4.4E-03	-	-
GAM07522	macrolide-specific ABC-type efflux carrier	1.8	8.5E+00	8.2E-01	3.3E-04	2.8E-02	K05685	ABC transporters

c Downregurated genes in response to GGGE

Gene ID	Putative function	Fold change	a.value	m.value	p.value	q.value	KO entry	KEGG pathway or Definition
GAM03320	30S ribosomal protein S20	0.6	1.5E+01	-8.2E-01	1.3E-05	2.8E-03	K02968	ribosome
GAM03671	benzaldehyde dehydrogenase II	0.6	9.9E+00	-8.5E-01	1.9E-05	4.0E-03	K00128	multiple pathways[(5)]
GAM03672	4-nitrobenzyl alcohol dehydrogenase NtnD	0.6	1.1E+01	-8.6E-01	8.2E-06	2.1E-03	K00119	phosphonate and phosphinate metabolism
GAM03825	hypothetical protein	0.3	7.5E+00	-1.8E+00	1.4E-13	1.7E-10	-	-
GAM05610	hypothetical conserved protein	0.4	8.0E+00	-1.3E+00	1.1E-08	4.7E-06	-	-

d Downregurated genes in response to MPHPV

Gene ID	Putative function	Fold change	a.value	m.value	p.value	q.value	KO entry	KEGG pathway or Definition
GAM03046	RND family efflux transporter MFP subunit	0.5	8.3E+00	-1.1E+00	2.2E-06	3.9E-04	K03585	β-lactam resistance
GAM03047	acriflavin resistance protein	0.5	9.7E+00	-9.3E-01	1.2E-05	1.8E-03	K18307	multidrug efflux pump
GAM03048	RND efflux system outer membrane lipoprotein	0.4	9.1E+00	-1.2E+00	2.0E-08	5.9E-06	-	-
GAM03180	catalase/peroxidase HPI	0.4	1.2E+01	-1.2E+00	5.9E-09	2.0E-06	K03782	phenylalanine metabolism/tryptophan metabolism/phenylpropanoid biosynthesis
GAM03672	4-nitrobenzyl alcohol dehydrogenase NtnD	0.6	1.1E+01	-8.1E-01	7.9E-05	8.4E-03	K00119	phosphonate and phosphinate metabolism
GAM04427	cb-type cytochrome c oxidase subunit III	0.6	1.0E+01	-7.6E-01	2.8E-04	2.4E-02	K00406	oxidative phosphorylation/two-component system
GAM04428	cb-type cytochrome c oxidase subunit IV	0.5	7.8E+00	-9.5E-01	1.0E-04	1.1E-02	K00407	oxidative phosphorylation/two-component system
GAM04429	cb-type cytochrome c oxidase subunit II	0.6	1.0E+01	-7.9E-01	1.4E-04	1.5E-02	K00405	oxidative phosphorylation/two-component system
GAM04430	cb-type cytochrome c oxidase subunit I	0.6	1.2E+01	-6.9E-01	5.9E-04	4.2E-02	K00404	oxidative phosphorylation/two-component system
GAM04896	membrane protein	0.4	8.6E+00	-1.2E+00	1.9E-07	4.5E-05	-	-
GAM05017	ornithine cyclodeaminase	0.5	7.8E+00	-9.2E-01	1.6E-04	1.6E-02	K01750	arginine and proline metabolism/biosynthesis of amino acids
GAM05051	phage shock protein A, PspA	0.6	9.7E+00	-7.7E-01	2.6E-04	2.4E-02	K03969	phage shock protein A
GAM05090	hypothetical protein	0.5	8.2E+00	-8.7E-01	2.3E-04	2.1E-02	-	-
GAM05231	hypothetical protein	0.5	6.2E+00	-1.1E+00	5.1E-04	4.0E-02	-	-
GAM05679	hypothetical protein	0.5	8.4E+00	-9.4E-01	4.7E-05	5.7E-03		-

[(1)(2)] The a- and m-values were calculated as follows:
a-value = $1/2\log_2(G1G2) = \log_2(G1)+\log_2(G2)$
m-value = $\log_2(G2/G1) = \log_2(G2)-\log_2(G1)$
G1: gene expression level in the control condition
G2: gene expression level in response to GGGE or MPHPV
Genes differentially expressed in response specifically to GGGE or MPHPV are highlighted in gray.

[(3)] KO entries are the defined ortholog groups categorized under the hierarchy of KEGG pathways and BRITE ontologies in Kyoto Encyclopedia of Genes and Genomes Databases (KEGG) (http://www.genome.jp/kegg/).

[(4)] Not defined due to the low nucleotide sequence similarity to the KO entries

[(5)] KEGG pathways involving K00128

glycolysis / gluconeogenesis	lysine degradation	glycerolipid metabolism
pentose and glucuronate interconversions	arginine and proline metabolism	pyruvate metabolism
ascorbate and aldarate metabolism	histidine metabolism	chloroalkane and chloroalkene degradation
fatty acid degradation	tryptophan metabolism	propanoate metabolism
valine, leucine and isoleucine degradation	β-alanine metabolism	limonene and pinene degradation

Table S7. Primers used for qRT-PCR

Target	Primer name	Sequence	Amplicon size (bp)
GAM05523	g0069_fw	GCGCATCTCAAGAACGTG	132
	g0069_rv	GATGTCTTCCAGCGGCTT	
GAM05529	g0075_fw	GACGCGAACTACACGTTG	114
	g0075_rv	GACCTCATGGTCGATCAGTG	
GAM05530	g0075_fw	AGGTTCTGGACGAGGAAATG	125
	g0075_rv	GATGGCGAAGTTGCAGATG	
GAM05531	g0077_fw	CCGAATACCTCGATGAGACT	156
	g0077_rv	GCAACGACAGATCATGGTAG	
GAM05532	g0078_fw	GCTATC GCATGATCCTGAAC	135
	g0078_rv	CGAAACGGTCGAACAGGTA	
GAM05547	g0093_fw	GTTTCTTCCACCTCTACCAGAC	89
	g0093_rv	GTGGGTGAGGATGTAGAGTTC	
16SrDNA	16Sr_fw	TGGGCACTCTAAGGAAACTG	109
	16Sr_rv	GTCACCGCCATTGTAGCA	

IV. Supplementary references

1. Addison, S.L., Foote, S.M., Reid, N.M, & Lloyd-Jones, G. *Novosphingobium nitrogenifigens* sp. nov., a polyhydroxyalkanoate-accumulating diazotroph isolated from a New Zealand pulp and paper wastewater. *Int. J. Syst. Evol. Microbiol.* **57**, 2467-2471 (2007).
2. Adriaenssens, E.M., Guerrero, L.D., Makhalanyane, T.P., Aislabie, J.M. & Cowan, D.A. Draft genome sequence of the aromatic hydrocarbon-degrading bacterium *Sphingobium* sp. strain Ant17, isolated from Antarctic soil. *Genome Announc.* **2,** e00212-14 (2014).
3. Anand, S.*, et al.* Genome sequence of *Sphingobium indicum* B90A, a hexachlorocyclohexane-degrading bacterium. *J. Bacteriol.* **194**, 4471-4472 (2012).
4. Asker. D., Beppu, T. & Ueda, K. *Sphingomonas jaspsi* sp. nov., a novel carotenoid-producing bacterium isolated from Misasa, Tottori, Japan. *Int. J. Syst. Evol. Microbiol.* **57**, 1435-1441 (2007).
5. Aylward, F.O.*, et al.* Comparison of 26 Sphingomonad genomes reveals diverse environmental adaptations and biodegradative capabilities. *Appl. Environ. Microbiol.* **79**, 3724-3733 (2013).
6. Brown, S.D.*, et al.* Twenty-one genome sequences from *Pseudomonas* species and 19 genome sequences from diverse bacteria isolated from the rhizosphere and endosphere of *Populus deltoides*. *J. Bacteriol.* **194**, 5991-5993 (2012).
7. Buonaurio, R.*, et al. Sphingomonas melonis* sp nov., a novel pathogen that causes brown spots on yellow Spanish melon fruits. *Int. J. Syst. Evol. Microbiol.* **52**, 2081-2087 (2002).
8. Chen, Y.*, et al.* Kraft lignin biodegradation by *Novosphingobium* sp B-7 and analysis of the degradation process. *Bioresour. Technol.* **123**, 682-685 (2012).
9. Copley, S.D.*, et al.* The whole genome sequence of *Sphingobium chlorophenolicum* L-1:

Insights into the evolution of the pentachlorophenol degradation pathway. *Genome Biol. Evol.* **4**, 184-198 (2012).

10. Cua, L.S. & Stein, L.Y. Characterization of denitrifying activity by the alphaproteobacterium, *Sphingomonas wittichii* RW1. *Front Microbiol.* **5**, 404-404 (2014).
11. D'Argenio, V., *et al. De Novo* sequencing and assembly of the whole genome of *Novosphingobium* sp. strain PP1Y. *J. Bacteriol.* **193**, 4296-4296 (2011).
12. Desiniotis, A., *et al.* Complete genome sequence of the ethanol-producing *Zymomonas mobilis* subsp *mobilis* centrotype ATCC 29191. *J. Bacteriol.* **194**, 5966-5967 (2012).
13. Dong, C., *et al.* Draft genome sequence of *Sphingobium* sp. strain C100, a polycyclic aromatic hydrocarbon-degrading bacterium from the deep-sea sediment of the Arctic ocean. *Genome Announc.* **2**, e01210-13 (2014).
14. Eugenia-Farias, M., *et al.* Genome sequence of *Sphingomonas* sp. S17, isolated from an alkaline, hyperarsenic, and hypersaline volcano-associated lake at high altitude in the Argentinean puna. *J. Bacteriol.* **193**, 3686-3687 (2011).
15. Fida, T.T., Moreno-Forero, S.K., Heipieper, H.J. & Springael, D. Physiology and transcriptome of the polycyclic aromatic hydrocarbon-degrading *Sphingomonas* sp. LH128 after long-term starvation. *Microbiology.* **159**, 1807-1817 (2013).
16. Fredrickson, J.K., Brockman, F.J., Workman, D.J., Li, S.W. & Stevens, T.O. Isolation and characterization of a subsurface bacterium capable of growth on toluene, naphthalene, and other aromatic compounds. *Appl. Environ. Microbiol.* **57**, 796-803 (1991)
17. Gai, Z., *et al.* Genome sequence of *Sphingobium yanoikuyae* XLDN2-5, an efficient carbazole-degrading strain. *J. Bacteriol.* **193**, 6404-6405 (2011).
18. Gai, Z., *et al.* Genome sequence of *Sphingomonas elodea* ATCC 31461, a highly productive industrial strain of gellan gum. *J. Bacteriol.* **193**, 7015-7016 (2011).
19. Gan, H.M., Buckley, L., Szegedi, E., Hudson, A.O. & Savka, M.A. Identification of an *rsh*

gene from a *Novosphingobium* sp. necessary for quorum-sensing signal accumulation. *J. Bacteriol.* **191**, 2551-2560 (2009).

20. Gan, H.M., Chew, T.H., Hudson, A.O. & Savka, M.A. Genome Sequence of *Novosphingobium* sp. strain Rr 2-17, a Nopaline crown gall-associated bacterium isolated from *Vitis vinifera* L. Grapevine. *J. Bacteriol.* **194**, 5137-5138 (2012).
21. Gan, H.Y., *et al.* Whole-genome sequences of 13 endophytic bacteria isolated from shrub willow (salix) grown in geneva, new york. *Genome Announc* **2**, e00288-14 (2014).
22. Gich, F. & Overmann, J. *Sandarakinorhabdus limnophila* gen. nov., sp nov., a novel bacteriochlorophyll a-containing, obligately aerobic bacterium isolated from freshwater lakes. *Int. J. Syst. Evol. Microbiol.* **56**, 847-854 (2006).
23. Glaeser, S.P., Kaempfer, P., Busse. H.J., Langer, S. & Glaeser, J. *Novosphingobium acidiphilum* sp. nov., an acidophilic salt-sensitive bacterium isolated from the humic acid-rich Lake Grosse Fuchskuhle. *Int. J. Syst. Evol. Microbiol.* **59**, 323-330 (2009).
24. Herman, R.A., Phillips, A.M., Lepping, M.D., Fast, B.J. & Sabbatini, J. Compositional safety of event DAS-40278-9 (AAD-1) herbicide-tolerant maize. *GM crops.* **1**, 294-311(2010).
25. Hoffmann, J., Bona-Lovasz, J., Beuttler, H. & Altenbuchner, J. *In vivo* and *in vitro* studies on the carotenoid cleavage oxygenases from *Sphingopyxis alaskensis* RB2256 and *Plesiocystis pacifica* SIR-1 revealed their substrate specificities and non-retinal-forming cleavage activities. *FEBS J.* **279**, 3911-3924 (2012).
26. Hu, A.Y., He, J.B. Chu, K.H. &Yu, C.P. Genome sequence of the 17β-estradiol-utilizing bacterium *Sphingomonas* strain KC8. *J. Bacteriol.* **193**, 4266-4267 (2011).
27. Hu, A., Lv, M. &Yu, C.P. Draft genome sequence of the bisphenol A-degrading bacterium *Sphingobium* sp. strain YL23. *Genome Announc.* **1**, e00549-13 (2013).
28. Huang HD, *et al. Sphingomonas sanxanigenens* sp. nov., isolated from soil. *Int. J. Syst.*

Evol. Microbiol. **59**, 719-723 (2009).

29. Innerebner, G., Knief, C. & Vorholt JAProtection of *Arabidopsis thaliana* against leaf-pathogenic *Pseudomonas syringae* by *Sphingomonas* strains in a controlled model system. *Appl. Environ. Microbiol.* **77**, 3202-3210 (2011).
30. Jiao. N.Z., Zhang, R. & Zheng, Q. Coexistence of two different photosynthetic operons in *Citromicrobium bathyomarinum* JL354 as revealed by whole-genome sequencing. *J. Bacteriol.* **192**, 1169-1170 (2010).
31. Kaur, J., Verma, H., Tripathi,C., Khurana, J.P. & Lal, R. Draft genome sequence of a hexachlorocyclohexane-degrading bacterium, *Sphingobium baderi* strain LL03^{T}. *Genome Announc.* **1**, e00751-13 (2013).
32. Kohli, P., *et al.* Draft genome sequence of *Sphingobium ummariense* strain RL-3, a hexachlorocyclohexane-degrading bacterium. *Genome Announc.* **1**, e00956-13 (2013).
33. Kouveli, V.N., *et al.* Genome sequence of the ethanol-producing *Zymomonas mobilis* subsp. *pomaceae* lectotype strain ATCC 29192. *J. Bacteriol.* **193**, 5049-5050 (2011).
34. Kouvelis, V.N., *et al.* Complete genome sequence of the ethanol producer *Zymomonas mobilis* NCIMB 11163. *J. Bacteriol.* **191**, 7140-7141(2009).
35. Kouvelis, V.N., *et al.* Finished genome of *Zymomonas mobilis* subsp. *mobilis* strain CP4, an applied ethanol producer. *Genome Announc.* **2,** e00845-13 (2014).
36. Kumar. S.A., *et al.* Draft genome sequence of *Sphingobium quisquiliarum* strain P25^{T}, a novel hexachlorocyclohexane (HCH)-degrading bacterium Isolated from an HCH dumpsite. *Genome Announc.* **1**, e00717-13 (2013).
37. Kumar, R., Dwivedi, V., Negi, V., Khurana, J.P. & Lal, R. Draft genome sequence of Sphingobium lactosutens strain DS20^{T}, isolated from a hexachlorocyclohexane dumpsite. *Genome Announc.* **1**, (2013).
38. Kunihiro, M., Ozeki, Y., Nogi, Y., Hamamura, N. & Kanaly, R.A. Benz[*a*]anthracene

biotransformation and production of ring fission products by *Sphingobium* sp. strain KK22. *Appl. Environ. Microbiol.* **79**, 4410-4420 (2013).

39. Lal, R., *et al.* Biochemistry of microbial degradation of hexachlorocyclohexane and prospects for bioremediation. *Microbiol. Mol. Biol. Rev.* **74**, 58-80(2010).
40. Lee, H., *et al.* Genome squence of *Sphingomonas* sp. strain PAMC 26621, an Arctic-lichen-associated bacterium isolated from a *Cetraria* sp. *J. Bacteriol.* **194**, 3030-3030 (2012).
41. Lee, J., *et al.* Draft genome sequence of a *Sphingomonas* sp., an endosymbiotic bacterium isolated from an Arctic lichen *Umbilicaria* sp. *J. Bacteriol*.**194**, 3010-3011 (2012).
42. Lima, A.R.J., *et al.* Draft genome sequence of *Blastomonas* sp. strain CACIA 14H2, a heterotrophic bacterium associated with Cyanobacteria. *Genome Announc.* **2**, e01200-13 (2014).
43. Lyu, Y., Zheng, W., Zheng, T. & Tian, Y. Biodegradation of polycyclic aromatic hydrocarbons by *Novosphingobium pentaromativorans* US6-1. *Plos One.* 9, e101438 (2014).
44. Ma Z, *et al.* Genome sequence of *Sphingomonas wittichii* DP58, the first reported phenazine-1-carboxylic acid-degrading strain. *J. Bacteriol*.**194**, 3535-3536 (2012).
45. Masai, E., Katayama, Y. & Fukuda, M. Genetic and biochemical investigations on bacterial catabolic pathways for lignin-derived aromatic compounds. *Biosci. Biotechnol. Biochem.* **71**, 1-15 (2007).
46. Mukherjee, U., Kumar, R., Mahato, N.K., Khurana, J.P. & Lal, R. Draft genome sequence of *Sphingobium* sp. strain HDIPO4, an avid degrader of hexachlorocyclohexane. *Genome Announc*. **1**, e00749-13 (2013).
47. Nagata, Y., *et al.* Complete genome sequence of the representative γ-hexachlorocyclohexane-degrading bacterium *Sphingobium japonicum* UT26. *J. Bacteriol.*

192, 5852-5853 (2010).

48. Nandy, S., Dudeja, M., Das, A.K. & Tiwari, R. Community acquired bacteremia by *Sphingomonas paucimobilis*: two rare case reports. *J. Clin. Diagn. Res*, **7**, 2947-2949 (2013).
49. Negi, V., *et al.* Draft genome sequence of hexachlorohexane (HCH)-degrading *Sphingobium lucknowense* strain F2^T, isolated from an HCH dumpsite. *Genome Announc.* **2**, e00788-14 (2014).
50. Niharika, N., *et al.* Draft genome sequence of *Sphingobium chinhatense* strain IP26^T, isolated from a Hexachlorocyclohexane dumpsite. *Genome Announc.* **1**, e00680-13 (2013).
51. Pal, R., Bhasin, V.K. & Lal, R. Proposal to reclassify *Sphingomonas xenophaga* Stolz *et al*. 2000 and *Sphingomonas taejonensis* Lee *et al*. 2001 as *Sphingobium xenophagum* comb. nov and *Sphingopyxis taejonensis* comb. nov., respectively. *Int. J. Syst. Evol. Microbiol.* **56**, 667-670 (2006).
52. Pappas, K.M., *et al.* Genome sequence of the ethanol-producing *Zymomonas mobilis* subsp. *mobilis* lectotype strain ATCC 10988. *J. Bacteriol.* **193,** 5051-5052 (2011).
53. Qu, Y., *et al.* Genome sequence of *Sphingomonas xenophaga* QYY, an anthraquinone-degrading strain. *Genome Announc.* **1**, e00031-12 (2013).
54. Rivas, R., Abril, A., Trujillo, M.E. &Velazquez, E. *Sphingomonas phyllosphaerae* sp. nov., from the phyllosphere of *Acacia caven* in Argentina. *Int. J Syst. Evol. Microbiol.* **54**, 2147-2150 (2004).
55. Romine, M.F., *et al.* Complete sequence of a 184-kilobase catabolic plasmid from *Sphingomonas aromaticivorans* F199. *J. Bacteriol.* **181**, 1585-1602 (1999).
56. Saxena, A., *et al.* Genome sequence of *Novosphingobium lindaniclasticum* LE124^T, isolated from a hexachlorocyclohexane dumpsite. *Genome Announc.* **1**, e00715-13 (2013).
57. Seo, J.S., *et al.* (2005) The genome sequence of the ethanologenic bacterium *Zymomonas*

mobilis ZM4. *Nat. Biotechnol.* **23**, 63-68.

58. Shin, S.C., *et al.* Genome sequence of *Sphingomonas* sp. strain PAMC 26605, isolated from Arctic lichen (*Ochrolechia* sp.). *J. Bacteriol.* **194**, 1607-1607 (2012).
59. Shin, S.C., Kim, S.J., Ahn, D.H., Lee, J.K. & Park, H. Draft genome sequence of *Sphingomonas echinoides* ATCC 14820. *J. Bacteriol.* **194**, 1843-1843 (2012).
60. Tabata, M., Ohtsubo, Y., Ohhata, S., Tsuda, M. & Nagata, Y. Complete genome sequence of the γ-Hexachlorocyclohexane-degrading bacterium *Sphingomonas* sp. strain MM-1. *Genome Announc* **1**, e00247-13 (2013).
61. Videira, P.A., Cortes, L.L., Fialho, A.M. & Sa-Correia, I. Identification of the *pgmG* gene, encoding a bifunctional protein with phosphoglucomutase and phosphomannomutase activities, in the gellan gum-producing strain *Sphingomonas paucimobilis* ATCC 31461. *Appl. Environ. Microbiol.* **66**, 2252-2258 (2000).
62. Wang, X., Tao, F., Gai, Z., Tang, H. & Xu, P.Genome sequence of the welan gum-producing strain *Sphingomonas* sp. ATCC 31555. *J. Bacteriol.* **194**, 5989-5990 (2012).
63. Xue, C., *et al.* Draft genome sequence of *Sphingobium* sp. strain BHC-A, revealing genes for the degradation of hexachlorocyclohexane. *Genome Announc.* **2**, e00254-14 (2014).
64. Yabuuchi, E., *et al*. *Sphingomonas paucimobilis* gen. nov. and comb. nov., *Sphingomonas parapaucimobilis* sp. nov., *Sphingomonas yanoikuyae* sp. nov., *Sphingomonas adhaesiva* sp. nov., *Sphingomonas capsulata* comb. nov., and two genospecies of the genus *Sphingomonas*. *Microbiology and Immunology*. **34**, 99-119 (1990).
65. Yoon, J.H., Lee, C.H., Yeo, S.H. & Oh, T.K. *Sphingopyxis baekryungensis* sp nov., an orange-pigmented bacterium isolated from sea water of the Yellow Sea in Korea. *Int. J. Syst. Evol. Microbiol.* **55**, 1223-1227 (2005).
66. Zeng, Y., Feng, F., Liu, Y., Li, Y. &Koblížek, M. Genome sequences and photosynthesis

gene cluster composition of a freshwater aerobic anoxygenic phototroph, *Sandarakinorhabdus* sp. strain AAP62, isolated from the Shahu Lake in Ningxia, China. *Genome Announc.* **1**, e00034-13 (2013)

67. Zeng Y*, et al.* Whole-genome sequences of an aerobic anoxygenic phototroph, *Blastomonas* sp. strain AAP53, isolated from a freshwater desert lake in Inner Mongolia, China. *Genome Announc.* **1**, e0007113 (2013).

68. Zhao, N., Bai, Y., Zhao, X.Q., Yang, Z.Y. & Bai, F.W. Draft genome sequence of the flocculating *Zymomonas mobilis* strain ZM401 (ATCC 31822). *J. Bacteriol.* **194**, 7008-7009 (2012).

69. Zheng, Q., Zhang, R. & Jiao, N. Genome sequence of *Citromicrobium* strain JLT1363, isolated from the South China Sea. *J. Bacteriol.* **193**, 2074-2075(2011).

70. Zipper, C., Nickel, K., Angst, W. & Kohler, H.P.E. Complete microbial degradation of both enantiomers of the chiral herbicide mecoprop (RS)-2-(4-chloro-2-methylphenoxy)propionic acid in an enantioselective manner by *Sphingomonas herbicidovorans* sp. nov. *Appl. Environ. Microbiol.* **62**, 4318-4322 (1996)
